# Whole‐genome bisulfite sequencing analysis of circulating tumour DNA for the detection and molecular classification of cancer

**DOI:** 10.1002/ctm2.1014

**Published:** 2022-08-23

**Authors:** Yibo Gao, Hengqiang Zhao, Ke An, Zongzhi Liu, Luo Hai, Renda Li, Yang Zhou, Weipeng Zhao, Yongsheng Jia, Nan Wu, Lingyu Li, Jianming Ying, Jie Wang, Binghe Xu, Zhihong Wu, Zhongsheng Tong, Jie He, Yingli Sun

**Affiliations:** ^1^ Central Laboratory National Cancer Center/National Clinical Research Center for Cancer/Cancer Hospital & Shenzhen Hospital, Chinese Academy of Medical Sciences and Peking Union Medical College Shenzhen China; ^2^ Department of Thoracic Surgery National Cancer Center/National Clinical Research Center for Cancer/Cancer Hospital Chinese Academy of Medical Sciences and Peking Union Medical College Beijing China; ^3^ State Key Laboratory of Molecular Oncology National Cancer Center/National Clinical Research Center for Cancer/Cancer Hospital Chinese Academy of Medical Sciences and Peking Union Medical College Beijing China; ^4^ Key Laboratory of Genomic and Precision Medicine China Gastrointestinal Cancer Research Center Beijing Institute of Genomics Chinese Academy of Sciences Beijing China; ^5^ Department of Breast Oncology Tianjin Medical University Cancer Institute and Hospital National Clinical Research Center for Cancer Key Laboratory of Breast Cancer Prevention and Therapy Ministry of Education Key Laboratory of Cancer Prevention and Therapy Tianjin Medical University Tianjin China; ^6^ Department of Orthopedic Surgery Peking Union Medical College Hospital Chinese Academy of Medical Sciences and Peking Union Medical College Beijing China; ^7^ Laboratory of Translational Medicine National Cancer Center/National Clinical Research Center for Cancer/Cancer Hospital, Chinese Academy of Medical Sciences and Peking Union Medical College Beijing China

**Keywords:** cancer early detection, circulating tumour DNA, DNA methylation, epigenetic biomarkers, liquid biopsy, whole‐genome bisulfite sequencing

## Abstract

**Background:**

Cancer cell–specific variation and circulating tumour DNA (ctDNA) methylation are promising biomarkers for non‐invasive cancer detection and molecular classification. Nevertheless, the applications of ctDNA to the early detection and screening of cancer remain highly challenging due to the scarcity of cancer cell–specific ctDNA, the low signal‐to‐noise ratio of DNA variation, and the lack of non‐locus‐specific DNA methylation technologies.

**Methods:**

We enrolled three cohorts of breast cancer (BC) patients from two hospitals in China (BC: *n* = 123; healthy controls: *n* = 40). We developed a ctDNA whole‐genome bisulfite sequencing technology employing robust trace ctDNA capture from up to 200 μL plasma, mini‐input (1 ng) library preparation, unbiased genome‐wide coverage and comprehensive computational methods.

**Results:**

A diagnostic signature comprising 15 ctDNA methylation markers exhibited high accuracy in the early (area under the curve [AUC] of 0.967) and advanced (AUC of 0.971) BC stages in multicentre patient cohorts. Furthermore, we revealed a ctDNA methylation signature that discriminates estrogen receptor status (Training set: AUC of 0.984 and Test set: AUC of 0.780). Different cancer types, including hepatocellular carcinoma and lung cancer, could also be well distinguished.

**Conclusions:**

Our study provides a toolset to generate unbiased whole‐genome ctDNA methylomes with a minimal amount of plasma to develop highly specific and sensitive biomarkers for the early diagnosis and molecular subtyping of cancer.

## Graphical abstract

An improved ctDNA whole‐genome bisulfite sequencing method was developed to generate unbiased whole‐genome ctDNA methylomes using as low as 1 ng ctDNA.

A diagnostic signature with ctDNA methylation biomarkers demonstrated high accuracy in early and advanced breast cancer in multicentre patient cohorts.

The ctDNA methylome could distinguish the estrogen receptor status of breast cancers.

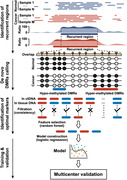

## 1 INTRODUCTION

Cancer causes the leading threat of death worldwide.^1^ The cancer screening and early diagnosis significantly decrease the mortality rate, as the timely detected cancer can be remedied by milder therapeutics or removed via surgery. Screening involves testing a healthy population to identify asymptomatic individuals with cancers. Conversely, early diagnosis focuses on classifying symptomatic patients as early as possible. Notably, screening requires the collection of samples from a large population, whereas early diagnosis requires periodic testing, which is only feasible using non‐invasive methods. Clinical methods for the non‐invasive detection of cancer include medical imaging technologies (such as X‐ray imaging,^2^ computed tomography,^3^ magnetic resonance imaging,^4^ ultrasonic testing^5^ and positron emission tomography‐computed tomography^6^) and serum antigen protein markers.^7^ These methods have their strengths; for example medical imaging reveals the location and morphology of tumours, whereas serum antigen protein markers have broad applications. However, these diagnostic techniques have limitations. For example, there is usually a lag between medical imaging and tumour progression, and such techniques may induce harm when using high‐energy rays and contrast agents.^8^ Additionally, serum markers would underestimate the heterogeneity of tumours, leading to rising misdiagnosis rates.^9^ Thus, conventional methods are not suitable for an early diagnosis of tumours, and there are urgent and unmet needs for the exploration of novel early tumour diagnostic markers of non‐invasive sampling, high sensitivity and specificity.

Tumour cells secrete single‐ or double‐stranded DNA fragments called circulating tumour DNA (ctDNA) to blood, offering a novel diagnostic tool.^10^, ^11^ ctDNA exhibits several distinct advantages: (i) Blood collection for ctDNA analysis is quick and simple. (ii) The half‐life of ctDNA (∼2 h) enables its use for the dynamic and real‐time monitoring of cancer progression. (iii) ctDNA detection reduces the bias associated with intratumoural genetic heterogeneity. (iv) ctDNA can detect the recurrence risk several months ahead of medical imaging. Thus, many ongoing studies apply ctDNA as a non‐invasive biomarker for the early diagnosis of tumours.^12^, ^13^, ^14^, ^15^, ^16^, ^17^, ^18^, ^19^, ^20^, ^21^ Genetic mutations of cancer‐associated genes are attractive candidates, which could be analysed in plasma‐derived ctDNA from cancer patients by established methodologies.^17^ Panel‐based sequencing provided more mutation detection opportunities. Nevertheless, there are three main challenges to this approach in cancer screening: (i) For early‐stage cancers, caps on acceptable phlebotomy volumes and limited ctDNA shedding may impact the sensitivity. (ii) The contamination of background DNA from white cells and mutations in plasma ctDNA from non‐malignant or premalignant processes (e.g. age‐related clonal haematopoiesis) may affect the test specificity. (iii) Mutations are not tissue specific, which brought the uncertainty of the origin of cancer. Therefore, there remains an urgent need to find a novel, practical method to overcome these limitations.

DNA methylation changes may be early events in the initiation and development of tumours, making them promising biomarkers for early cancer diagnosis.^22^, ^23^, ^24^, ^25^ Studies have demonstrated that particular DNA methylation signatures are better able to predict the risk of breast cancer (BC) than copy number variants in tissues.^18^ Furthermore, the DNA methylation profile obtained by the Human Methylation 450 (HM450K) microarray was able to classify central nervous system cancers with an accuracy exceeding that of histopathology using tissue.^20^, ^25^ However, the methods used in previous studies only cover 0.1%–1% of the genome, and cancer‐specific changes in methylation are easily missed, which markedly impacts the specificity and sensitivity of the technology. Reduced representation bisulfite sequencing (RRBS)^26^ and methylated DNA immunoprecipitation sequencing (MeDIP‐seq)^27^ have been used to seek an improved method for detecting DNA methylation. RRBS and MeDIP‐seq significantly improved DNA methylation coverage to 10% of the whole genome and were able to distinguish various types of cancers. Nevertheless, RRBS and MeDIP‐seq are based on enzyme digestion and antibody immunoprecipitation, respectively, resulting in locus‐specificity of the obtained data. Low‐pass whole‐genome bisulfite sequencing (WGBS) was also performed to reduce the cost of WGBS sequencing with a low depth (∼5 million reads) of ctDNA WGBS sequencing,^28^ which could perfectly cover the GC‐rich region. However, a high coverage of the whole genome, especially the GC‐poor region, still requires high‐depth sequencing, as we described here. Therefore, the coverage of CpG regions is limited, and information with low CG contents is omitted in DNA methylation profiling, leading to the loss of cancer‐specific messages. To overcome this limitation, the bisulfite sequencing of DNA has been developed.^29^ Bisulfite treatment converts unmodified cytosines in DNA to uracil, while maintaining 5‐methylcytosine (5mC). With PCR amplification, followed by sequencing, this can reach the single base resolution. Further, bisulfite treatment was performed together with next‐generation sequencing (NGS) yielded the WGBS data on the global genomic distribution of 5mC, with over 70% genome coverage.^30^ Additionally, the ctDNA concentration in plasma is extremely low. Approximately 2000 ng genomic DNA is required for the preparation of a WGBS library, which significantly exceeds the level of ctDNA in clinically available plasma samples.

**FIGURE 1:**
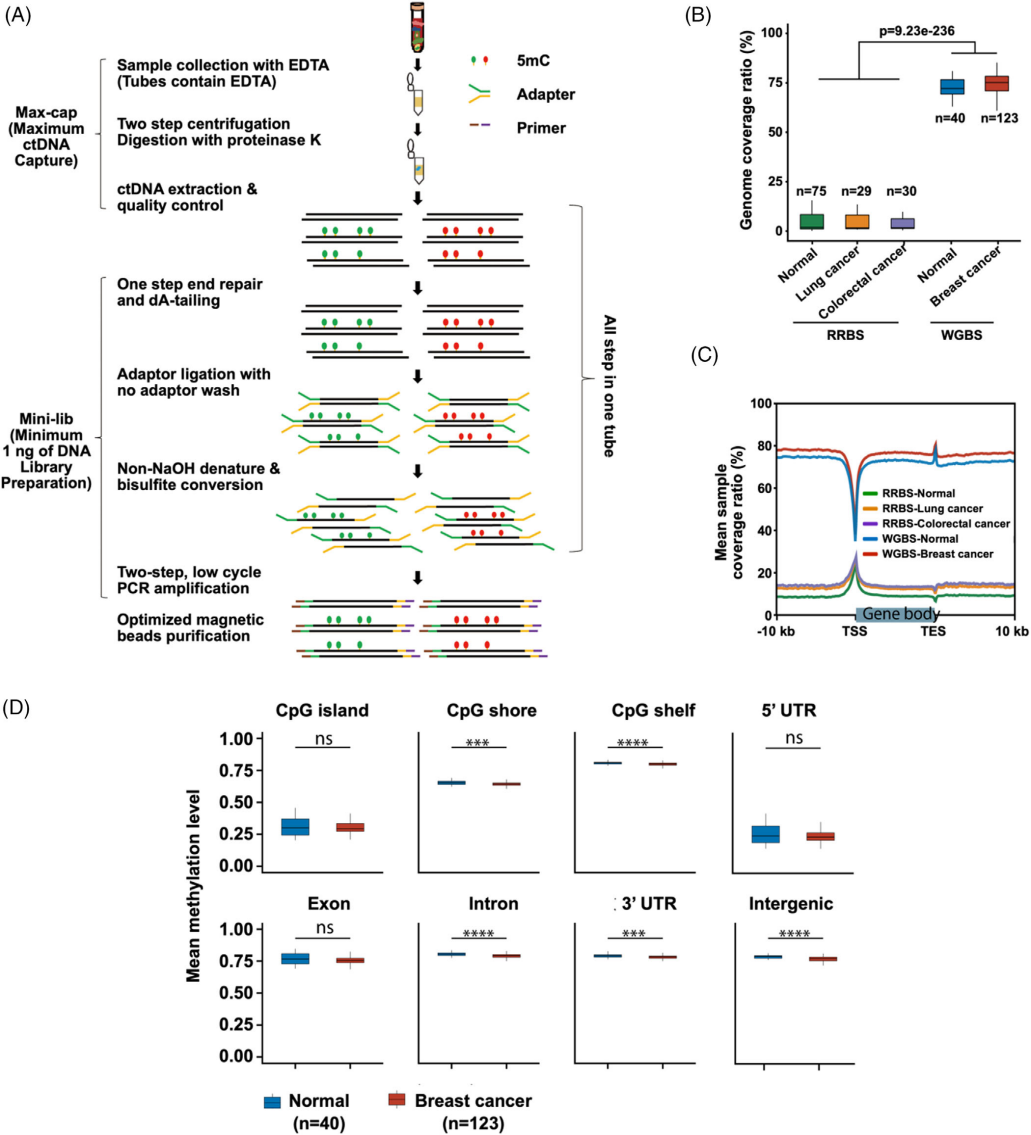
Workflow chart for data generation and analysis via sequencing of 5‐methylcytosine (5mC) in circulating tumour DNA (ctDNA). (A) Whole‐genome methylation sequencing of 5mC in ctDNA. ctDNA is extracted from plasma. Purified ctDNA is ligated with an adapter and bisulfite‐converted. The fragments were completed using PCR amplification followed by beads capture. (B) The breadth of reduced representation bisulfite sequencing (RRBS) and whole‐genome bisulfite sequencing (WGBS) data occupies ∼10% and ∼75% of the genome, respectively. (C) The sample coverage ratio of WGBS was higher than that of RRBS data. (D) The mean global methylation level of ctDNA in normal samples was higher than that in cancer samples.

Herein, an improved ctDNA–WGBS method was reported to accurately profile whole‐genome methylation patterns from trace quantities of ctDNA, which was extracted from only 200 μL of plasma, compared with the standard amount of 5–20‐mL plasma. Details of this process are demonstrated in Figure 1A. Considering the prevention of material losses and the low ctDNA requirement, the processes of end repair, dA tailing, adapter ligation and bisulfite conversion were performed in one tube. Additionally, beads were used for capture instead of agarose gel to substantially increase the recovery ratio. This novel ctDNA–WGBS method was applied for whole‐genome‐wide detection of 5mC at single‐base resolution in ctDNA of early‐stage cancer patients. This method enabled early‐stage BC detection with high specificity and sensitivity in multicentre patient cohorts due to the minimal input (as low as 1 ng) library preparation, unbiased genome‐wide coverage and comprehensive computational methods, which reduced the noise of low recurrent fragments and non‐tumour originating ctDNA. Moreover, the method was able to distinguish among molecular subtypes of cancer, which carry subtle differences in DNA methylation patterns.

## 2 RESULTS

### 2.1 WGBS library preparation with 1 ng ctDNA input yielded optimum library quality and high genome coverage, outperforming existing ctDNA methylation library construction methods

The improved protocol was first tested using samples from 123 BC patients (early stage: *n* = 53; advanced stage: *n* = 70) and 40 normal controls. Four pairs of BC tissues were constructed to generate WGBS data for the identification of ctDNA from tumour tissues. The concentration and quality of ctDNA from each sample are provided in Tables S1 and S2. The results showed that 163 high‐quality ctDNA‐WGBS profiles and 8 primary tissue DNA WGBS profiles were obtained. For both ctDNA and tissue DNA, CpG loci accounted for more than 65% of the whole genome (Table S3). The obtained WGBS data were then compared to previously reported RRBS data.^22^ As shown in Figure 1B, the breadth of the RRBS and WGBS data was ∼10% and ∼75% of the whole genome, respectively. The genome coverage ratio of the WGBS data was more widespread than that of the RRBS data (Figure 1C). Additionally, the mean levels of global ctDNA and tissue methylation were higher in normal samples than in cancer samples (Figures 1D and S1).

**FIGURE 2:**
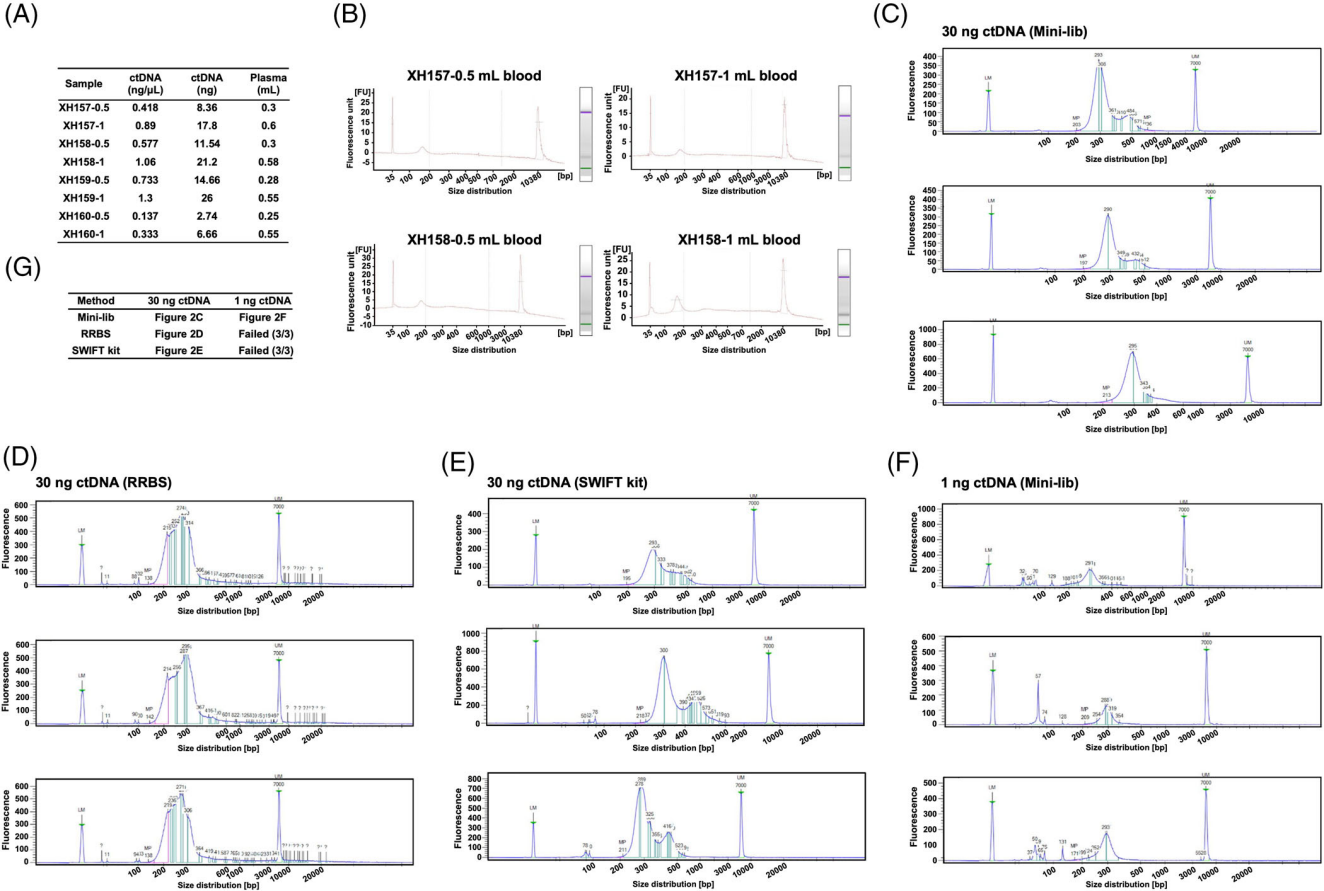
High‐quality circulating tumour DNA (ctDNA) extracted with Max‐cap and the library product of the Mini‐lib whole‐genome bisulfite sequencing (WGBS) library preparation. (A) The amount of ctDNA extracted with Max‐cap from 0.5 mL (∼200 μL plasma) and 1 mL whole blood in different samples. The experiment was performed in triplicate. (B) Agilent 2100 Bioanalyzer results show that ctDNA extracted with Max‐cap from 0.5 mL (∼200 μL plasma) and 1 mL blood is enriched around 160–180 bps. (C)–(E) An analysis of library prepared with Mini‐lib from 30 ng ctDNA input with LabChip GX touch (head‐to‐head experiments with Mini‐lib, reduced representation bisulfite sequencing [RRBS], and SWIFT kit). (F) An analysis of library prepared with Mini‐lib from 1 ng ctDNA input with LabChip GX touch. (G) In comparison with other advanced library preparation methods, only Mini‐lib enabled 1 ng input ctDNA library preparation for WGBS (head‐to‐head experiments with Mini‐lib, RRBS and SWIFT kit; note that the latter two methods failed with 1 ng input DNA, and no product was available). The experiment was performed in triplicate. The minimum input of ctDNA for use with the SWIFT kit and RRBS is around 30 ng.

Approximately 10 ng of ctDNA could be obtained in 0.5 mL of whole blood (around 200 μL plasma) from patients with advanced cancer (Figure 2A). The test performed by an Agilent 2100 Bioanalyzer revealed that the quality of ctDNA extracted from 0.5 mL of whole blood was equivalent to that extracted from 1 mL of whole blood and was enriched around 160–180 bps (Figure 2B). This demonstrated the reliability of our method. Furthermore, we collected library preparations for DNA methylation sequencing and selected those that claimed to generate a ctDNA library with less than 100 ng of input ctDNA, including RRBS and another method reported in several recent papers using SWIFT Accel‐NGS@Methyl‐Seq DNA Library kits (referred to as ‘SWIFT kits’ herein).^22^, ^31^, ^32^ Head‐to‐head library preparation using triplicate experiments from the same advanced sample showed that the minimum input ctDNA for both the RRBS and SWIFT kits was ∼30 ng. With 30 ng of input ctDNA, the minimum 1 ng of DNA Library Preparation (Mini‐lib) (Figure 2C), RRBS (Figure 2D) and SWIFT kits (Figure 2E) presented an enriched peak of around 300 bps. However, with 1 ng of input ctDNA, only the Mini‐lib generated clear peaks at ∼300 bps and exhibited excellent performance in subsequent sequencing tasks (Figure 2F). Both the RRBS and SWIFT kits failed to generate a ctDNA library (Figure 2G). Taken together, these data indicate that Mini‐lib provides a powerful tool for generating a WGBS library with input ctDNA as low as 1 ng.

ctDNA methylation libraries were also constructed from the same patient sample using RRBS, single‐cell WGBS (sc‐WGBS) and ctDNA‐WGBS. A head‐to‐head comparison revealed that sc‐WGBS covered less than 15% of the genome. Using our method, the sequence was able to cover more than 70% of the genome. Additionally, the ctDNA‐WGBS methylome covered most markers, whereas the sc‐WGBS methylome only covered one third of the ctDNA‐WGBS methylome, and the RRBS methylome covered no cancer‐specific markers (Figure S2).

### 2.2 Optimized deep‐learning algorithm revealed cancer‐specific recurrent regions

**FIGURE 3:**
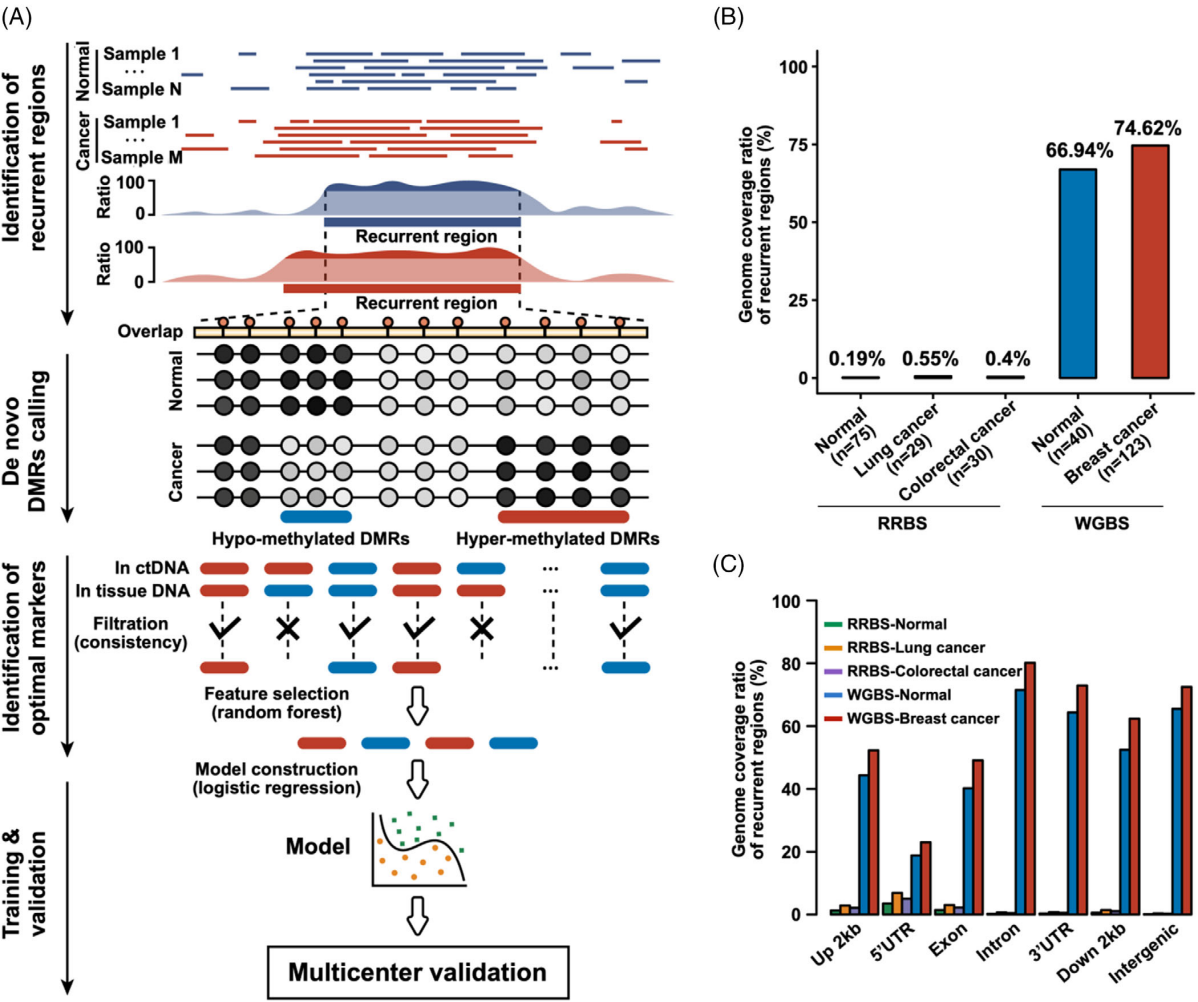
Computational workflow analysis of recurrent regions. (A) Workflow showing the processing of recurrent regions. (B) and (C) Bar plots showing the identified recurrent regions on the whole‐genome scale for reduced representation bisulfite sequencing (RRBS) and whole‐genome bisulfite sequencing (WGBS), respectively

Next, we investigated recurrent regions, termed differential methylation regions (DMRs), which are associated with greater stability and reliability. The computational workflow for the analysis of recurrent regions is shown in Figure 3A. First, recurrent regions were identified using normal and cancer samples. Next, hypo‐ and hypermethylated DMRs were determined in the recurrent regions of cancer patients and compared with normal people. Optimal biomarkers were then identified, and cancer tissue was used to generate the WGBS data and to filter the level of consistency in tumour tissue to ensure a uniform amount of DNA input for ctDNA samples. Next, random forest algorithms and logistic regression were used for feature selection. Finally, the model was constructed and validated using multicentre data, and recurrent regions were compared on the whole‐genome scale using the RRBS and WGBS methods. The genome coverage ratio of the recurrent regions exceeded 66.94% using our improved WGBS method. Nevertheless, the recurrent regions for RRBS were less than 1% (Figure 3B). Compared to the RRBS data for gene distribution, our WGBS data demonstrated extensive coverage across the whole genome, indicating that more genomic regions were used for subsequent biomarker identification (Figure 3C).

### 2.3 High genome coverage ctDNA methylome enabled the sensitive detection of tumours

**FIGURE 4:**
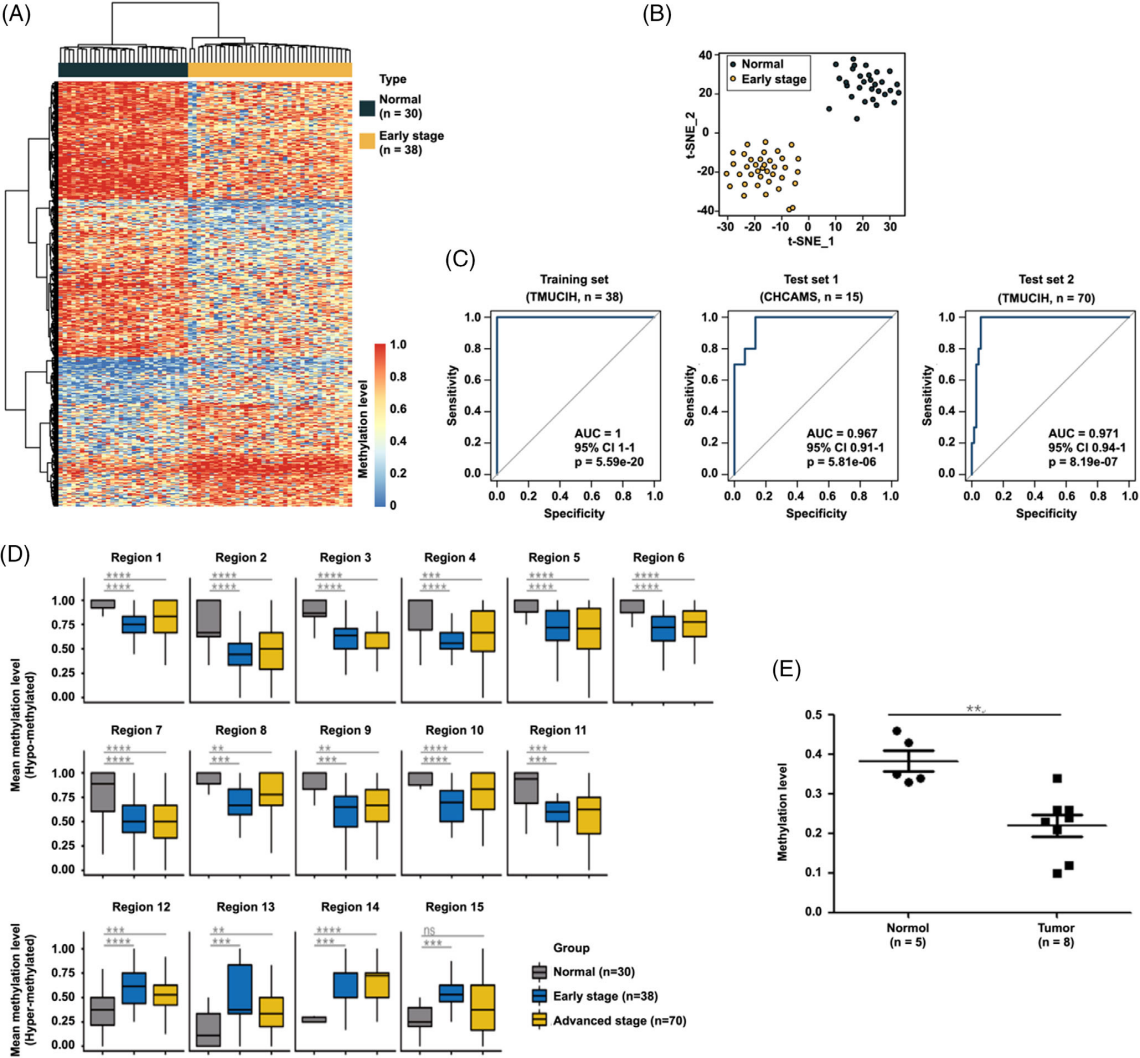
Early detection of breast cancer using circulating tumour DNA (ctDNA) differential methylation regions (DMRs). Heat map (A) and *t*‐stochastic neighbour embedding (*t*‐SNE) plot (B) showing the clustering of healthy individuals and patients with early‐stage breast cancer using 583 differentially methylated regions on ctDNA. (C) Receiver operating characteristic curves suggest the classification of healthy individuals and patients with early‐stage breast cancer in the independent training and testing datasets in the two cohorts. The receiver operating characteristic curves suggested the classification of normal individuals and patients with early‐ and advanced‐stage breast cancer in training data and other independent testing datasets using the same 15 differentially methylated regions with potential for biomarkers. (D) Box plot showing the position of 15 potential markers. The mean methylation distribution of 15 optimal ctDNA DMRs biomarkers consisted of 4 hypermethylated and 11 hypomethylated biomarkers in normal samples and early‐ and advanced‐stage breast cancer samples, respectively. Centrelines show the median; boxes represent the interquartile range (25%–75%); whiskers correspond to 1.5 times the interquartile range. *p‐*Value was computed using a two‐tailed Student's *t*‐test. ns, *p* > 0.05; **p ≤ 0.01; ***p ≤ 0.001; ****p ≤ 0.0001. (E) The vertical scatter plot showing the methylation level of ctDNA from healthy controls and breast cancer patients

Based on these encouraging outcomes, we next identified 583 DMRs (Table S4) from ctDNA among normal people (*n* = 30) and early stage BC patients (*n* = 38). The average length of the obtained ctDNA DMRs was ∼82 bp (Figure S3A). In addition, these DMRs were chiefly enriched in the intron and intergenic regions (Figure S3B). The hierarchical clustering results precisely classified patients and healthy individuals by the DMRs methylation levels in the training set (Figure 4A). *t*‐Stochastic neighbour embedding (*t*‐SNE) analysis revealed a similar clear classification in the testing dataset (Figure 4B).

Considering the tissue specificity of DNA methylation, we identified 58 of these 583 DMRs with trends in ctDNA methylation levels that were consistent with those in tissue DNA. Next, the random forest algorithm was applied for feature selection, resulting in 15 DMRs (Table S5) as potential early BC diagnosis biomarkers. We used multicentre samples to test the obtained biomarkers and recruited three Chinese BC cohorts in two hospitals, as follows: a training set consisting of early/non‐metastatic BC samples recruited from the Tianjin Medical University Cancer Institute and Hospital (TMUCIH). The Test set 1 comprised early/non‐metastatic BC samples from the Cancer Hospital, Chinese Academy of Medical Sciences (CHCAMS). The Test set 2 included advanced/metastatic BC samples recruited from TMUCIH. Detailed clinical information and demographic characteristics of these patients are shown in Tables S6 and S7. The receiver operating characteristic (ROC) curves demonstrated that the sensitivity, specificity and area below the ROC curve (AUC) were 100%, 100% and 1 for Training set; 87%, 100% and 0.967 for Test set 1; and 94%, 100% and 0.971% for Test set 2, respectively (Figures 4C, S4 and S5). The positions of 15 potential markers are shown in the box plot in Figure 4D. The mean methylation distribution of 15 optimal ctDNA DMRs biomarkers comprised 4 hyper‐ and 11 hypomethylated biomarkers in normal, early stage and advanced stage BC samples. As it is very important to use other easily accessed methods for clinical translation, we performed droplet digital PCR (ddPCR) to double‐check the marker panel that we found by ctDNA‐WGBS (Figure 4E and Table S8).

In addition, we further validated the as‐obtained early diagnostic biomarkers by independent datasets from The Cancer Genome Atlas (TCGA) HM450K. In total, 34 of 583 DMRs were reproducible in the TCGA dataset, and we evaluated their performance in the TCGA dataset, including 98 normal adjacent tissues and 785 early‐stage BC tumour tissues (Table S9). The ROC curves demonstrated that the AUC was 0.996, indicating that ctDNA DMRs obtained from plasma were consistent with those obtained from primary tumours (Figure S6).

### 2.4 Different cancer types and subtypes could be discriminated using the methylomes of ctDNA

**FIGURE 5:**
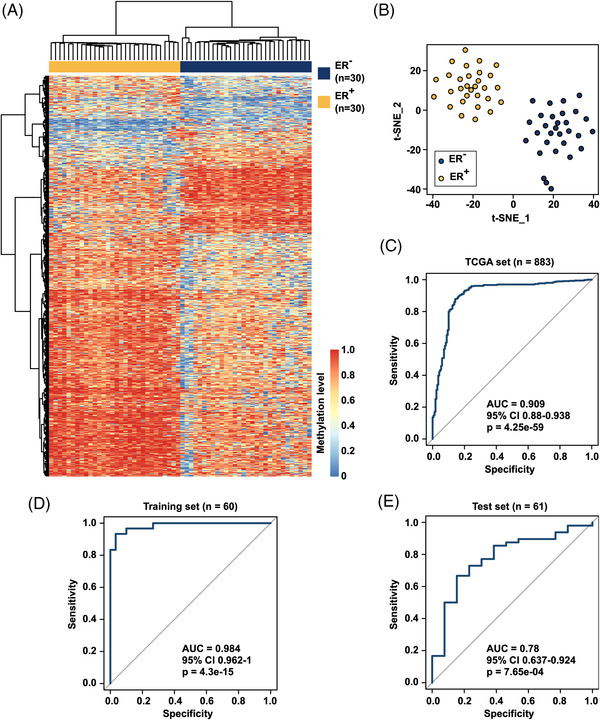
Circulating tumour DNA (ctDNA) methylation as potential biomarkers for the subtype classification of breast cancer and prediction of ER status. Heat map (A) and *t*‐stochastic neighbour embedding (*t*‐SNE) (B) plot of 1332 differential methylation regions (DMRs) between ER^+^ and ER^−^ breast cancer samples. (C) External validation of 1332 DMRs using The Cancer Genome Atlas (TCGA) 450 K data. In total, 47 of 1332 DMRs were reproducible in TCGA data, and the receiver operating characteristic curve exhibited good discrimination ability between ER^+^ and ER^−^ breast cancer samples. Receiver operating characteristic curves of a predictive model comprising 12 markers in the Training set (D) and independent Test set (E) (Training set: 30 ER^+^ breast cancer samples and 30 ER^−^ breast cancer samples; Test set: 48 ER^+^ breast cancer samples and 13 ER^−^ breast cancer samples)

Encouraged by the high specificity and sensitivity of early stage BC screening, we further evaluated the discriminating ability of our method to different subtypes of BC. The BC patients were evaluated based on ctDNA DMRs using 30 estrogen receptor‐negative (ER^−^) and 30 ER‐positive (ER^+^) patients with the described computational framework. Eventually, 1332 ctDNA DMRs were identified (Table S10), and both hierarchical clustering (Figure 5A) and *t*‐SNE (Figure 5B) analyses obtained a clear classification of these two BC subtypes. Moreover, the identified DMRs were subject to external validation using independent TCGA 450 K data. In total, 47 of 1332 DMRs were reproducible in the TCGA dataset, and we evaluated their performance on the TCGA dataset, including 570 ER^+^ and 169 ER^−^ BC tissues. In Figure 5C, the ROC data demonstrated that the CpG site methylation levels in ctDNA DMRs could effectively distinguish primary tumour tissues with distinct ER states (AUC = 0.909). A random forest algorithm was applied for feature selection, resulting in 12 ctDNA DMRs as potential markers. To further assess the capability of as‐obtained ctDNA DMRs to distinguish the clinical subtypes of BC, we randomly divided the BC patients into two almost equal‐sized subsets to train a model (using binary logistic regression as described earlier) and tested the model. As displayed in Figures 5D,E, S7 and S8, the results suggested that the predictive model comprising 12 biomarkers could distinguish the two BC subtypes in the Training set (AUC = 0.984, sensitivity: 93%, specificity: 93%) and independent Test set (AUC = 0.780, sensitivity: 73%, specificity: 87%). Collectively, these results indicated that changes in ctDNA methylation in BC could be used to discriminate the ER status of patients. To investigate the potential of our established method for subtyping cancers, different types of cancers were further investigated. Importantly, the *t*‐SNE analysis revealed that BC, hepatocellular carcinoma, lung cancer and normal control samples could be well distinguished using this method (Figure S9). Although further research is required, the previous data indicated that our method provides a toolset that can be used to develop highly sensitive and specific biomarkers for discriminating different types and subtypes of cancer.

## 3 DISCUSSION

Epigenetic abnormalities can lead to tumourigenesis, and recent works showed that DNA methylation could be a promising biomarker for different types of tumours.^21^, ^22^, ^33^, ^34^ Detecting DNA methylation provides advantages over detecting copy number variants and somatic mutations.^18^ However, there are many obstacles to its clinical application. Detection sensitivity and specificity are critical for identifying cancer, especially in its early stages. Thus, it is critical to cover as many genome sequences as possible to obtain reliable sensitivity and specificity results. Recent research has concentrated on the application of RRBS to detect DNA methylation in different types of tumours.^21^, ^22^, ^33^, ^34^ Although data have been obtained with RRBS,^26^ MeDIP‐seq^27^ and low‐pass WGBS,^28^ researchers have continued to search for an ‘unbiased’ method of detecting DNA methylation that can cover more genomes than RRBS or MeDIP‐seq.

WGBS was selected as a promising method to detect ctDNA methylation because it can cover the highest genome ratio among all methods of DNA methylation detection. However, when applied with ctDNA detection, current WGBS research was not found to outperform other methods as expected.^33^, ^34^, ^35^ Notably, WGBS requires a large amount of input DNA for library preparation, usually 2000 ng, and 5 mL of whole blood generally contains 10–50 ng ctDNA (in early stages of cancer).^19^, ^35^ Thus, it was critical to develop a method, such as the ctDNA‐WGBS method, using trace amounts of input DNA for library preparation. In the present study, an improved ctDNA–WGBS method was reported to accurately profile whole‐genome methylation patterns from trace amounts of ctDNA obtained from plasma. This ctDNA–WGBS method enables us to detect ctDNA levels as low as 1 ng in plasma, which increases the sensitivity of WGBS detection from 18%^35^ to 87%.

Data analysis was also optimized by filtering the background signals. Our data were shown to cover more regions than the RRBS for both normal samples and those from patients with cancer. The obtained methylomes contained a higher amount of methylation information for ctDNA than that from RRBS, resulting in the identification of 15 specific biomarkers, which may be missed by RRBS or other methods. Overall, we demonstrated that ctDNA methylation could identify BC in both early and advanced stages with high specificity and sensitivity. We verified the reliability of biomarkers by ddPCR, which is a cost‐effective method and can be performed for high‐throughput clinical detection in the future.

Most of the fragmented DNA in plasma is cell‐free DNA (cfDNA), which is released by apoptotic cells from different tissues. ctDNA is part of the cfDNA, and only 1%–10% of the cfDNA is ctDNA. It is very important to reduce the background of cfDNA to search for cancer‐specific biomarkers.^36^ First, we chose to investigate methylation markers because methylation is tissue‐specific. Second, we carefully performed extraction to ensure the ∼160 bp ctDNA product. Third, we performed the previous data analysis and filtered the data with cancer tissue data to minimize the background cfDNA background noise.

Previous studies have shown that DNA methylation patterns can help distinguish different types of cancers.^20^, ^21^, ^22^, ^35^, ^37^ Here, we have revealed that ctDNA–WGBS is sensitive enough to distinguish different subtypes of BC. Overall, our findings demonstrated that overcoming the technical limitations of ctDNA–WGBS enabled us to detect ctDNA methylation signals in concentrations as low as 1 ng. The combination of deep learning and data analysis may provide a powerful and sensitive whole‐genome coverage tool for early‐stage cancer detection and subtype classification. With the NGS cost decreasing, it is hoped that the ctDNA–WGBS approach will become increasingly accessible for both basic and clinical research.

## 4 CONCLUSIONS

In summary, an improved ctDNA–WGBS method was reported to accurately profile whole‐genome methylation patterns from trace amounts of ctDNA, which was extracted from only 200 μL of plasma. This method enabled the early‐stage BC screening with high specificity and sensitivity in multicentre patient cohorts due to mini‐input (as low as 1 ng) library preparation, unbiased genome‐wide coverage and comprehensive computational methods, which reduced the noise of low recurrent fragments and non‐tumour‐originating ctDNA. Moreover, the protocol was effective at developing highly specific and sensitive biomarkers for distinguishing various types and subtypes of cancer. We anticipate that our established method for early diagnosis of cancer will have substantial clinical translation potential.

## 5 MATERIALS AND METHODS

### 5.1 Patient cohorts

We recruited three Chinese BC cohorts from two hospitals. The discovery set (Training set) consisted of female patients of Chinese descent with early/non‐metastatic BC from the TMUCIH (Dec 2016–Dec 2017). The first replication cohort (Test set 1) consisted of Chinese female patients with early/non‐metastatic BC recruited from the CHCAMS (May 2018–Oct 2018). The second replication case series (Test set 2) comprised female advanced/metastatic BC patients from TMUCIH. Specifically, 38 Chinese female early/non‐metastatic BC patients were recruited (mean age ± standard deviation [SD]: 50.87 ± 11.05) in the training set. For Test set 1, 15 Chinese female early/non‐metastatic BC patients were recruited (mean age ± SD: 53.47 ± 8.88). For Test set 2, 70 female advanced/metastatic BC patients were recruited (mean age ± SD: 50.7 ± 10.31). In total, 123 female BC patients were included. Age‐matched Chinese females without cancer were enrolled in the healthy control group from the Beijing Institute of Genomics. In addition, 40 females were divided into two control groups (*n* = 25 for Control group 1, and *n* = 15 for Control group 2).

### 5.2 Phenotype evaluation

The BC and metastasis diagnosis were established on pathological analysis. Based on the American Joint Committee on Cancer for BC staging (the eighth edition), pathological staging of the lymph node, primary tumour and metastasis was double‐checked and carefully defined and was further classified into stages I–IV.^38^ Immunohistochemistry (IHC) was applied to obtain the ER and progesterone receptor (PR) status. A tumour was defined as ER/PR negative if IHC results of tumour nuclei were less than 1%. However, when IHC analysis was borderline, fluorescence in situ hybridization was used to access human epidermal growth factor receptor 2 (HER2). According to the St Gallen 2017 criteria, the molecular subtypes were determined by HER2 and hormone receptor status.^39^

### 5.3 ctDNA extraction

DNA extracted from blood was stored in blood collection tubes (Streck, Omaha, NE). Plasma was obtained from blood using centrifugation for 10 min at 1900 × *g* and then for another 10 min at 18 000 × *g* with EDTA and proteinase K. Afterwards, the plasma samples were processed by the QIAamp Circulating Nucleic Acid Kit (55114, Qiagen, Valencia, CA). An average of 20–80 ng ctDNA could be obtained from ∼4–5 mL of plasma. As‐obtained samples were kept at −80°C prior to use.

### 5.4 Tissue DNA extraction

Fresh frozen cancer tissues were used to obtain genomic DNA by a QIAshredder (79654, Qiagen, Valencia, CA). An average of 2 μg genomic DNA was obtained from ∼0.3 mg of tissues. As‐obtained samples were kept at −80°C prior to use.

### 5.5 ctDNA methylation library

We used DNA to prepare methylation libraries for WGBS. First, ctDNA (1–10 ng) and control non‐methylated λ‐phage DNA (1/1000–5/1000 ng, D1521, Promega, Madison, WA) were blended. λ‐phage DNA was sheared to obtain 200 bp fragments with a Covaris S220 ultrasonicator (Covaris, Woburn, MA). Second, the NEBNext Ultra End II Repair/dA Tailing Module (E7442S/L, NEB, Ipswich, MA) reagents were used to synthesize the DNA fragments by end repair adenylation of the 3′‐ends (30 min@20°C for the end repair reaction, and 30 min@65°C for the dA tailing reaction). Third, the NEBNext Ultra II Ligation Module (E7445S/L, NEB, Ipswich, MA) reagents were applied to ligate the methylation adaptor (15 min@20°C). The adaptor sequences for NGS were obtained from Illumina. Then, the EZ DNA Methylation Gold Kit (D5005, Zymo Research, Orange, CA) was used to bisulfite‐converted DNA. Afterwards, amplify DNA by PCR as follows: 25 μL of KAPA HiFi HS Uracil+ ReadyMix (KK2801, KAPA, Wilmington, MA), 10 μM primer 1.0, 10 μM primer index and 20 μL DNA, under the following conditions: 45 s@98°C; 10–14 cycles of 15 s@98°C, 30 s@64°C, 30 s@72°C and 60 s@72°C. Finally, we used Agencourt AMPure XP beads (Beckman Coulter, Miami, FL) to select efficient captures (∼290 bp) as the resulting library. The Qubit dsDNA HS Assay Kit (Thermo Fisher, Waltham, MA) was used to analyse the library purity. The X‐ten system (Illumina, San Diego, CA) was used to sequence the final DNA methylation library.

The ctDNA methylation library was prepared with the SWIFT kit as described in the previous study.^31^, ^32^ Briefly, 5 ng of ctDNA and 100 ng of germline DNA were sonicated to 180–220 bp by the Covaris S220 ultrasonicator and bisulfite converted by the EZ DNA Methylation Gold Kit (D5005, Zymo Research, Orange, CA). Single‐stranded DNA was treated with the Accel‐NGS Methyl‐Seq DNA Library kit (36024, Swift Biosciences, Ann Arbor, MI) for library construction. Briefly, the Adaptase Module (Swift Biosciences, Ann Arbor, MI) was applied to incorporate truncated adapter sequences into single‐stranded DNA in template‐independent reactions step by step. Then, DNA was enriched via PCR using Illumina sequencing‐compatible primers for nine cycles for ctDNA and six cycles for genomic DNA. The RRBS library was prepared as described previously.^22^

### 5.6 Genomic DNA methylation library

Single‐cell DNA methylation WGBS (sc‐WGBS) and RRBS DNA methylation libraries were prepared as previously described.^22^ The X‐ten system (Illumina, San Diego, CA) was used to sequence DNA methylation libraries.

### 5.7 Quality analysis and WGBS data mapping

Raw sequencing data in FASTQ format were trimmed to delete low‐quality bases, amplification primers and sequencing adapters from the read ends. After quality control, Bismark was applied to map reads to the human reference genome (hg19) and remove PCR duplicates.^40^ The spike‐in of totally non‐methylated λ‐phage DNA was used to calculate the bisulfite conversion ratio. A BAM file containing only the mapped and duplicate deleted reads was applied for subsequent bioinformatics analyses.

### 5.8 Bioinformatics pipeline for cancer early detection

A rigorous and comprehensive computational workflow was developed to screen optimal ctDNA methylation biomarkers for the early detection of cancer from 163 high‐quality ctDNA‐WGBS profiles and 8 primary tissue DNA WGBS profiles.

1. Regions of ctDNA recurrence were identified in a population of two groups of BC patients and healthy people. To minimize the influence of missing values of ctDNA fragments in the sample cohorts, ctDNA recurrent regions within a population of samples were determined by the Poisson tests in BC ctDNA samples and healthy controls. The recurrence rate for each site was assessed through the percentage of samples covered by at least one read at that site. High confidence ctDNA recurrent regions were extracted using a stringent threshold with *p* < 0.01, and the ratio of recurrence at each site was >70%. Overlap among recurrent regions of normal ctDNA controls and those of BC ctDNA cases were selected as reference recurrent regions.
2. Identification of de novo differentially methylated regions. To identify precise and reliable ctDNA DMRs between healthy controls and cancer patients in the training set, de novo ctDNA DMRs calling was performed in the ctDNA reference recurrent regions. We identified de novo ctDNA DMRs based on changes in adjacent CpG‐methylated patterns using the previously reported CpG_MPs protocol with a rigid threshold for the absolute mean methylation difference of each region being >0.2 and *p* < 0.01.^41^
3. Identification of optimal ctDNA methylation markers of BC. To minimize the influence of methylation noise from other tissues in plasma, BC‐specific ctDNA DMRs were extracted from the primary tumour tissue WGBS samples. The consistency of methylation patterns between ctDNA DMRs in ctDNA and tissue was evaluated by the mean methylation difference. The ctDNA DMRs remained as cancer‐specific ctDNA methylation biomarkers due to the consistent absolute mean methylation difference >0.2. Moreover, a backward stepwise strategy identified the optimal ctDNA DMRs for use as cancer‐specific markers. All cancer‐specific ctDNA DMRs were ranked based on their importance score, and the least important features were iteratively discarded one by one. The random forest R package was applied to evaluate the importance scores.^42^ Finally, the predictive model was constructed based on the optimal 15 cancer‐specific ctDNA DMRs.
4. Model construction and validation. A random forest algorithm was used to construct the model by fitting 500 trees using the methylation levels of 15 markers selected as before. The ultimate model derived from the training set was applied to the test set for independent validation. For unseen test samples, we estimated the methylation level of the region by averaging that of all covered CpG sites within the region. Considering the importance attached to outliers, missing values were replaced with the median of all available methylation values in the corresponding group.

### 5.9 Data processing and analysis

WGBS data were processed for the ctDNA and tissue samples. Raw data in FASTQ format were filtered using trim_galore after sequencing. The filter reads were mapped to Bismark using the hg19 reference genome sequences.

Considering the low detection rate of ctDNA in some genomic regions, we first used DANPOS^43^ to identify highly recurrent regions in BC and normal ctDNA samples, respectively. Those with a recurrence rate greater than 70% and an adjusted *p*‐value <1 × 10^−10^ were identified as recurrent regions. Overlaps between those of normal ctDNA samples and the recurrent regions of BC ctDNA samples were extracted for further analysis.

By continuous scanning in the recurrent regions, segments comprising CpG sites with high methylation similarities across all samples were obtained using the genome segmentation function of SMART2.^44^ Segments with lengths greater than 10 bp and comprising more than three CpG sites were reserved to identify DMRs. Differential methylation between early‐stage cancer samples and normal samples was examined using a two‐tailed Student's *t*‐test and the absolute mean methylation difference. Only those segments with absolute mean methylation difference >0.2 and *p* < 0.05 were regarded as DMRs.

To reduce the number of false‐positive DMRs, four pairs of BC tissue DNA and normal tissue DNA samples were used. Only those DMRs with a significant absolute mean methylation difference (>0.2) between BC tissue DNA and normal tissue DNA and consistent directional change with ctDNA were retained for further analysis.

To avoid overfitting, a backward stepwise strategy was implemented to reduce the model complexity. Specifically, all features were sorted according to the importance score, and the least important features were iteratively discarded one by one. A random forest algorithm was applied to evaluate the importance scores. Finally, a predictive model was developed based on the optimal 15 features. The model was constructed by the least absolute shrinkage and selection operator‐penalized logistic regression.

### 5.10 ddPCR analysis

For ddPCR, droplets were generated based on the instruction manual to avoid the formation of bubbles, transferred into 96‐well microtitre plates and sealed. PCR amplification was performed in a thermal cycler with a 2°C/s ramp rate to avoid the breakage of oil droplets. The droplet reader (QX200, Bio‐Rad, Hercules, CA) was used to measure the fluorescence signals.

The methylated sequences were labelled with carboxyfluorescein (FAM), and the unmethylated sequences were labelled with hexachloro‐fluorescein (HEX). The specificity of the probe was detected by the EpiTect PCR Control DNA kit (Qiagen, 59695): methylated human control DNA as a template to detect the specificity of the FAM probe. Unmethylated human control DNA is considered a template to detect the specificity of the HEX probe. For each experiment, water was used as a blank control. The input amount of methylated template and unmethylated template was 0, 3, 5, 7 and 10 ng, respectively.

The temperature conditions (55, 55.7, 57, 59, 61.4, 63.3 and 64.5°C, respectively) were optimized, and the optimal temperature is 59°C. Then, the specificity and the concentration of the hybridization probes and the primers were optimized. The final concentration was 900 nM primers and 200 nM probes, 400 nM primers and 250 nM probes, 200 nM primers and 100 nM probes, and other conditions were optimized. Finally, 900 nM primers and 200 nM probes were used. According to the optimal concentration and temperature of the previous primers and probes, the biomarkers we found by ctDNA–WGBS were verified by ddPCR.

## CONFLICT OF INTEREST

The authors declare no competing interests.

## Supporting information



Supporting Information

## Data Availability

The scripts and data that support the findings of this study are openly available in GitHub at https://github.com/zhq921/cfWGBS‐bioinfo‐pip. The raw sequencing data reported in this manuscript are publicly available from the Genome Sequence Archive (http://gsa.big.ac.cn) under the accession number CRA001142 or from the National Center for Biotechnology Information (https://www.ncbi.nlm.nih.gov) under the accession number PRJNA494975. All the data and materials are available upon reasonable request.
