# Supplementary material for: Whole‐genome bisulfite sequencing analysis of circulating tumour DNA for the detection and molecular classification of cancer
Source: Clin Transl Med. 2022 Aug 23;12(8):e1014. doi: 10.1002/ctm2.1014 (PMC9398227; doi:10.1002/ctm2.1014)
Supplement: Supplementary file 1 — Supporting Information [file CTM2-12-e1014-s001.docx]

Supplementary Materials

of

**Whole-genome bisulfite sequencing analysis of circulating tumor DNA for the detection and molecular classification of cancer**

Yibo Gao^1,2,3#^, Hengqiang Zhao^6#^, Ke An^4#^, Zongzhi Liu^1,4#^, Luo Hai^1#^, Renda Li^2^, Yang Zhou^2^, Weipeng Zhao^5^, Yongsheng Jia^5^, Nan Wu^6^, Lingyu Li^1^, Jianming Ying^3^, Jie Wang^2,3^, Binghe Xu^2,3^, Zhihong Wu^6*^, Zhongsheng Tong^5*^, Jie He^1,2,3*^ and Yingli Sun^1,3,4*^

^1^National Cancer Center/National Clinical Research Center for Cancer/Cancer Hospital & Shenzhen Hospital, Chinese Academy of Medical Sciences and Peking Union Medical College, Shenzhen 518116, China

^2^Department of Thoracic Surgery, National Cancer Center/National Clinical Research Center for Cancer/Cancer Hospital, Chinese Academy of Medical Sciences and Peking Union Medical College, Beijing 100021, China

^3^State Key Laboratory of Molecular Oncology, National Cancer Center/National Clinical Research Center for Cancer/Cancer Hospital, Chinese Academy of Medical Sciences and Peking Union Medical College, Beijing 100021, China

^4^Key Laboratory of Genomic and Precision Medicine, China Gastrointestinal Cancer Research Center, Beijing Institute of Genomics, Chinese Academy of Sciences, Beijing 100101, China

^5^Department of Breast Oncology, Tianjin Medical University Cancer Institute and Hospital, National Clinical Research Center for Cancer, Key Laboratory of Breast Cancer Prevention and Therapy, Ministry of Education, Key Laboratory of Cancer Prevention and Therapy, Tianjin Medical University, Tianjin 300060, China

^6^Department of Orthopedic Surgery, Peking Union Medical College Hospital, Peking Union Medical College and Chinese Academy of Medical Sciences, Beijing 100730, China

*^*^Correspondence: scaroll99@hotmail.com (Y. Sun); hejie@cicams.ac.cn (J. He); tongzhongsheng@tjmuch.com (Z. Tong); wuzh3000@163.com (Z. Wu)*

*^#^* *Yibo Gao, Hengqiang Zhao, Ke An, Zongzhi Liu and Luo Hai contributed equally to this work.*

**
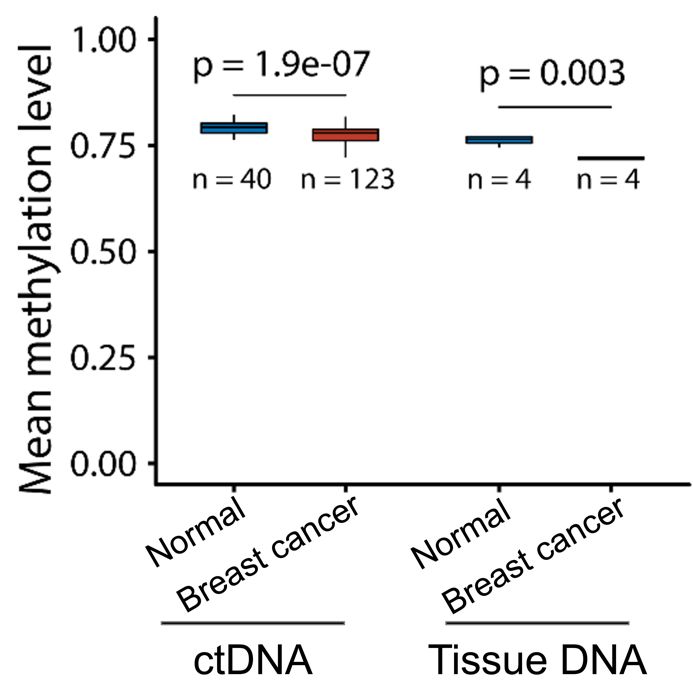
**

**FIGURE S1** Mean methylation level of ctDNA and tissue DNA was higher in normal samples than in cancer samples. p-value was computed by two-sided Student’s t-test.

**
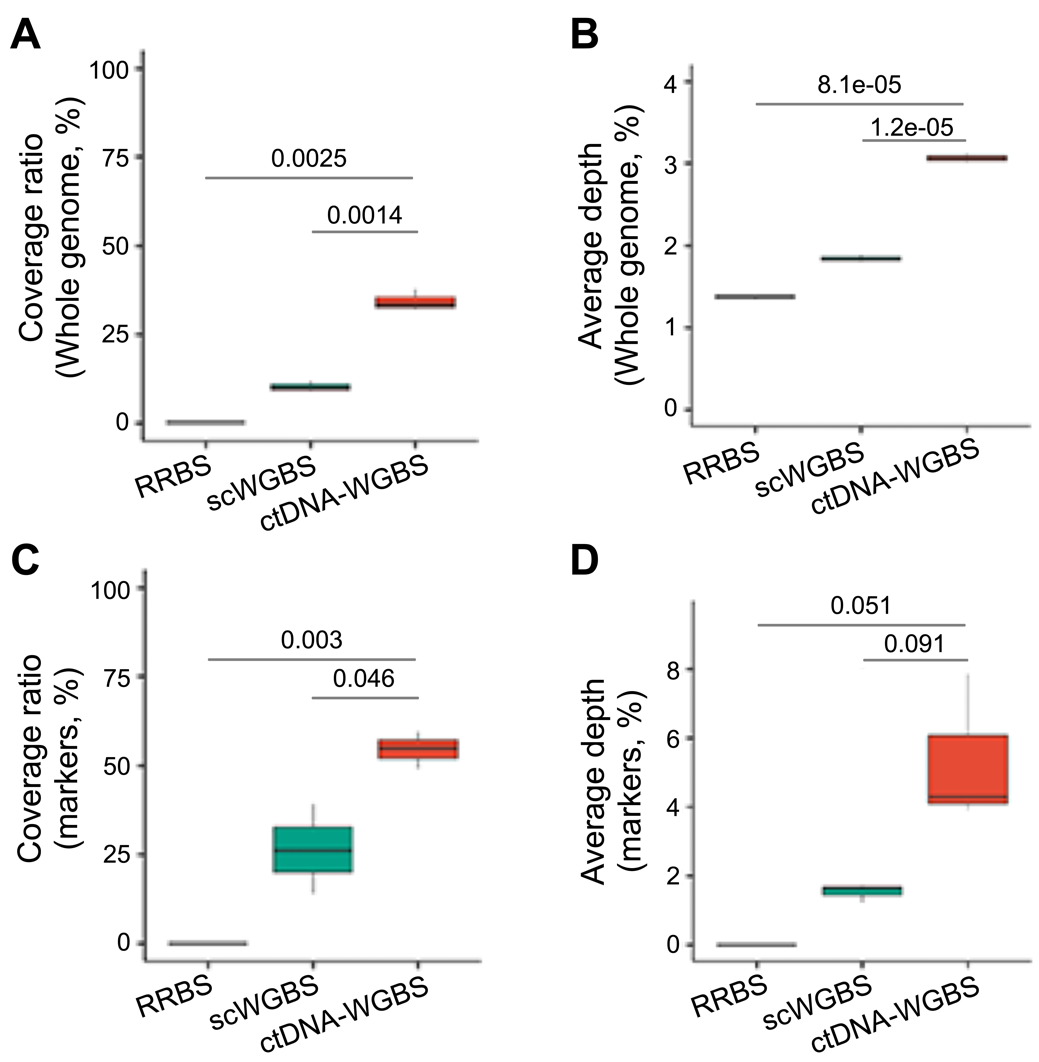
**

**FIGURE S2** Head-to-head comparison of the RRBS, scWGBS and ctDNA-WGBS sequencing results from the same sample.

**
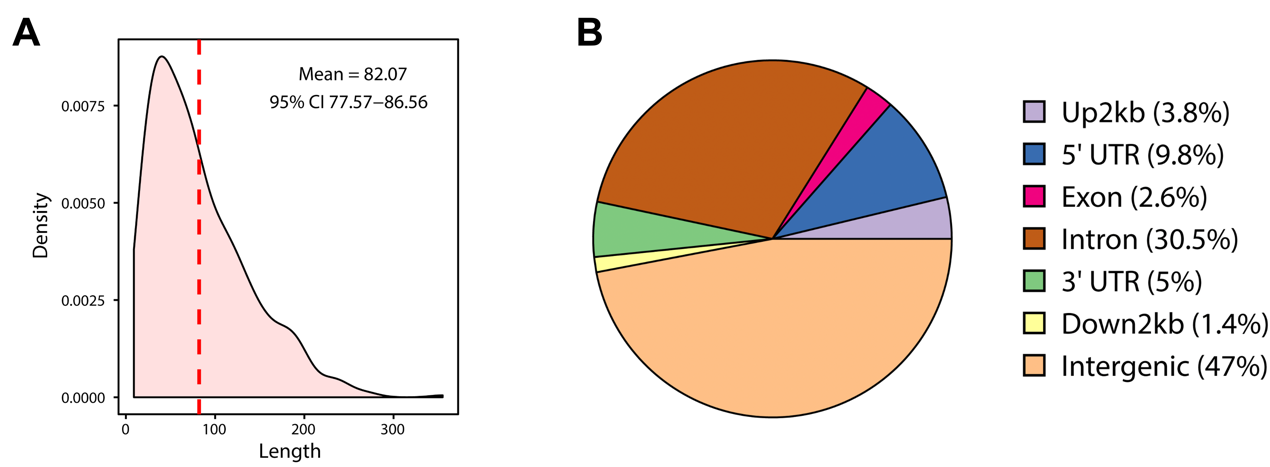
**

**FIGURE S3** Characterization of DMRs for early detection of breast cancer. Length distribution (**A**) and localization (**B**) of 583 ctDNA DMRs identified from ctDNA between normal samples and early-stage breast cancer samples.

**
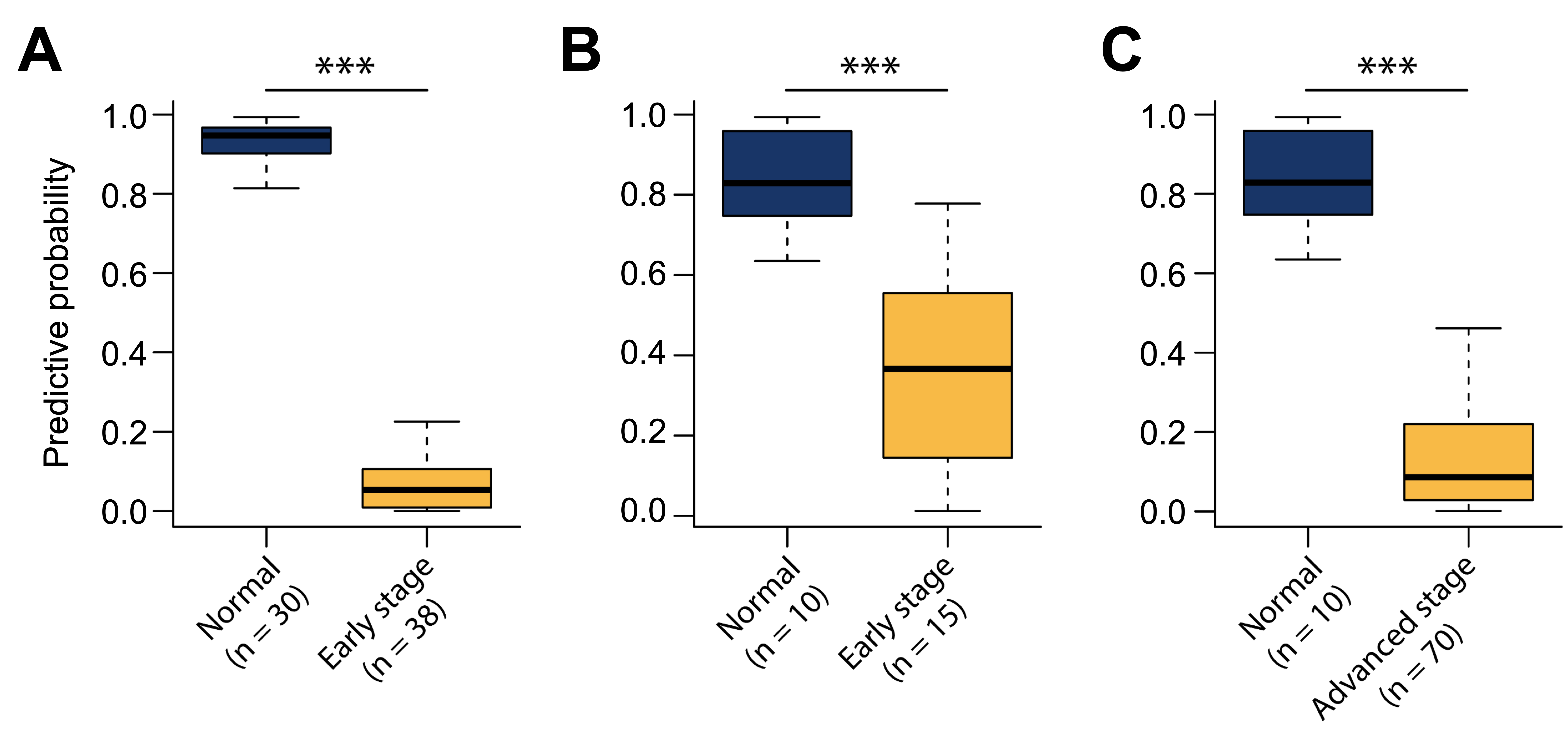
**

**FIGURE S4** Predictive probability in training set (**A**), test set 1 (**B**) and test set 2 (**C**).

**
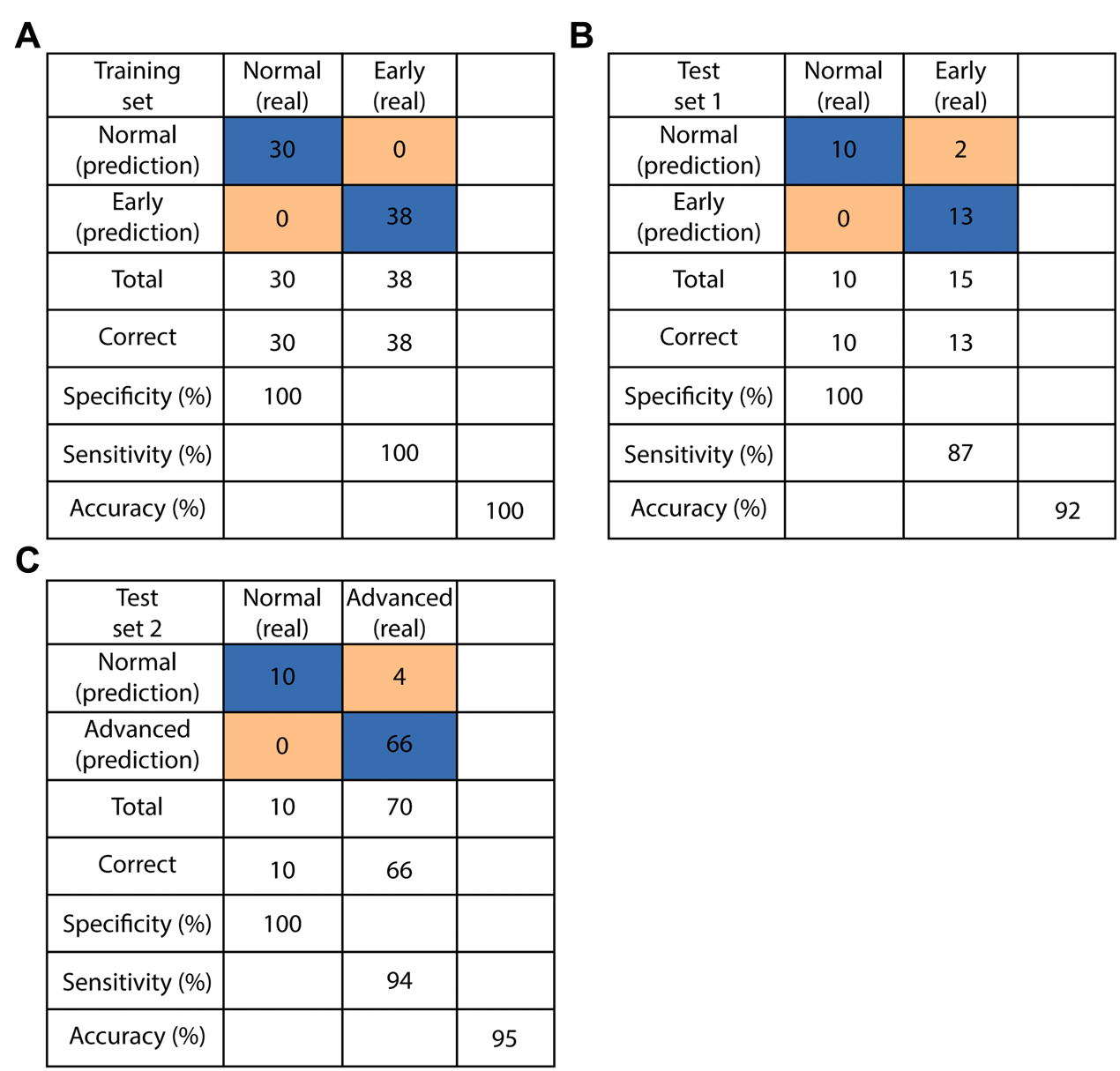
**

**FIGURE S5** Confusion matrix of early detection model in training set (**A**), test set 1 (**B**) and test set 2 (**C**).

**
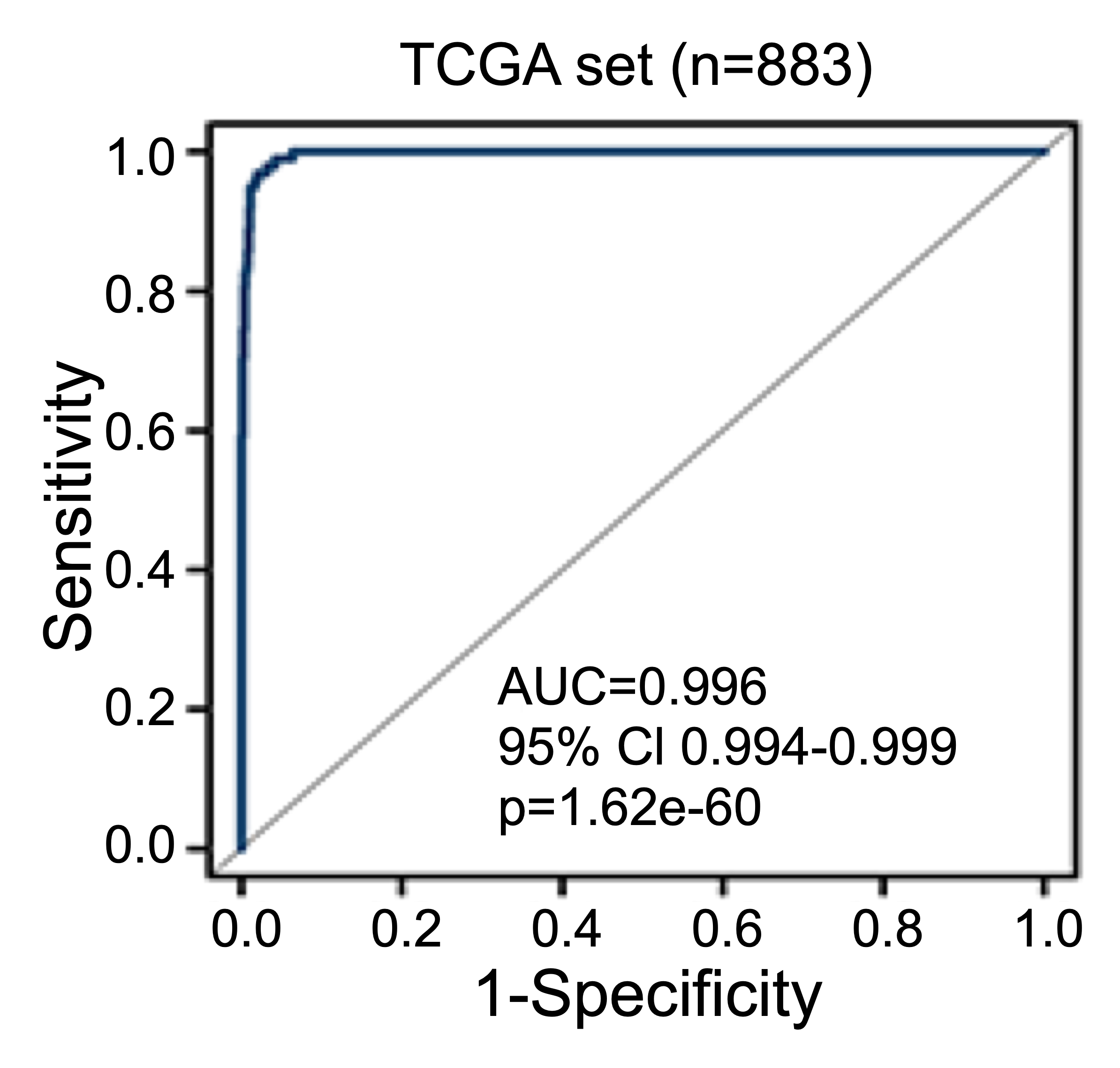
**

**FIGURE S6** The ROCs suggest the classification of healthy individuals and early breast cancer patients in the independent training and testing dataset in TCGA data.

**
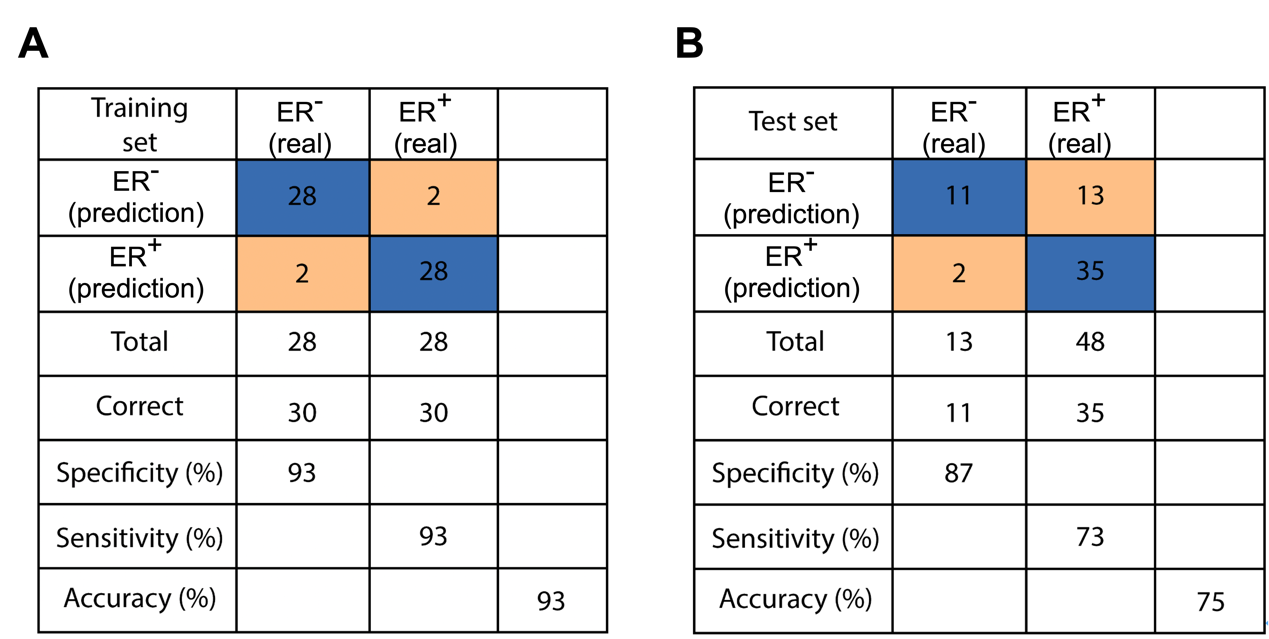
**

**FIGURE S7** Confusion matrix of ER prediction model in training set (**A**) and test set (**B**).

**
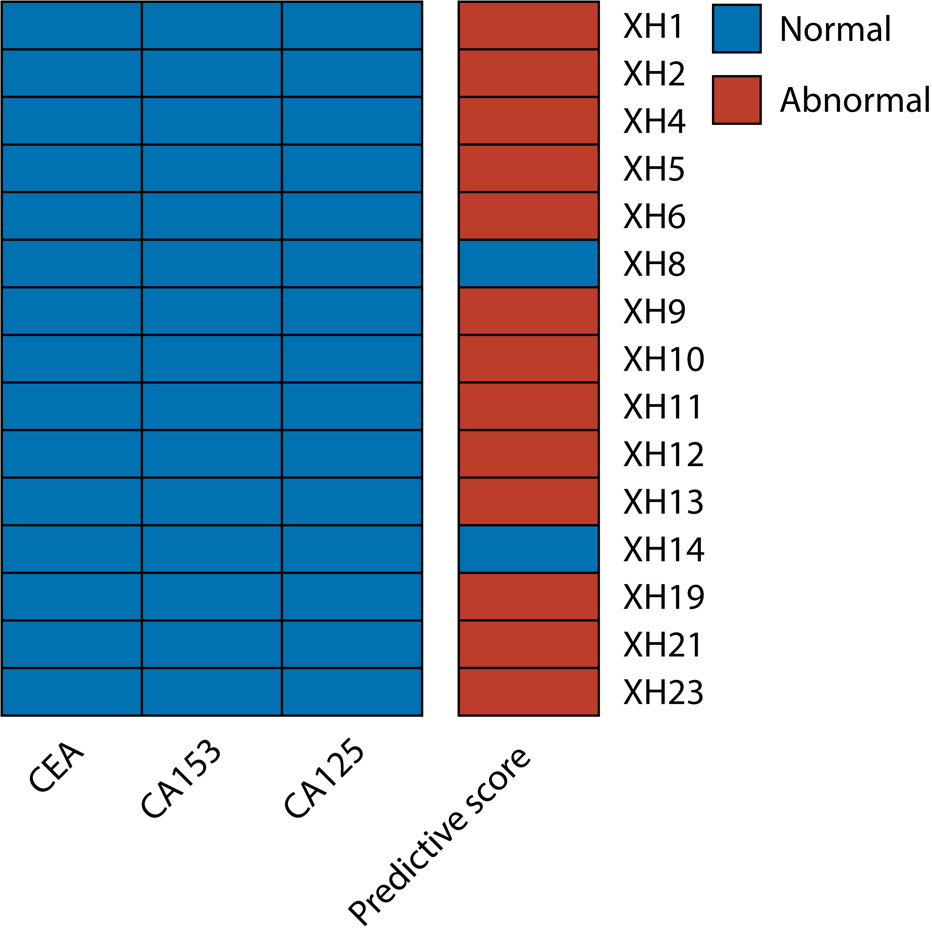
**

**FIGURE S8** Prediction result comparison of the early breast cancer patients in test set 1 (Cohort 2) by known serum protein (CEA, CA153 and CA125) with that by our framework (blue: predicted normal people; red: predicted cancer patients).

**
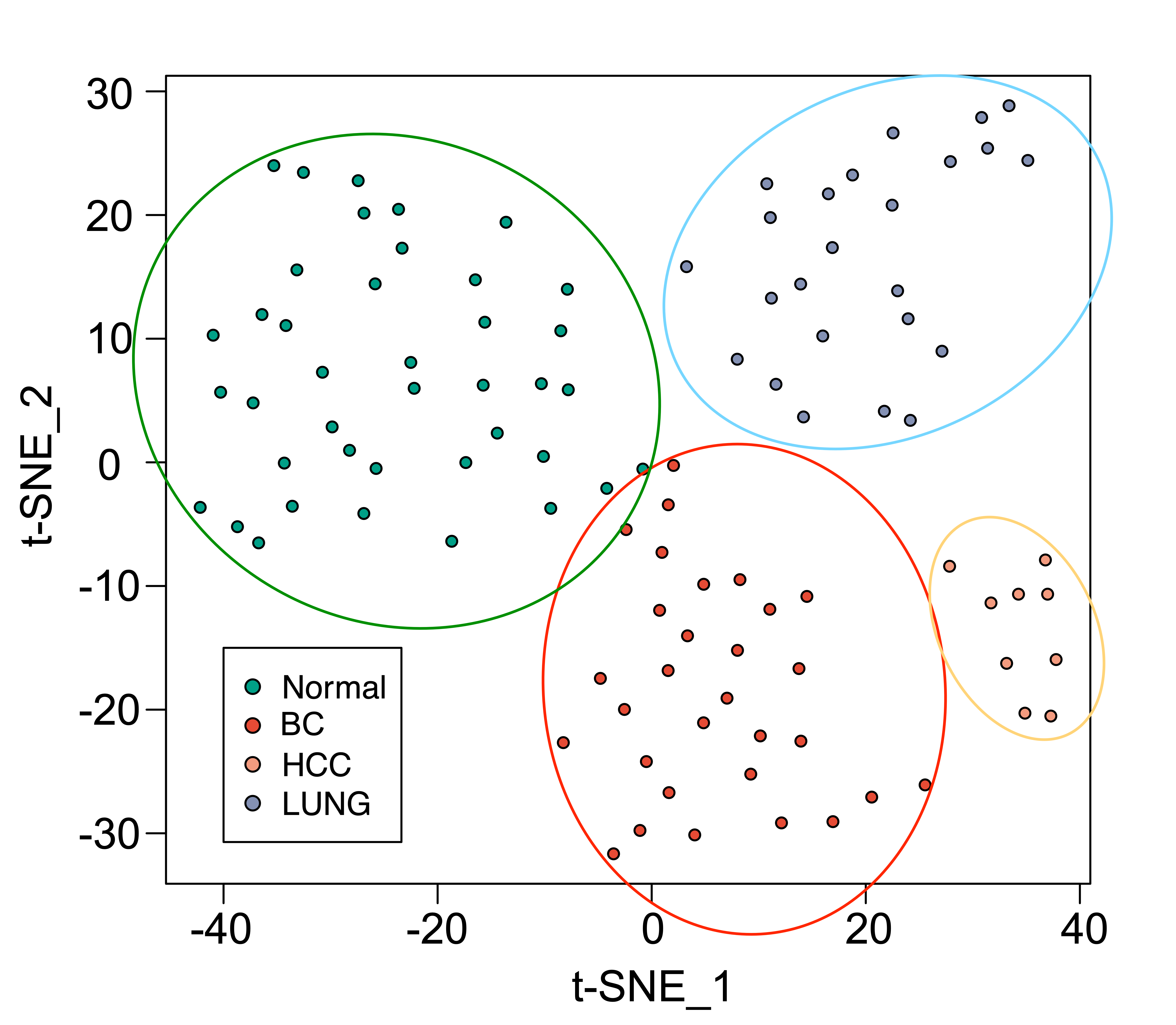
**

**FIGURE S9** t-SNE plot suggested the classification of normal controls, breast cancer (BC), hepatocellular carcinoma (HCC), and lung cancer (LUNG) patients.

**TABLE S1 ctDNA concentration of each sample.**

| **Serial ID** | **Sample** | **ctDNA (ng/mL plasma)** | **Stage** |
| --- | --- | --- | --- |
|  | N0032 | 5.82 | Normal |
|  | N0033 | 6.51 | Normal |
|  | N0034 | 3.97 | Normal |
|  | N0035 | 3.41 | Normal |
|  | N0036 | 2.58 | Normal |
|  | N0037 | 5.81 | Normal |
|  | N0038 | 8.28 | Normal |
|  | N0039 | 4.22 | Normal |
|  | N0040 | 2.03 | Normal |
|  | N0041 | 3.60 | Normal |
|  | N0042 | 2.53 | Normal |
|  | N0003 | 6.78 | Normal |
|  | N0004 | 5.72 | Normal |
|  | N0005 | 6.57 | Normal |
|  | N0006 | 5.73 | Normal |
|  | N0007 | 11.65 | Normal |
|  | N0008 | 6.44 | Normal |
|  | N0009 | 6.63 | Normal |
|  | N0010 | 12.91 | Normal |
|  | N0011 | 4.86 | Normal |
|  | N0012 | 4.63 | Normal |
|  | N0013 | 8.20 | Normal |
|  | N0014 | 4.09 | Normal |
|  | N0015 | 9.92 | Normal |
|  | N0016 | 5.69 | Normal |
|  | N0017 | 6.53 | Normal |
|  | N0018 | 7.68 | Normal |
|  | N0021 | 4.40 | Normal |
|  | N0022 | 4.87 | Normal |
|  | N0023 | 5.72 | Normal |
|  | N0024 | 6.80 | Normal |
|  | N0025 | 10.06 | Normal |
|  | N0026 | 2.28 | Normal |
|  | N0027 | 7.18 | Normal |
|  | N0028 | 2.99 | Normal |
|  | N0029 | 3.67 | Normal |
|  | N0031 | 7.05 | Normal |
|  | N0043 | 8.82 | Normal |
|  | N0044 | 8.27 | Normal |
|  | N0045 | 6.98 | Normal |
|  | TZ103 | 6.66 | Early |
|  | TZ106 | 7.93 | Early |
|  | TZ108 | 3.63 | Early |
|  | TZ109 | 11.45 | Early |
|  | TZ113 | 82.50 | Early |
|  | TZ116 | 15.12 | Early |
|  | TZ121 | 14.93 | Early |
|  | TZ127 | 10.30 | Early |
|  | TZ128 | 5.65 | Early |
|  | TZ129 | 12.55 | Early |
|  | TZ13 | 7.14 | Early |
|  | TZ130 | 8.07 | Early |
|  | TZ136 | 3.36 | Early |
|  | TZ137 | 10.56 | Early |
|  | TZ142 | 37.00 | Early |
|  | TZ144 | 6.10 | Early |
|  | TZ148 | 7.77 | Early |
|  | TZ151 | 6.91 | Early |
|  | TZ16 | 11.64 | Early |
|  | TZ18 | 7.80 | Early |
|  | TZ21 | 4.83 | Early |
|  | TZ22 | 5.40 | Early |
|  | TZ28 | 40.42 | Early |
|  | TZ41 | 9.22 | Early |
|  | TZ52 | 21.84 | Early |
|  | TZ53 | 9.66 | Early |
|  | TZ54 | 9.24 | Early |
|  | TZ61 | 20.70 | Early |
|  | TZ62 | 11.97 | Early |
|  | TZ66 | 1.23 | Early |
|  | TZ67 | 0.81 | Early |
|  | TZ68 | 0.30 | Early |
|  | TZ71 | 16.04 | Early |
|  | TZ78 | 5.37 | Early |
|  | TZ80 | 4.90 | Early |
|  | TZ84 | 6.88 | Early |
|  | TZ86 | 8.31 | Early |
|  | TZ91 | 12.65 | Early |
|  | XH1 | 5.37 | Early |
|  | XH10 | 3.77 | Early |
|  | XH11 | 4.65 | Early |
|  | XH12 | 3.02 | Early |
|  | XH13 | 9.00 | Early |
|  | XH14 | 5.30 | Early |
|  | XH19 | 9.26 | Early |
|  | XH2 | 8.94 | Early |
|  | XH21 | 6.67 | Early |
|  | XH23 | 7.51 | Early |
|  | XH4 | 15.81 | Early |
|  | XH5 | 9.69 | Early |
|  | XH6 | 8.07 | Early |
|  | XH8 | 10.20 | Early |
|  | XH9 | 5.48 | Early |
|  | TZ10 | 8.36 | Advanced |
|  | TZ105 | 12.65 | Advanced |
|  | TZ107 | 47.86 | Advanced |
|  | TZ11 | 5.74 | Advanced |
|  | TZ110 | 11.90 | Advanced |
|  | TZ112 | 17.16 | Advanced |
|  | TZ114 | 37.76 | Advanced |
|  | TZ12 | 6.02 | Advanced |
|  | TZ120 | 9.36 | Advanced |
|  | TZ125 | 14.00 | Advanced |
|  | TZ131 | 11.46 | Advanced |
|  | TZ132 | 27.72 | Advanced |
|  | TZ134 | 10.18 | Advanced |
|  | TZ135 | 14.84 | Advanced |
|  | TZ143 | 48.69 | Advanced |
|  | TZ145 | 13.73 | Advanced |
|  | TZ146 | 6.81 | Advanced |
|  | TZ15 | 10.33 | Advanced |
|  | TZ152 | 12.75 | Advanced |
|  | TZ153 | 4.40 | Advanced |
|  | TZ154 | 14.64 | Advanced |
|  | TZ155 | 36.84 | Advanced |
|  | TZ158 | 42.12 | Advanced |
|  | TZ160 | 22.36 | Advanced |
|  | TZ17 | 8.46 | Advanced |
|  | TZ19 | 3.89 | Advanced |
|  | TZ20 | 35.85 | Advanced |
|  | TZ24 | 304.69 | Advanced |
|  | TZ25 | 1071.43 | Advanced |
|  | TZ27 | 16.20 | Advanced |
|  | TZ29 | 14.00 | Advanced |
|  | TZ3 | 19.08 | Advanced |
|  | TZ30 | 15.54 | Advanced |
|  | TZ31 | 6.00 | Advanced |
|  | TZ32 | 152.83 | Advanced |
|  | TZ33 | 10.45 | Advanced |
|  | TZ34 | 16.12 | Advanced |
|  | TZ36 | 8.69 | Advanced |
|  | TZ37 | 10.77 | Advanced |
|  | TZ38 | 9.64 | Advanced |
|  | TZ39 | 114.00 | Advanced |
|  | TZ40 | 22.26 | Advanced |
|  | TZ42 | 10.26 | Advanced |
|  | TZ43 | 12.86 | Advanced |
|  | TZ45 | 12.02 | Advanced |
|  | TZ46 | 21.00 | Advanced |
|  | TZ48 | 15.84 | Advanced |
|  | TZ49 | 10.98 | Advanced |
|  | TZ5 | 12.39 | Advanced |
|  | TZ50 | 15.12 | Advanced |
|  | TZ51 | 20.52 | Advanced |
|  | TZ57 | 18.86 | Advanced |
|  | TZ58 | 23.02 | Advanced |
|  | TZ59 | 17.05 | Advanced |
|  | TZ63 | 2.66 | Advanced |
|  | TZ64 | 24.42 | Advanced |
|  | TZ65 | 79.20 | Advanced |
|  | TZ69 | 63.16 | Advanced |
|  | TZ7 | 14.43 | Advanced |
|  | TZ75 | 16.30 | Advanced |
|  | TZ76 | 12.65 | Advanced |
|  | TZ8 | 29.04 | Advanced |
|  | TZ85 | 9.30 | Advanced |
|  | TZ88 | 51.49 | Advanced |
|  | TZ9 | 12.15 | Advanced |
|  | TZ92 | 14.40 | Advanced |
|  | TZ94 | 5.75 | Advanced |
|  | TZ95 | 8.76 | Advanced |
|  | TZ97 | 11.42 | Advanced |
|  | TZ98 | 7.11 | Advanced |

**TABLE S2 Quality statistics of ctDNA and tissue WGBS for each sample.**

| **Serial ID** | **Sample ID** | **Sample origin** | **Stage** | **Raw reads** | **Clean reads** | **Mapped reads** | **Unique read** | **Mean coverage** | **Conversion ratio** |
| --- | --- | --- | --- | --- | --- | --- | --- | --- | --- |
|  | N0003 | Plasma | Normal | 259105682 | 258833414 | 172983968 | 133620800 | 3.2 | 99.23% |
|  | N0004 | Plasma | Normal | 281587058 | 281283040 | 166602928 | 124264369 | 3.0 | 99.46% |
|  | N0005 | Plasma | Normal | 272967924 | 272079400 | 173857586 | 133290237 | 3.1 | 99.34% |
|  | N0006 | Plasma | Normal | 247819294 | 247515784 | 131350426 | 101085490 | 2.9 | 99.28% |
|  | N0007 | Plasma | Normal | 250079818 | 249856570 | 149365256 | 119663193 | 3.1 | 98.59% |
|  | N0008 | Plasma | Normal | 241562482 | 241190706 | 160907682 | 128728647 | 3.2 | 99.29% |
|  | N0009 | Plasma | Normal | 269282620 | 269000426 | 128011940 | 101223337 | 2.9 | 99.07% |
|  | N0010 | Plasma | Normal | 283897202 | 283633484 | 172573824 | 136923621 | 3.2 | 98.49% |
|  | N0011 | Plasma | Normal | 248946328 | 248049562 | 155400612 | 117004774 | 2.6 | 99.78% |
|  | N0012 | Plasma | Normal | 290077102 | 289506808 | 172377474 | 128330110 | 2.5 | 99.80% |
|  | N0013 | Plasma | Normal | 275508994 | 275244808 | 165720044 | 130591263 | 3.2 | 99.36% |
|  | N0014 | Plasma | Normal | 259033006 | 258347616 | 164734362 | 105789080 | 2.2 | 99.09% |
|  | N0015 | Plasma | Normal | 236110780 | 235759866 | 114557834 | 89722096 | 2.8 | 98.05% |
|  | N0016 | Plasma | Normal | 296537872 | 296173130 | 186056450 | 143138831 | 3.5 | 99.28% |
|  | N0017 | Plasma | Normal | 258572136 | 258290840 | 156658802 | 125536772 | 3.1 | 99.16% |
|  | N0018 | Plasma | Normal | 263480982 | 263210442 | 182180102 | 141462939 | 3.4 | 99.29% |
|  | N0021 | Plasma | Normal | 259861718 | 259321548 | 170809780 | 131334112 | 2.4 | 99.83% |
|  | N0022 | Plasma | Normal | 251562998 | 251057088 | 167565258 | 126762595 | 2.4 | 99.85% |
|  | N0023 | Plasma | Normal | 296392598 | 296112204 | 127929376 | 99054406 | 2.9 | 98.56% |
|  | N0024 | Plasma | Normal | 297254390 | 296885162 | 154930318 | 122825514 | 3.0 | 98.62% |
|  | N0025 | Plasma | Normal | 301372946 | 301022998 | 134069874 | 107891515 | 3.0 | 98.78% |
|  | N0026 | Plasma | Normal | 266434024 | 265552444 | 148295960 | 91360227 | 2.2 | 99.09% |
|  | N0027 | Plasma | Normal | 261312962 | 260895874 | 99336998 | 75545577 | 2.2 | 98.79% |
|  | N0028 | Plasma | Normal | 272906552 | 271882624 | 151104468 | 84573740 | 2.1 | 99.06% |
|  | N0029 | Plasma | Normal | 268873576 | 268134382 | 134071728 | 86550404 | 2.0 | 99.11% |
|  | N0031 | Plasma | Normal | 252268222 | 251839372 | 116369770 | 82794430 | 2.2 | 98.91% |
|  | N0035 | Plasma | Normal | 310889376 | 310255506 | 187371914 | 65690925 | 2.0 | 99.08% |
|  | N0036 | Plasma | Normal | 325604260 | 324670344 | 217487294 | 30964965 | 1.5 | 98.44% |
|  | N0037 | Plasma | Normal | 294210928 | 292554188 | 203482254 | 108416727 | 2.3 | 98.64% |
|  | N0038 | Plasma | Normal | 298310202 | 294833364 | 138362104 | 39764750 | 1.7 | 98.52% |
|  | N0039 | Plasma | Normal | 290334282 | 289516998 | 190878574 | 94678009 | 2.4 | 99.00% |
|  | N0040 | Plasma | Normal | 276190014 | 275765414 | 194281898 | 136741676 | 2.8 | 98.73% |
|  | N0041 | Plasma | Normal | 310834310 | 309822718 | 189859994 | 97042472 | 2.2 | 99.05% |
|  | N0042 | Plasma | Normal | 290570370 | 289313450 | 182718364 | 86531856 | 2.1 | 99.12% |
|  | N0032 | Plasma | Normal | 316841962 | 315996474 | 167530270 | 122364366 | 2.7 | 98.63% |
|  | N0033 | Plasma | Normal | 356899292 | 356206812 | 222913560 | 151717889 | 3.3 | 98.92% |
|  | N0034 | Plasma | Normal | 267194202 | 266434424 | 141551780 | 98434344 | 2.5 | 98.99% |
|  | N0043 | Plasma | Normal | 279298478 | 278580582 | 184504422 | 126661860 | 2.5 | 98.82% |
|  | N0044 | Plasma | Normal | 293209126 | 292915456 | 167291052 | 119365435 | 3.1 | 98.76% |
|  | N0045 | Plasma | Normal | 298952064 | 298526694 | 191673268 | 134648010 | 2.7 | 99.16% |
|  | TZ10 | Plasma | Advanced | 290373040 | 289773652 | 198371762 | 161418373 | 2.8 | 98.86% |
|  | TZ103 | Plasma | Early | 270788204 | 270029174 | 161961508 | 66126479 | 1.8 | 99.22% |
|  | TZ105 | Plasma | Advanced | 313349418 | 312913404 | 195330452 | 122864307 | 2.9 | 98.76% |
|  | TZ106 | Plasma | Early | 328168886 | 327751166 | 190222098 | 132186427 | 3.1 | 98.76% |
|  | TZ107 | Plasma | Advanced | 307133626 | 306763744 | 192616684 | 134678095 | 3.0 | 98.78% |
|  | TZ108 | Plasma | Early | 358782596 | 358050480 | 201409944 | 94216310 | 2.2 | 99.32% |
|  | TZ109 | Plasma | Early | 252610110 | 251434002 | 135362468 | 80885757 | 2.2 | 98.71% |
|  | TZ11 | Plasma | Advanced | 335757182 | 334840356 | 165469550 | 33340688 | 1.4 | 98.88% |
|  | TZ110 | Plasma | Advanced | 214327730 | 213863696 | 120816474 | 60997672 | 2.0 | 99.40% |
|  | TZ112 | Plasma | Advanced | 201408994 | 200682958 | 120721040 | 72426674 | 1.8 | 99.02% |
|  | TZ113 | Plasma | Early | 175534912 | 175025016 | 101593310 | 70143751 | 1.8 | 98.72% |
|  | TZ114 | Plasma | Advanced | 229863218 | 229017300 | 130744292 | 79233851 | 2.0 | 98.80% |
|  | TZ116 | Plasma | Early | 289386244 | 288636096 | 169953588 | 109735264 | 2.2 | 99.03% |
|  | TZ12 | Plasma | Advanced | 316917036 | 315898922 | 189448638 | 77232682 | 1.9 | 98.80% |
|  | TZ120 | Plasma | Advanced | 246376918 | 245646482 | 161152304 | 86823226 | 2.0 | 99.11% |
|  | TZ121 | Plasma | Early | 236533656 | 235997970 | 158304606 | 114127489 | 2.2 | 99.08% |
|  | TZ125 | Plasma | Advanced | 450640738 | 449365996 | 285808740 | 186385009 | 3.5 | 98.93% |
|  | TZ127 | Plasma | Early | 376808648 | 375759816 | 233382570 | 127323642 | 2.7 | 99.21% |
|  | TZ128 | Plasma | Early | 165881304 | 165644166 | 112927254 | 83289003 | 2.3 | 98.97% |
|  | TZ129 | Plasma | Early | 253067576 | 252428758 | 159560640 | 113551421 | 2.7 | 98.45% |
|  | TZ13 | Plasma | Early | 348170282 | 347028234 | 200413082 | 77676790 | 2.2 | 98.78% |
|  | TZ130 | Plasma | Early | 247824414 | 247049342 | 156149992 | 81608580 | 2.2 | 99.28% |
|  | TZ131 | Plasma | Advanced | 144700282 | 144277272 | 74355834 | 50818739 | 1.7 | 98.78% |
|  | TZ132 | Plasma | Advanced | 309383394 | 308893510 | 200815414 | 141481026 | 2.5 | 99.08% |
|  | TZ134 | Plasma | Advanced | 240837130 | 240290478 | 133467728 | 100958925 | 2.2 | 98.27% |
|  | TZ135 | Plasma | Advanced | 236427762 | 235985100 | 143127052 | 108851342 | 2.5 | 98.83% |
|  | TZ136 | Plasma | Early | 260890886 | 260579800 | 174389080 | 127497376 | 2.7 | 99.24% |
|  | TZ137 | Plasma | Early | 271047590 | 270287606 | 156144170 | 94080618 | 2.2 | 98.28% |
|  | TZ142 | Plasma | Early | 282354280 | 281826092 | 159208324 | 110202875 | 2.3 | 99.19% |
|  | TZ143 | Plasma | Advanced | 256918012 | 256400194 | 141825218 | 111245499 | 2.4 | 98.74% |
|  | TZ144 | Plasma | Early | 302225890 | 301563816 | 136226168 | 95654494 | 2.5 | 98.54% |
|  | TZ145 | Plasma | Advanced | 217354914 | 216741790 | 140251758 | 107416798 | 2.7 | 98.89% |
|  | TZ146 | Plasma | Advanced | 283840224 | 283377214 | 154976472 | 117197628 | 2.8 | 99.15% |
|  | TZ148 | Plasma | Early | 259585426 | 259106774 | 157100904 | 118189244 | 2.6 | 99.18% |
|  | TZ15 | Plasma | Advanced | 313203638 | 312775156 | 208937776 | 166373773 | 3.1 | 98.77% |
|  | TZ151 | Plasma | Early | 240967210 | 240661016 | 180921600 | 145929742 | 3.1 | 98.65% |
|  | TZ152 | Plasma | Advanced | 215671070 | 215090358 | 136994314 | 98691082 | 2.8 | 99.21% |
|  | TZ153 | Plasma | Advanced | 239946190 | 239083426 | 146515364 | 90699660 | 2.6 | 98.76% |
|  | TZ154 | Plasma | Advanced | 264506380 | 263800538 | 163859056 | 124820561 | 2.6 | 97.85% |
|  | TZ155 | Plasma | Advanced | 250400460 | 249773042 | 172458362 | 125250310 | 2.6 | 99.36% |
|  | TZ158 | Plasma | Advanced | 230677002 | 230099392 | 148044774 | 110263607 | 2.7 | 99.18% |
|  | TZ16 | Plasma | Early | 302294526 | 301833772 | 201087166 | 156951318 | 3.0 | 98.84% |
|  | TZ160 | Plasma | Advanced | 255392066 | 254748392 | 162182666 | 111146627 | 2.2 | 99.28% |
|  | TZ17 | Plasma | Advanced | 295565184 | 294826902 | 158462040 | 122718664 | 2.8 | 98.84% |
|  | TZ18 | Plasma | Early | 293493652 | 292231258 | 172384866 | 83763088 | 2.3 | 98.82% |
|  | TZ19 | Plasma | Advanced | 293974978 | 293509552 | 171642242 | 64674495 | 1.8 | 98.68% |
|  | TZ20 | Plasma | Advanced | 327247618 | 326606518 | 168932806 | 142549062 | 3.0 | 99.23% |
|  | TZ21 | Plasma | Early | 317776510 | 316742418 | 163987440 | 104103585 | 2.5 | 98.80% |
|  | TZ22 | Plasma | Early | 362663042 | 361778462 | 186286644 | 97701424 | 2.6 | 98.36% |
|  | TZ24 | Plasma | Advanced | 346883084 | 346194600 | 202152126 | 165879491 | 2.8 | 98.77% |
|  | TZ25 | Plasma | Advanced | 378794136 | 377864750 | 207773166 | 168866426 | 3.2 | 98.96% |
|  | TZ27 | Plasma | Advanced | 306518580 | 305972708 | 166948032 | 141948754 | 2.9 | 98.99% |
|  | TZ28 | Plasma | Early | 359698164 | 358581568 | 200887264 | 167079797 | 2.9 | 98.97% |
|  | TZ29 | Plasma | Advanced | 299527898 | 299058962 | 201367024 | 170401421 | 3.2 | 99.03% |
|  | TZ3 | Plasma | Advanced | 186960106 | 186489596 | 116367892 | 93012653 | 2.5 | 98.81% |
|  | TZ30 | Plasma | Advanced | 323022978 | 322648878 | 198386730 | 136929440 | 2.8 | 98.82% |
|  | TZ31 | Plasma | Advanced | 280588476 | 279393140 | 129109594 | 98654908 | 2.3 | 98.19% |
|  | TZ32 | Plasma | Advanced | 270654222 | 269766378 | 136616976 | 94268866 | 2.4 | 98.71% |
|  | TZ33 | Plasma | Advanced | 333817184 | 333103588 | 184682034 | 157060985 | 3.2 | 99.11% |
|  | TZ34 | Plasma | Advanced | 363911234 | 362811502 | 201829412 | 156591004 | 3.3 | 99.04% |
|  | TZ36 | Plasma | Advanced | 356932928 | 356567016 | 71222900 | 57359122 | 2.0 | 98.77% |
|  | TZ37 | Plasma | Advanced | 327979596 | 327450796 | 164340424 | 128994375 | 2.5 | 98.85% |
|  | TZ38 | Plasma | Advanced | 331016526 | 330272744 | 205423996 | 168719123 | 3.7 | 98.93% |
|  | TZ39 | Plasma | Advanced | 297261366 | 296585262 | 182464372 | 155586599 | 3.3 | 98.91% |
|  | TZ40 | Plasma | Advanced | 288460430 | 287859130 | 181451628 | 143249450 | 3.0 | 98.72% |
|  | TZ41 | Plasma | Early | 295083522 | 294573208 | 174533478 | 141167957 | 2.8 | 98.81% |
|  | TZ42 | Plasma | Advanced | 259717650 | 258187310 | 174880912 | 112899262 | 2.3 | 98.83% |
|  | TZ43 | Plasma | Advanced | 309385988 | 308608480 | 202771514 | 110791122 | 2.3 | 98.71% |
|  | TZ45 | Plasma | Advanced | 291802004 | 291263820 | 156859844 | 84651761 | 2.2 | 99.00% |
|  | TZ46 | Plasma | Advanced | 305276626 | 304330698 | 207858186 | 156576381 | 3.2 | 98.73% |
|  | TZ48 | Plasma | Advanced | 265164742 | 264385224 | 148046286 | 112244235 | 2.3 | 98.68% |
|  | TZ49 | Plasma | Advanced | 339080162 | 337164050 | 143327246 | 79616022 | 2.0 | 98.68% |
|  | TZ5 | Plasma | Advanced | 160560240 | 160231230 | 112117930 | 94955326 | 2.5 | 98.03% |
|  | TZ50 | Plasma | Advanced | 309290036 | 307965256 | 177379448 | 101663635 | 2.2 | 98.85% |
|  | TZ51 | Plasma | Advanced | 231028280 | 230066762 | 98980858 | 70301906 | 1.9 | 98.25% |
|  | TZ52 | Plasma | Early | 252463064 | 251736146 | 140213724 | 101641630 | 2.1 | 98.78% |
|  | TZ53 | Plasma | Early | 323969996 | 323200568 | 190977638 | 121812718 | 2.4 | 98.44% |
|  | TZ54 | Plasma | Early | 294945454 | 294165400 | 179248416 | 112934614 | 2.4 | 98.68% |
|  | TZ57 | Plasma | Advanced | 273739682 | 272438638 | 142493154 | 92743004 | 2.1 | 98.48% |
|  | TZ58 | Plasma | Advanced | 299461764 | 298767436 | 154363764 | 74536033 | 2.1 | 98.59% |
|  | TZ59 | Plasma | Advanced | 335633676 | 334009338 | 201469508 | 108476804 | 2.5 | 98.39% |
|  | TZ61 | Plasma | Early | 323733612 | 322870768 | 181869686 | 112011830 | 2.8 | 98.97% |
|  | TZ62 | Plasma | Early | 275507088 | 274711208 | 156506154 | 97749915 | 2.5 | 98.96% |
|  | TZ63 | Plasma | Advanced | 343469184 | 342616230 | 191900354 | 32354797 | 1.6 | 98.89% |
|  | TZ64 | Plasma | Advanced | 358200618 | 357277696 | 186726870 | 124367255 | 2.7 | 98.97% |
|  | TZ65 | Plasma | Advanced | 290256426 | 289786184 | 160153700 | 66062363 | 1.8 | 98.59% |
|  | TZ66 | Plasma | Early | 309682838 | 308730464 | 149451876 | 92842915 | 2.4 | 98.76% |
|  | TZ67 | Plasma | Early | 367810128 | 366974726 | 173735372 | 61608196 | 2.1 | 98.56% |
|  | TZ68 | Plasma | Early | 332528702 | 331594822 | 167259828 | 76847383 | 2.0 | 98.76% |
|  | TZ69 | Plasma | Advanced | 302516146 | 301565084 | 137420258 | 98559760 | 2.4 | 98.87% |
|  | TZ7 | Plasma | Advanced | 158377168 | 157985302 | 107013420 | 88437811 | 2.4 | 98.10% |
|  | TZ71 | Plasma | Early | 254116446 | 253430552 | 148092712 | 94582141 | 2.2 | 99.34% |
|  | TZ75 | Plasma | Advanced | 321291680 | 320443742 | 200593446 | 140275947 | 3.0 | 98.85% |
|  | T76 | Plasma | Advanced | 336888540 | 336330332 | 170395668 | 119932320 | 2.7 | 98.88% |
|  | TZ78 | Plasma | Early | 197799214 | 197507412 | 120949978 | 77992230 | 1.9 | 98.83% |
|  | TZ8 | Plasma | Advanced | 161423852 | 161137700 | 87378758 | 73673846 | 2.0 | 98.74% |
|  | TZ80 | Plasma | Early | 261256602 | 260789216 | 147621404 | 112288088 | 2.7 | 98.24% |
|  | TZ84 | Plasma | Early | 315618034 | 314818310 | 157563528 | 94716972 | 2.2 | 98.90% |
|  | TZ85 | Plasma | Advanced | 267681460 | 267126618 | 160269744 | 114540897 | 2.7 | 99.12% |
|  | TZ86 | Plasma | Early | 238985862 | 238479930 | 153732412 | 120998542 | 2.9 | 98.77% |
|  | TZ88 | Plasma | Advanced | 236072086 | 235686872 | 166256442 | 126274959 | 2.7 | 98.83% |
|  | TZ9 | Plasma | Advanced | 342608782 | 342189634 | 218111302 | 159244562 | 3.2 | 99.03% |
|  | TZ91 | Plasma | Early | 268732296 | 267803970 | 179216362 | 122649420 | 2.3 | 98.94% |
|  | TZ92 | Plasma | Advanced | 239023834 | 238666280 | 173110136 | 105194389 | 2.6 | 99.34% |
|  | TZ94 | Plasma | Advanced | 282653744 | 281950610 | 146459558 | 107719342 | 2.8 | 98.78% |
|  | TZ95 | Plasma | Advanced | 279036702 | 278329098 | 143968528 | 105866525 | 2.6 | 98.59% |
|  | TZ97 | Plasma | Advanced | 325185082 | 324490246 | 179464294 | 123342702 | 2.9 | 98.72% |
|  | TZ98 | Plasma | Advanced | 166837102 | 166396254 | 106412440 | 52687157 | 1.7 | 99.07% |
|  | XH1 | Plasma | Early | 273356358 | 272943444 | 163766828 | 117597905 | 2.4 | 98.61% |
|  | XH10 | Plasma | Early | 305932676 | 305506156 | 169463100 | 128094011 | 3.1 | 98.88% |
|  | XH11 | Plasma | Early | 312758742 | 312366930 | 193303692 | 145526643 | 3.4 | 98.92% |
|  | XH12 | Plasma | Early | 300859656 | 300345732 | 181372996 | 139989998 | 3.1 | 99.09% |
|  | XH13 | Plasma | Early | 276232088 | 275877846 | 145999234 | 111017676 | 3.0 | 98.92% |
|  | XH14 | Plasma | Early | 284758302 | 284484572 | 189045698 | 149583536 | 3.2 | 98.97% |
|  | XH19 | Plasma | Early | 276485726 | 276257446 | 164773590 | 133183174 | 3.1 | 99.00% |
|  | XH2 | Plasma | Early | 310995988 | 310281292 | 211649976 | 135532052 | 2.6 | 98.40% |
|  | XH21 | Plasma | Early | 258178996 | 257606858 | 158661716 | 108770811 | 2.4 | 99.18% |
|  | XH23 | Plasma | Early | 263804824 | 263321392 | 158388310 | 120980892 | 2.6 | 99.26% |
|  | XH4 | Plasma | Early | 271885494 | 271511422 | 192259322 | 137438117 | 2.8 | 98.56% |
|  | XH5 | Plasma | Early | 293778756 | 293187512 | 205318870 | 146389954 | 2.7 | 98.51% |
|  | XH6 | Plasma | Early | 287124638 | 286615304 | 186565314 | 127626902 | 2.6 | 98.55% |
|  | XH8 | Plasma | Early | 248956548 | 248655064 | 178939350 | 139664623 | 2.8 | 98.79% |
|  | XH9 | Plasma | Early | 307416610 | 306703470 | 208786806 | 140181974 | 2.6 | 99.06% |
|  | FD16N | Tissue | Early | 436120526 | 435672478 | 324328782 | 251467095 | 4.6 | 99.58% |
|  | FD16T | Tissue | Early | 495973772 | 495269834 | 343355228 | 254434545 | 5.1 | 99.67% |
|  | FD17N | Tissue | Early | 463986114 | 463255724 | 316708702 | 133674399 | 3.5 | 99.68% |
|  | FD17T | Tissue | Early | 460603474 | 459881996 | 313855796 | 226847211 | 5.0 | 99.68% |
|  | FD27N | Tissue | Early | 439477782 | 438644054 | 276803960 | 199522913 | 4.7 | 99.44% |
|  | FD27T | Tissue | Early | 460182640 | 459231618 | 278682178 | 200317503 | 4.8 | 99.63% |
|  | FD4N | Tissue | Early | 451660920 | 450007330 | 289400956 | 203247414 | 4.6 | 99.23% |
|  | FD4T | Tissue | Early | 492333166 | 490588668 | 314215168 | 214411094 | 5.2 | 99.47% |

**TABLE S3 Summary of WGBS data.**

| **Samples origin** | **Stage** | **Number of samples** | **Total reads (Mean±SD)** | **Unique reads (Mean±SD)** | **Ratio of bisulfite conversion (Mean±SD)** | **Ratio of covered CpGs (Mean±SD)** |
| --- | --- | --- | --- | --- | --- | --- |
| Plasma | Normal | 40 | 279303703±25269146 | 109552284±27032036 | 99%±0.4% | 65.7%±8.9% |
| Plasma | Early Cancer | 53 | 285685137±44027069 | 112744288±25008956 | 98.8%±0.3% | 70.0%±7.6% |
| Plasma | Advanced Cancer | 70 | 283135116±58596896 | 111693294±35533040 | 98.8%±0.3% | 69.6%±8.3% |
| Tissue | Normal | 4 | 447811336±12682874 | 196977955±48381090 | 99.5%±0.2% | 91.3%±0.3% |
| Tissue | Early Cancer | 4 | 477273263±19548921 | 224002588±23001275 | 99.6%±0.1% | 89.8%±2.8% |

**TABLE S4 Chromosome location of 583 differentially methylated regions in early detection of breast cancer.**

| **Serial ID** | **Chromosome** | **Start** | **End** |
| --- | --- | --- | --- |
|  | chr1 | 56297 | 56447 |
|  | chr1 | 1696760 | 1696797 |
|  | chr1 | 1812358 | 1812447 |
|  | chr1 | 5100282 | 5100373 |
|  | chr1 | 5521217 | 5521335 |
|  | chr1 | 6362891 | 6362913 |
|  | chr1 | 9288935 | 9289092 |
|  | chr1 | 10211349 | 10211472 |
|  | chr1 | 15065245 | 15065280 |
|  | chr1 | 19451036 | 19451269 |
|  | chr1 | 19764720 | 19764824 |
|  | chr1 | 21504788 | 21504921 |
|  | chr1 | 25745546 | 25745626 |
|  | chr1 | 26265875 | 26265911 |
|  | chr1 | 28634319 | 28634428 |
|  | chr1 | 30215039 | 30215102 |
|  | chr1 | 31054038 | 31054291 |
|  | chr1 | 37615093 | 37615171 |
|  | chr1 | 39456075 | 39456108 |
|  | chr1 | 46822889 | 46822966 |
|  | chr1 | 47900302 | 47900387 |
|  | chr1 | 70443676 | 70443869 |
|  | chr1 | 79057762 | 79057867 |
|  | chr1 | 87770862 | 87770899 |
|  | chr1 | 92200406 | 92200561 |
|  | chr1 | 95698614 | 95698810 |
|  | chr1 | 112684441 | 112684589 |
|  | chr1 | 115000444 | 115000533 |
|  | chr1 | 117416576 | 117416657 |
|  | chr1 | 119377931 | 119378042 |
|  | chr1 | 143437625 | 143437696 |
|  | chr1 | 153595780 | 153595859 |
|  | chr1 | 159796996 | 159797062 |
|  | chr1 | 161397898 | 161397919 |
|  | chr1 | 167031190 | 167031255 |
|  | chr1 | 180952925 | 180952941 |
|  | chr1 | 201122342 | 201122383 |
|  | chr1 | 207485153 | 207485183 |
|  | chr1 | 208415109 | 208415123 |
|  | chr1 | 209939354 | 209939395 |
|  | chr1 | 217807365 | 217807598 |
|  | chr1 | 226151687 | 226151708 |
|  | chr1 | 226901148 | 226901183 |
|  | chr1 | 228272736 | 228272813 |
|  | chr1 | 228658496 | 228658547 |
|  | chr1 | 230134825 | 230134987 |
|  | chr1 | 233427267 | 233427297 |
|  | chr1 | 233907888 | 233907907 |
|  | chr1 | 235587206 | 235587238 |
|  | chr1 | 238563302 | 238563367 |
|  | chr1 | 246888756 | 246888897 |
|  | chr1 | 248009094 | 248009164 |
|  | chr10 | 15910095 | 15910267 |
|  | chr10 | 17064964 | 17064993 |
|  | chr10 | 25347013 | 25347037 |
|  | chr10 | 26742667 | 26742844 |
|  | chr10 | 28908964 | 28908994 |
|  | chr10 | 29546509 | 29546613 |
|  | chr10 | 34540911 | 34540983 |
|  | chr10 | 37247885 | 37247948 |
|  | chr10 | 39014665 | 39014692 |
|  | chr10 | 49866280 | 49866292 |
|  | chr10 | 60976189 | 60976369 |
|  | chr10 | 62808875 | 62808907 |
|  | chr10 | 71440556 | 71440637 |
|  | chr10 | 115828267 | 115828404 |
|  | chr10 | 123208929 | 123208973 |
|  | chr10 | 126548038 | 126548093 |
|  | chr10 | 130341850 | 130342023 |
|  | chr10 | 130505738 | 130505775 |
|  | chr10 | 133990313 | 133990367 |
|  | chr10 | 134448226 | 134448253 |
|  | chr11 | 1360377 | 1360405 |
|  | chr11 | 2168362 | 2168588 |
|  | chr11 | 3177591 | 3177658 |
|  | chr11 | 3511856 | 3511880 |
|  | chr11 | 4235151 | 4235231 |
|  | chr11 | 9384822 | 9385013 |
|  | chr11 | 9772237 | 9772288 |
|  | chr11 | 15847745 | 15847821 |
|  | chr11 | 19391325 | 19391350 |
|  | chr11 | 20410055 | 20410219 |
|  | chr11 | 33255001 | 33255113 |
|  | chr11 | 33994230 | 33994248 |
|  | chr11 | 48770314 | 48770367 |
|  | chr11 | 66394467 | 66394510 |
|  | chr11 | 68794979 | 68795065 |
|  | chr11 | 69182363 | 69182397 |
|  | chr11 | 71142092 | 71142160 |
|  | chr11 | 72320715 | 72320770 |
|  | chr11 | 75831714 | 75831885 |
|  | chr11 | 91982421 | 91982524 |
|  | chr11 | 93654877 | 93654937 |
|  | chr11 | 100341376 | 100341509 |
|  | chr11 | 109992079 | 109992105 |
|  | chr11 | 114085363 | 114085431 |
|  | chr11 | 117165514 | 117165599 |
|  | chr11 | 120106405 | 120106601 |
|  | chr11 | 122579243 | 122579407 |
|  | chr11 | 122647477 | 122647634 |
|  | chr11 | 126321813 | 126322168 |
|  | chr11 | 127215594 | 127215624 |
|  | chr11 | 133569129 | 133569248 |
|  | chr12 | 2144903 | 2144980 |
|  | chr12 | 2605762 | 2606006 |
|  | chr12 | 3185732 | 3185866 |
|  | chr12 | 6427559 | 6427602 |
|  | chr12 | 6662192 | 6662257 |
|  | chr12 | 8205424 | 8205524 |
|  | chr12 | 26880771 | 26881041 |
|  | chr12 | 31219256 | 31219387 |
|  | chr12 | 31385685 | 31385767 |
|  | chr12 | 33595117 | 33595280 |
|  | chr12 | 48335308 | 48335371 |
|  | chr12 | 52219688 | 52219702 |
|  | chr12 | 57443176 | 57443207 |
|  | chr12 | 58272306 | 58272392 |
|  | chr12 | 65143310 | 65143406 |
|  | chr12 | 67278141 | 67278185 |
|  | chr12 | 77726295 | 77726393 |
|  | chr12 | 86035436 | 86035590 |
|  | chr12 | 94365005 | 94365035 |
|  | chr12 | 95353451 | 95353507 |
|  | chr12 | 95409198 | 95409254 |
|  | chr12 | 105184404 | 105184452 |
|  | chr12 | 115062831 | 115062905 |
|  | chr12 | 116980450 | 116980648 |
|  | chr12 | 117299141 | 117299199 |
|  | chr12 | 118631134 | 118631159 |
|  | chr12 | 119591006 | 119591095 |
|  | chr12 | 122760959 | 122761037 |
|  | chr12 | 124118920 | 124118985 |
|  | chr12 | 124286311 | 124286428 |
|  | chr12 | 128447401 | 128447438 |
|  | chr12 | 130423331 | 130423491 |
|  | chr12 | 133242272 | 133242328 |
|  | chr13 | 21708436 | 21708506 |
|  | chr13 | 24863145 | 24863323 |
|  | chr13 | 30982970 | 30983009 |
|  | chr13 | 33609195 | 33609371 |
|  | chr13 | 37430338 | 37430470 |
|  | chr13 | 37552091 | 37552151 |
|  | chr13 | 42438942 | 42439077 |
|  | chr13 | 48839455 | 48839497 |
|  | chr13 | 48895477 | 48895551 |
|  | chr13 | 50196835 | 50196892 |
|  | chr13 | 69197250 | 69197333 |
|  | chr13 | 74706482 | 74706556 |
|  | chr13 | 75114980 | 75115041 |
|  | chr13 | 75794923 | 75795036 |
|  | chr13 | 105661086 | 105661117 |
|  | chr13 | 107448954 | 107449001 |
|  | chr13 | 110623879 | 110623919 |
|  | chr13 | 114461427 | 114461480 |
|  | chr14 | 21820956 | 21820979 |
|  | chr14 | 29377705 | 29377763 |
|  | chr14 | 34487839 | 34487861 |
|  | chr14 | 36699123 | 36699262 |
|  | chr14 | 42725390 | 42725488 |
|  | chr14 | 51260757 | 51260807 |
|  | chr14 | 57034146 | 57034220 |
|  | chr14 | 57359876 | 57359920 |
|  | chr14 | 58605919 | 58606098 |
|  | chr14 | 64911046 | 64911120 |
|  | chr14 | 65068581 | 65068650 |
|  | chr14 | 72944360 | 72944400 |
|  | chr14 | 77383072 | 77383093 |
|  | chr14 | 77709261 | 77709338 |
|  | chr14 | 80264214 | 80264403 |
|  | chr14 | 88500907 | 88500948 |
|  | chr14 | 92412705 | 92412741 |
|  | chr14 | 95621582 | 95621737 |
|  | chr14 | 97880199 | 97880296 |
|  | chr14 | 98530738 | 98530777 |
|  | chr14 | 98846821 | 98846991 |
|  | chr14 | 99008889 | 99008903 |
|  | chr14 | 101127854 | 101127865 |
|  | chr14 | 103628411 | 103628468 |
|  | chr14 | 103849146 | 103849298 |
|  | chr15 | 22682763 | 22682772 |
|  | chr15 | 23098501 | 23098547 |
|  | chr15 | 23940799 | 23940860 |
|  | chr15 | 27053379 | 27053390 |
|  | chr15 | 31528160 | 31528259 |
|  | chr15 | 41275214 | 41275230 |
|  | chr15 | 62464095 | 62464127 |
|  | chr15 | 66950617 | 66950649 |
|  | chr15 | 75415808 | 75415820 |
|  | chr15 | 77948128 | 77948189 |
|  | chr15 | 85640503 | 85640646 |
|  | chr15 | 91911749 | 91911821 |
|  | chr15 | 99226449 | 99226510 |
|  | chr16 | 189439 | 189467 |
|  | chr16 | 9610000 | 9610162 |
|  | chr16 | 12796326 | 12796364 |
|  | chr16 | 15797185 | 15797256 |
|  | chr16 | 17267062 | 17267179 |
|  | chr16 | 25189028 | 25189176 |
|  | chr16 | 32823496 | 32823508 |
|  | chr16 | 32961279 | 32961495 |
|  | chr16 | 33448764 | 33448822 |
|  | chr16 | 34344569 | 34344645 |
|  | chr16 | 48732290 | 48732327 |
|  | chr16 | 49047899 | 49047956 |
|  | chr16 | 51089726 | 51089744 |
|  | chr16 | 54158881 | 54158930 |
|  | chr16 | 56313403 | 56313461 |
|  | chr16 | 62238196 | 62238229 |
|  | chr16 | 71692810 | 71692854 |
|  | chr16 | 74876510 | 74876596 |
|  | chr16 | 75502781 | 75502801 |
|  | chr16 | 77997787 | 77997804 |
|  | chr16 | 78429138 | 78429228 |
|  | chr16 | 78611669 | 78611780 |
|  | chr16 | 79170022 | 79170154 |
|  | chr16 | 81642616 | 81642694 |
|  | chr16 | 86767668 | 86767680 |
|  | chr16 | 86835070 | 86835165 |
|  | chr16 | 87871072 | 87871088 |
|  | chr16 | 88044619 | 88044692 |
|  | chr16 | 89485596 | 89485637 |
|  | chr17 | 505327 | 505365 |
|  | chr17 | 4160089 | 4160137 |
|  | chr17 | 5077159 | 5077182 |
|  | chr17 | 6563850 | 6563957 |
|  | chr17 | 8214737 | 8214812 |
|  | chr17 | 11529051 | 11529187 |
|  | chr17 | 14198422 | 14198554 |
|  | chr17 | 33824594 | 33824707 |
|  | chr17 | 35128603 | 35128713 |
|  | chr17 | 48932075 | 48932199 |
|  | chr17 | 50026963 | 50027000 |
|  | chr17 | 53920863 | 53920928 |
|  | chr17 | 55543772 | 55543820 |
|  | chr17 | 55975321 | 55975363 |
|  | chr17 | 60782664 | 60782776 |
|  | chr17 | 61488668 | 61488809 |
|  | chr17 | 66611777 | 66611819 |
|  | chr17 | 78463744 | 78463764 |
|  | chr18 | 12007355 | 12007387 |
|  | chr18 | 12894341 | 12894353 |
|  | chr18 | 13610929 | 13610946 |
|  | chr18 | 39114686 | 39114742 |
|  | chr18 | 57215140 | 57215273 |
|  | chr18 | 59556016 | 59556079 |
|  | chr18 | 67954442 | 67954544 |
|  | chr18 | 68568419 | 68568549 |
|  | chr18 | 75332164 | 75332402 |
|  | chr18 | 77213900 | 77213925 |
|  | chr19 | 1176585 | 1176662 |
|  | chr19 | 1601584 | 1601690 |
|  | chr19 | 3828833 | 3828911 |
|  | chr19 | 10483772 | 10483818 |
|  | chr19 | 14950140 | 14950354 |
|  | chr19 | 22703282 | 22703353 |
|  | chr19 | 29201863 | 29201908 |
|  | chr19 | 40473485 | 40473521 |
|  | chr19 | 47471019 | 47471087 |
|  | chr19 | 55736827 | 55737002 |
|  | chr2 | 1800438 | 1800542 |
|  | chr2 | 2016400 | 2016465 |
|  | chr2 | 9036362 | 9036454 |
|  | chr2 | 9927289 | 9927338 |
|  | chr2 | 27301584 | 27301652 |
|  | chr2 | 37887033 | 37887053 |
|  | chr2 | 42368452 | 42368492 |
|  | chr2 | 45398091 | 45398174 |
|  | chr2 | 45953574 | 45953613 |
|  | chr2 | 46634181 | 46634217 |
|  | chr2 | 47064530 | 47064624 |
|  | chr2 | 49119124 | 49119278 |
|  | chr2 | 50396626 | 50396651 |
|  | chr2 | 55200786 | 55200967 |
|  | chr2 | 57987788 | 57987902 |
|  | chr2 | 62432409 | 62432440 |
|  | chr2 | 66323063 | 66323236 |
|  | chr2 | 72267857 | 72267960 |
|  | chr2 | 74123531 | 74123583 |
|  | chr2 | 100824335 | 100824366 |
|  | chr2 | 105997571 | 105997666 |
|  | chr2 | 108799097 | 108799181 |
|  | chr2 | 109789758 | 109789835 |
|  | chr2 | 120117387 | 120117430 |
|  | chr2 | 121301459 | 121301524 |
|  | chr2 | 123503669 | 123503721 |
|  | chr2 | 131857315 | 131857395 |
|  | chr2 | 132764097 | 132764270 |
|  | chr2 | 134277354 | 134277543 |
|  | chr2 | 134572006 | 134572111 |
|  | chr2 | 134846625 | 134846677 |
|  | chr2 | 143904813 | 143904945 |
|  | chr2 | 168759833 | 168759920 |
|  | chr2 | 169100883 | 169100918 |
|  | chr2 | 202986681 | 202986783 |
|  | chr2 | 205402995 | 205403172 |
|  | chr2 | 213948099 | 213948147 |
|  | chr2 | 217928517 | 217928698 |
|  | chr2 | 223433831 | 223433887 |
|  | chr2 | 224782320 | 224782348 |
|  | chr2 | 231742840 | 231742918 |
|  | chr2 | 232256318 | 232256429 |
|  | chr2 | 233178256 | 233178276 |
|  | chr2 | 234472536 | 234472593 |
|  | chr2 | 235451584 | 235451684 |
|  | chr2 | 235515636 | 235515744 |
|  | chr2 | 236011388 | 236011435 |
|  | chr2 | 237883691 | 237883737 |
|  | chr2 | 238832500 | 238832517 |
|  | chr2 | 239884996 | 239885051 |
|  | chr2 | 240366738 | 240366863 |
|  | chr2 | 241915001 | 241915058 |
|  | chr20 | 3004055 | 3004097 |
|  | chr20 | 4062811 | 4062848 |
|  | chr20 | 17154781 | 17154929 |
|  | chr20 | 20142431 | 20142487 |
|  | chr20 | 25234408 | 25234471 |
|  | chr20 | 30264675 | 30264775 |
|  | chr20 | 31222274 | 31222391 |
|  | chr20 | 32507639 | 32507709 |
|  | chr20 | 34860542 | 34860661 |
|  | chr20 | 35200913 | 35200963 |
|  | chr20 | 37456618 | 37456863 |
|  | chr20 | 40343892 | 40343934 |
|  | chr20 | 41066908 | 41067045 |
|  | chr20 | 44795213 | 44795294 |
|  | chr20 | 47779702 | 47779717 |
|  | chr20 | 49011341 | 49011402 |
|  | chr20 | 49965494 | 49965719 |
|  | chr20 | 50228091 | 50228257 |
|  | chr20 | 59385519 | 59385573 |
|  | chr20 | 60713549 | 60713587 |
|  | chr21 | 9828975 | 9829060 |
|  | chr21 | 16999542 | 16999593 |
|  | chr21 | 27500480 | 27500521 |
|  | chr21 | 29202193 | 29202207 |
|  | chr21 | 34905470 | 34905523 |
|  | chr21 | 35269274 | 35269343 |
|  | chr21 | 37021468 | 37021556 |
|  | chr21 | 40173464 | 40173529 |
|  | chr21 | 40550402 | 40550430 |
|  | chr21 | 41549614 | 41549813 |
|  | chr21 | 44216808 | 44216916 |
|  | chr21 | 47238367 | 47238393 |
|  | chr22 | 21613120 | 21613215 |
|  | chr22 | 24907861 | 24907932 |
|  | chr22 | 30054870 | 30054939 |
|  | chr22 | 41439883 | 41439927 |
|  | chr22 | 41900599 | 41900621 |
|  | chr22 | 50317212 | 50317249 |
|  | chr3 | 13388889 | 13388953 |
|  | chr3 | 32838704 | 32838763 |
|  | chr3 | 48565635 | 48565771 |
|  | chr3 | 70241292 | 70241360 |
|  | chr3 | 72335103 | 72335173 |
|  | chr3 | 72367558 | 72367672 |
|  | chr3 | 74683057 | 74683116 |
|  | chr3 | 99150699 | 99150892 |
|  | chr3 | 103459384 | 103459494 |
|  | chr3 | 109884602 | 109884625 |
|  | chr3 | 114776946 | 114777014 |
|  | chr3 | 120400574 | 120400661 |
|  | chr3 | 131361478 | 131361593 |
|  | chr3 | 135157770 | 135157927 |
|  | chr3 | 141202649 | 141202709 |
|  | chr3 | 183067334 | 183067446 |
|  | chr3 | 184934195 | 184934316 |
|  | chr3 | 191930766 | 191930911 |
|  | chr3 | 196569826 | 196569909 |
|  | chr4 | 468392 | 468434 |
|  | chr4 | 1525725 | 1525810 |
|  | chr4 | 6966041 | 6966067 |
|  | chr4 | 11906324 | 11906476 |
|  | chr4 | 16230204 | 16230237 |
|  | chr4 | 21755306 | 21755322 |
|  | chr4 | 40564544 | 40564577 |
|  | chr4 | 47102992 | 47103180 |
|  | chr4 | 55542581 | 55542680 |
|  | chr4 | 57017184 | 57017234 |
|  | chr4 | 65671462 | 65671493 |
|  | chr4 | 84284403 | 84284547 |
|  | chr4 | 85326978 | 85327102 |
|  | chr4 | 86395684 | 86395764 |
|  | chr4 | 87882341 | 87882399 |
|  | chr4 | 88138661 | 88138700 |
|  | chr4 | 90876216 | 90876258 |
|  | chr4 | 94500392 | 94500490 |
|  | chr4 | 119430068 | 119430106 |
|  | chr4 | 128089836 | 128089957 |
|  | chr4 | 133756881 | 133756909 |
|  | chr4 | 164113987 | 164114193 |
|  | chr4 | 165514697 | 165514828 |
|  | chr4 | 175008582 | 175008628 |
|  | chr4 | 177397694 | 177397789 |
|  | chr4 | 190606571 | 190606685 |
|  | chr4 | 190732230 | 190732312 |
|  | chr5 | 2613375 | 2613526 |
|  | chr5 | 3787278 | 3787298 |
|  | chr5 | 4199542 | 4199616 |
|  | chr5 | 6018649 | 6018682 |
|  | chr5 | 7879419 | 7879573 |
|  | chr5 | 8733788 | 8733871 |
|  | chr5 | 10527824 | 10527879 |
|  | chr5 | 14401560 | 14401591 |
|  | chr5 | 58633181 | 58633199 |
|  | chr5 | 68371806 | 68371940 |
|  | chr5 | 78498528 | 78498560 |
|  | chr5 | 90575462 | 90575486 |
|  | chr5 | 105100551 | 105100685 |
|  | chr5 | 113493150 | 113493334 |
|  | chr5 | 137815562 | 137815623 |
|  | chr5 | 148661343 | 148661421 |
|  | chr5 | 151292563 | 151292735 |
|  | chr5 | 159602685 | 159602798 |
|  | chr5 | 172288325 | 172288434 |
|  | chr5 | 174990357 | 174990482 |
|  | chr5 | 176506696 | 176506734 |
|  | chr5 | 177398421 | 177398538 |
|  | chr5 | 177716007 | 177716153 |
|  | chr6 | 325275 | 325309 |
|  | chr6 | 332867 | 333105 |
|  | chr6 | 815050 | 815104 |
|  | chr6 | 2629698 | 2629766 |
|  | chr6 | 7263770 | 7263806 |
|  | chr6 | 10522178 | 10522198 |
|  | chr6 | 17779269 | 17779311 |
|  | chr6 | 17932531 | 17932659 |
|  | chr6 | 17971892 | 17971983 |
|  | chr6 | 34494678 | 34494779 |
|  | chr6 | 35307307 | 35307338 |
|  | chr6 | 35987222 | 35987310 |
|  | chr6 | 36984720 | 36984992 |
|  | chr6 | 42845331 | 42845348 |
|  | chr6 | 43757775 | 43757973 |
|  | chr6 | 47198579 | 47198686 |
|  | chr6 | 64132006 | 64132074 |
|  | chr6 | 70990706 | 70990738 |
|  | chr6 | 78446340 | 78446534 |
|  | chr6 | 85838948 | 85839091 |
|  | chr6 | 90872582 | 90872736 |
|  | chr6 | 98003097 | 98003177 |
|  | chr6 | 104797136 | 104797212 |
|  | chr6 | 106517489 | 106517547 |
|  | chr6 | 108171091 | 108171132 |
|  | chr6 | 110673909 | 110673959 |
|  | chr6 | 119646811 | 119646821 |
|  | chr6 | 128812621 | 128812704 |
|  | chr6 | 134061470 | 134061589 |
|  | chr6 | 137037115 | 137037163 |
|  | chr6 | 137605701 | 137605911 |
|  | chr6 | 143597951 | 143598096 |
|  | chr6 | 153170236 | 153170294 |
|  | chr6 | 159224349 | 159224372 |
|  | chr6 | 159864434 | 159864563 |
|  | chr6 | 161187759 | 161187811 |
|  | chr6 | 161691354 | 161691541 |
|  | chr6 | 164526702 | 164526745 |
|  | chr6 | 168778383 | 168778409 |
|  | chr6 | 169733248 | 169733305 |
|  | chr7 | 2311438 | 2311459 |
|  | chr7 | 2705036 | 2705053 |
|  | chr7 | 4185818 | 4185887 |
|  | chr7 | 5854737 | 5854755 |
|  | chr7 | 6617523 | 6617559 |
|  | chr7 | 6704235 | 6704261 |
|  | chr7 | 20434944 | 20435040 |
|  | chr7 | 20600495 | 20600508 |
|  | chr7 | 28428634 | 28428754 |
|  | chr7 | 35239302 | 35239323 |
|  | chr7 | 37298913 | 37298996 |
|  | chr7 | 38669773 | 38669841 |
|  | chr7 | 48949031 | 48949169 |
|  | chr7 | 51703943 | 51703972 |
|  | chr7 | 56263916 | 56264011 |
|  | chr7 | 66310503 | 66310513 |
|  | chr7 | 67642010 | 67642085 |
|  | chr7 | 69946285 | 69946340 |
|  | chr7 | 70834437 | 70834539 |
|  | chr7 | 70985711 | 70985801 |
|  | chr7 | 71566812 | 71566842 |
|  | chr7 | 86633599 | 86633715 |
|  | chr7 | 91187345 | 91187435 |
|  | chr7 | 98716538 | 98716593 |
|  | chr7 | 99915490 | 99915533 |
|  | chr7 | 102083743 | 102083767 |
|  | chr7 | 122474413 | 122474428 |
|  | chr7 | 126898187 | 126898203 |
|  | chr7 | 127526227 | 127526283 |
|  | chr7 | 131495014 | 131495054 |
|  | chr7 | 133340859 | 133340883 |
|  | chr7 | 135651388 | 135651566 |
|  | chr7 | 138661198 | 138661276 |
|  | chr7 | 144135927 | 144136012 |
|  | chr7 | 151090963 | 151091033 |
|  | chr7 | 151855965 | 151856050 |
|  | chr7 | 152130922 | 152130974 |
|  | chr7 | 154011588 | 154011651 |
|  | chr7 | 154216049 | 154216171 |
|  | chr7 | 155284061 | 155284082 |
|  | chr8 | 335340 | 335383 |
|  | chr8 | 542462 | 542543 |
|  | chr8 | 8232950 | 8232965 |
|  | chr8 | 10236812 | 10236915 |
|  | chr8 | 13078748 | 13078905 |
|  | chr8 | 17552637 | 17552657 |
|  | chr8 | 20548524 | 20548720 |
|  | chr8 | 23890837 | 23890958 |
|  | chr8 | 25819601 | 25819629 |
|  | chr8 | 29965101 | 29965128 |
|  | chr8 | 30562403 | 30562414 |
|  | chr8 | 33395227 | 33395266 |
|  | chr8 | 41377333 | 41377532 |
|  | chr8 | 49427867 | 49427913 |
|  | chr8 | 56430631 | 56430738 |
|  | chr8 | 58943001 | 58943104 |
|  | chr8 | 74282972 | 74283002 |
|  | chr8 | 74784306 | 74784374 |
|  | chr8 | 86937232 | 86937357 |
|  | chr8 | 92735654 | 92735778 |
|  | chr8 | 94766430 | 94766471 |
|  | chr8 | 97290117 | 97290168 |
|  | chr8 | 103541409 | 103541475 |
|  | chr8 | 103573566 | 103573665 |
|  | chr8 | 112757941 | 112758093 |
|  | chr8 | 120613848 | 120613894 |
|  | chr8 | 120667457 | 120667542 |
|  | chr8 | 128439250 | 128439294 |
|  | chr8 | 128920301 | 128920365 |
|  | chr8 | 137214421 | 137214521 |
|  | chr8 | 137458010 | 137458124 |
|  | chr8 | 139239553 | 139239731 |
|  | chr9 | 13529631 | 13529712 |
|  | chr9 | 14240600 | 14240713 |
|  | chr9 | 28509600 | 28509629 |
|  | chr9 | 33315136 | 33315324 |
|  | chr9 | 77764271 | 77764307 |
|  | chr9 | 79168087 | 79168151 |
|  | chr9 | 88057323 | 88057462 |
|  | chr9 | 91147031 | 91147061 |
|  | chr9 | 98075729 | 98075795 |
|  | chr9 | 101449533 | 101449597 |
|  | chr9 | 103375196 | 103375265 |
|  | chr9 | 103375285 | 103375379 |
|  | chr9 | 104556808 | 104556902 |
|  | chr9 | 105521959 | 105522088 |
|  | chr9 | 108814196 | 108814282 |
|  | chr9 | 111929739 | 111929762 |
|  | chr9 | 116108873 | 116108913 |
|  | chr9 | 120373900 | 120373925 |
|  | chr9 | 127147983 | 127148111 |
|  | chr9 | 129404657 | 129404682 |
|  | chr9 | 132106821 | 132106867 |
|  | chr9 | 132256066 | 132256143 |
|  | chr9 | 135290184 | 135290222 |
|  | chr9 | 138797064 | 138797111 |
|  | chr9 | 139528165 | 139528189 |
|  | chr9 | 139682570 | 139682599 |
|  | chr9 | 140542339 | 140542351 |
|  | chr9 | 140572840 | 140572939 |
|  | chrX | 766752 | 766834 |
|  | chrX | 1248047 | 1248158 |
|  | chrX | 2931776 | 2931906 |
|  | chrX | 3941711 | 3941901 |
|  | chrX | 5900830 | 5900954 |
|  | chrX | 8357504 | 8357699 |
|  | chrX | 9879589 | 9879605 |
|  | chrX | 12820746 | 12820760 |
|  | chrX | 20431165 | 20431260 |
|  | chrX | 23761882 | 23761919 |
|  | chrX | 39765126 | 39765175 |
|  | chrX | 43463042 | 43463272 |
|  | chrX | 63615379 | 63615413 |
|  | chrX | 63830511 | 63830631 |
|  | chrX | 66514108 | 66514224 |
|  | chrX | 73462544 | 73462599 |
|  | chrX | 112049549 | 112049633 |
|  | chrX | 120289310 | 120289334 |
|  | chrX | 121624351 | 121624548 |
|  | chrX | 125540741 | 125540775 |
|  | chrX | 136953539 | 136953681 |
|  | chrX | 142077778 | 142077903 |
|  | chrX | 149404040 | 149404099 |
|  | chrX | 150953545 | 150953601 |
|  | chrX | 151142148 | 151142221 |

**TABLE S5 Characteristics of fifteen methylation markers and their coefficients in early detection of breast cancer.**

| **Marker** | **Chromosome** | **Start** | **End** | **Length** | **Mean methylation difference (cancer - control)** | **P value** |
| --- | --- | --- | --- | --- | --- | --- |
| Region1 | chr10 | 39014665 | 39014692 | 28 | -0.211015 | 1.62E-05 |
| Region2 | chr11 | 127215594 | 127215624 | 31 | -0.3297515 | 9.57E-06 |
| Region3 | chr13 | 95459772 | 95459798 | 27 | -0.2184069 | 4.88E-06 |
| Region4 | chr16 | 32823496 | 32823508 | 13 | 0.2721491 | 4.15E-06 |
| Region5 | chr16 | 77997787 | 77997804 | 18 | -0.265768 | 9.49E-06 |
| Region6 | chr3 | 135157770 | 135157927 | 158 | -0.2113162 | 3.12E-05 |
| Region7 | chr6 | 153170236 | 153170294 | 59 | -0.2116533 | 2.13E-05 |
| Region8 | chr6 | 161187759 | 161187811 | 53 | -0.2912524 | 3.19E-04 |
| Region9 | chr7 | 124319970 | 124320207 | 238 | -0.2965146 | 9.26E-05 |
| Region10 | chr7 | 51703943 | 51703972 | 30 | -0.2514395 | 1.54E-05 |
| Region11 | chr7 | 70979852 | 70979954 | 103 | -0.258864 | 1.79E-06 |
| Region12 | chr7 | 70985711 | 70985801 | 91 | -0.327665 | 8.34E-07 |
| Region13 | chr7 | 91187345 | 91187435 | 91 | -0.2582435 | 7.48E-06 |
| Region14 | chr8 | 55124674 | 55124771 | 98 | -0.2272259 | 6.30E-04 |
| Region15 | chrX | 8357504 | 8357699 | 196 | -0.2182057 | 6.89E-04 |

**TABLE S6 Clinical characteristics of patients in training set, test set 1, and test set 2.**

| **Characteristics** | **Training set**  (n=38) | **Test set 1**  (n=15) | **Test set 2**  (n=70) |
| --- | --- | --- | --- |
| Age, yr/o ^a^ | 50.87±11.05 | 53.47±8.88 | 50.7±10.31 |
| Menopausal status, No. (%) |  | | |
| Premenopausal | 14 (36.84) | 6 (40.00) | 22 (31.43) |
| Postmenopausal | 23 (60.53) | 9 (60.00) | 46 (65.71) |
| NA | 1 (2.63) | 0 (0) | 2 (2.86) |
| Tumor (T) ^b^, No. (%) |  | | |
| Tis | 1 (2.63) | 1 (6.67) | 0 (0) |
| T1 | 14 (36.84) | 9 (60.00) | 8 (11.43) |
| T2 | 15 (39.47) | 5 (33.33) | 42 (60.00) |
| T3 | 4 (10.53) | 0 (0) | 4 (5.71) |
| T4 | 3 (7.89) | 0 (0) | 11 (15.71) |
| Tx | 1 (2.63) | 0 (0) | 5 (7.14) |
| Nodes (N) ^b^, No. (%) |  | | |
| N0 | 21 (55.26) | 9 (60.00) | 22 (31.43) |
| N1 | 9 (23.68) | 3 (20.00) | 22 (31.43) |
| N2 | 1 (2.63) | 2 (13.33) | 4 (5.71) |
| N3 | 7 (18.42) | 1 (6.67) | 21 (30.00) |
| Nx | 0 (0) | 0 (0) | 1 (1.43) |
| Metastasis (M) ^b^, No. (%) |  | | |
| M0 | 38 (100) | 15 (100) | 0 (0) |
| M1 | 0 (0) | 0 (0) | 70 (100) |
| Stage ^b^, No. (%) |  | | |
| I | 10 (26.32) | 8 (53.33) | 0 (0) |
| II | 18 (47.37) | 4 (26.67) | 0 (0) |
| III | 10 (26.32) | 3 (20.00) | 0 (0) |
| IV | 0 (0) | 0 (0) | 70 (100%) |
| Histology, No. (%) |  | | |
| Ductal carcinoma in situ | 1 (2.63) | 1 (6.67) | 0 (0) |
| Invasive cacinoma | 36 (94.74) | 14 (93.33) | 69 (98.57) |
| Other | 1 (2.63) | 0 (0) | 1 (1.43) |
| Estrogen receptor (ER), No. (%) |  |  |  |
| ER positive | 22 (57.89) | 11 (73.33) | 47 (67.14) |
| ER negative | 16 (42.11) | 4 (26.67) | 23 (32.86) |
| NA | 0 (0) | 0 (0) | 0 (0) |
| Progesterone receptor (PR), No. (%) |  | | |
| PR positive | 16 (42.11) | 11 (73.33) | 37 (52.86) |
| PR negative | 22 (57.89) | 4 (26.67) | 31 (44.29) |
| NA | 0 (0) | 0 (0) | 2 (2.86) |
| HER2 status, No. (%) |  | | |
| HER2 positive | 9 (23.68) | 6 (40.00) | 14 (20.00) |
| HER2 negative | 28 (73.68) | 9 (60.00) | 55 (78.57) |
| NA | 1 (2.63) | 0 (0) | 1 (1.43) |
| Molecular subtype, No. (%) |  | | |
| ER^+^/HER2^-^ | 18 (47.37) | 9 (60.00) | 38 (54.29) |
| ER^+^/HER2^+^ | 3 (7.89) | 2 (13.33) | 9 (12.86) |
| ER^-^/HER2^+^ | 6 (15.79) | 4 (26.67) | 5 (7.14) |
| TNBC | 10 (26.32) | 0 (0) | 17 (24.29) |
| NA | 1 (2.63) | 0 (0) | 1 (1.43) |
| CA153, No. (%) |  | | |
| Positive | 2 (5.26) | 0 (0) | 48 (68.57) |
| Negative | 35 (92.11) | 15 (100) | 22 (31.43) |
| NA | 1 (2.63) | 0 (0) | 0 (0) |

^a^ Data is shown as mean ± standard deviation (SD).

^b^ The primary tumor, lymph node, and metastasis (TNM) staging was determined according to the eighth edition of classification for breast cancer of the American Joint Commission of Cancer (AJCC).

Abbreviation: n, No., number; yr/o, year(s) old; NA, not available.

**TABLE S7 Demographic characteristics of patients and controls.**

| **Characteristics** | **Training set** | | **Test set 1** | | **Test set 2** | |
| --- | --- | --- | --- | --- | --- | --- |
|  | Cohort 1 (n=38) | Controls (n=25) | Cohort 2 (n=15) | Controls (n=15) | Cohort 3 (n=70) | Controls (n=15) |
| Age, yr/o ^a^ | 50.87±11.05 | 44.5±10.55 | 53.47±8.88 | 42.33±8.51 | 50.7±10.31 | 42.33±8.51 |
| Menopausal status, No. (%) |  | | | | | |
| Premenopausal | 14 (36.84) | 18 (72.00) | 6 (40.00) | 9 (60.00) | 22 (31.43) | 9 (60.00) |
| Postmenopausal | 23 (60.53) | 7 (28.00) | 9 (60.00) | 4 (27.00) | 46 (65.71) | 4 (27.00) |
| NA | 1 (2.63) | 0 (0) | 0 (0) | 2 (13.00) | 2 (2.86) | 2 (13.00) |

^a^ Data is shown as mean ± standard deviation (SD).

Abbreviation: n, No., number; yr/o, year(s) old; NA, not available

**Table S8** **The samples information of the ddPCR assays.**

| **Serial ID** | **Samples Name** | **Stage** | **Methylated copies/μL** | **Non-Methylated copies/μL** | **Methylation Level** |
| --- | --- | --- | --- | --- | --- |
|  | TZ88 | Tumor | 6.17 | 17.4 | 0.26 |
|  | TZ107 | Tumor | 17.1 | 61.6 | 0.24 |
|  | TZ113 | Tumor | 3.3 | 11.8 | 0.21 |
|  | TZ114 | Tumor | 19.7 | 62.8 | 0.23 |
|  | TZ155 | Tumor | 15.8 | 42.8 | 0.26 |
|  | TZ158 | Tumor | 0.977 | 1.73 | 0.34 |
|  | XH95 | Tumor | 14.2 | 127 | 0.1 |
|  | XH101 | Tumor | 16.3 | 115 | 0.12 |
|  | Z7 | Normal | 0.364 | 0.8 | 0.33 |
|  | N0007 | Normal | 0.838 | 1.01 | 0.46 |
|  | N0010 | Normal | 11.1 | 22.8 | 0.34 |
|  | N0015 | Normal | 7 | 9.34 | 0.43 |
|  | N0025 | Normal | 0.591 | 1.08 | 0.35 |

**TABLE S9 The 883 Samples information of TCGA HM450K data.**


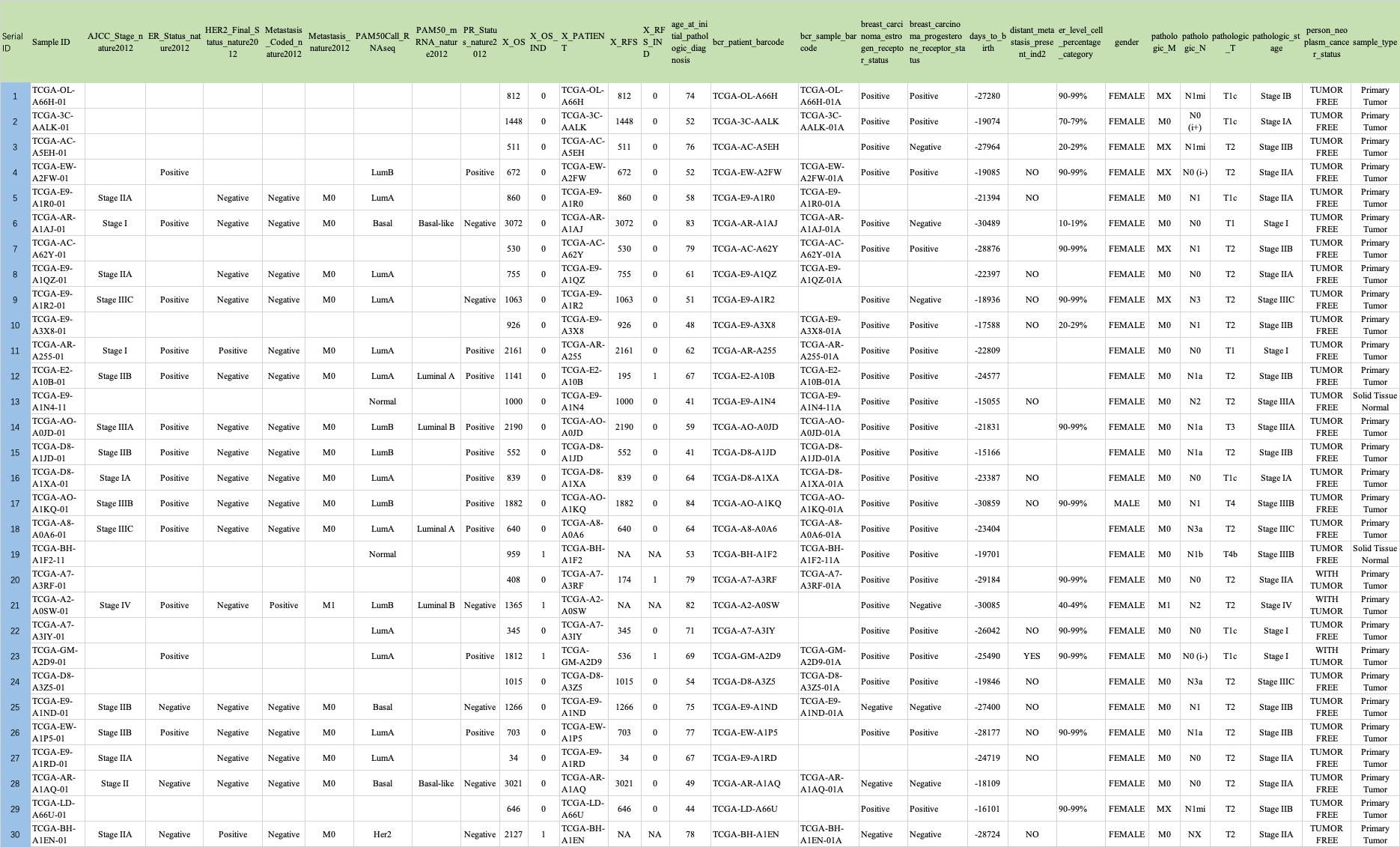


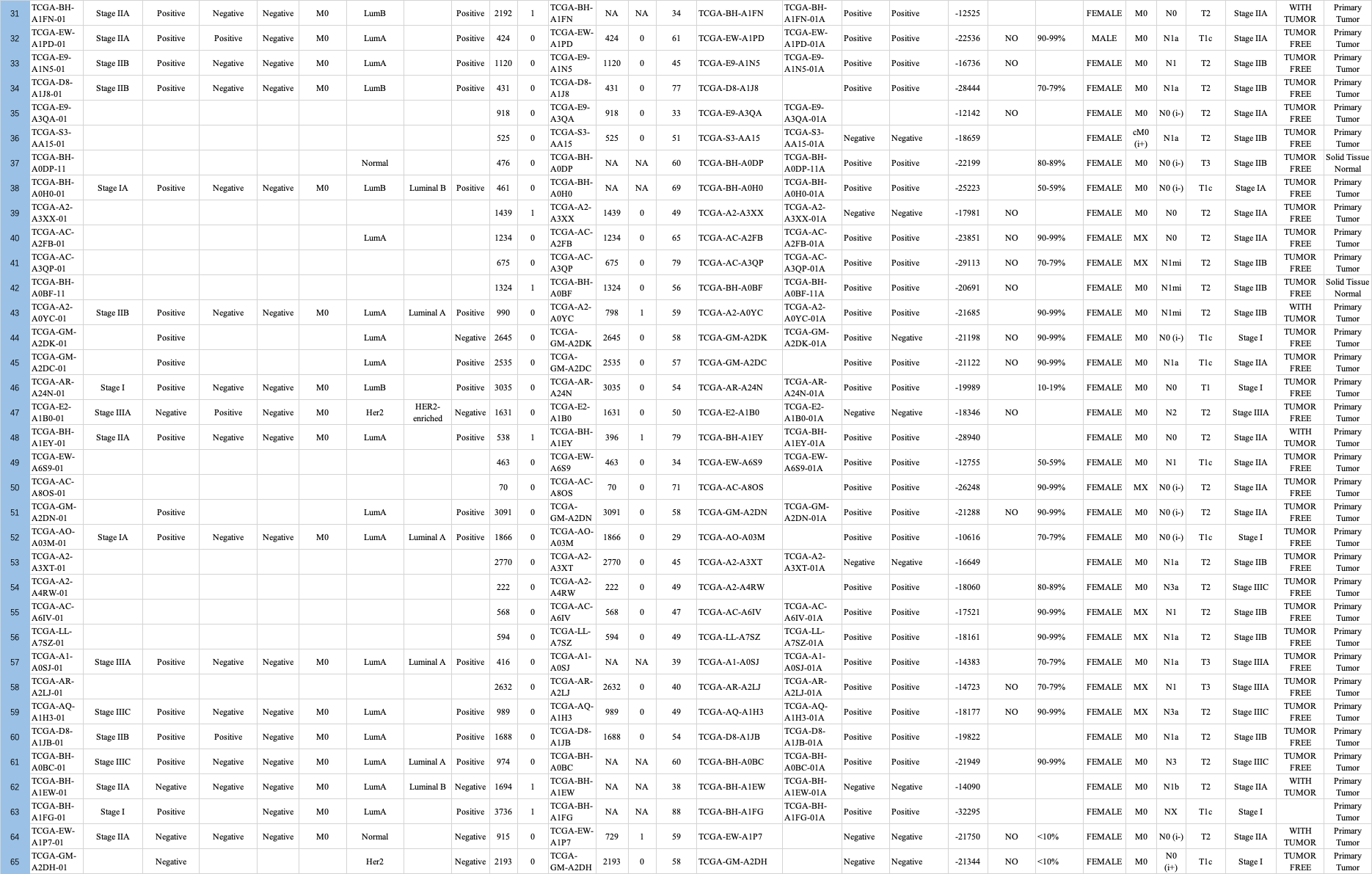


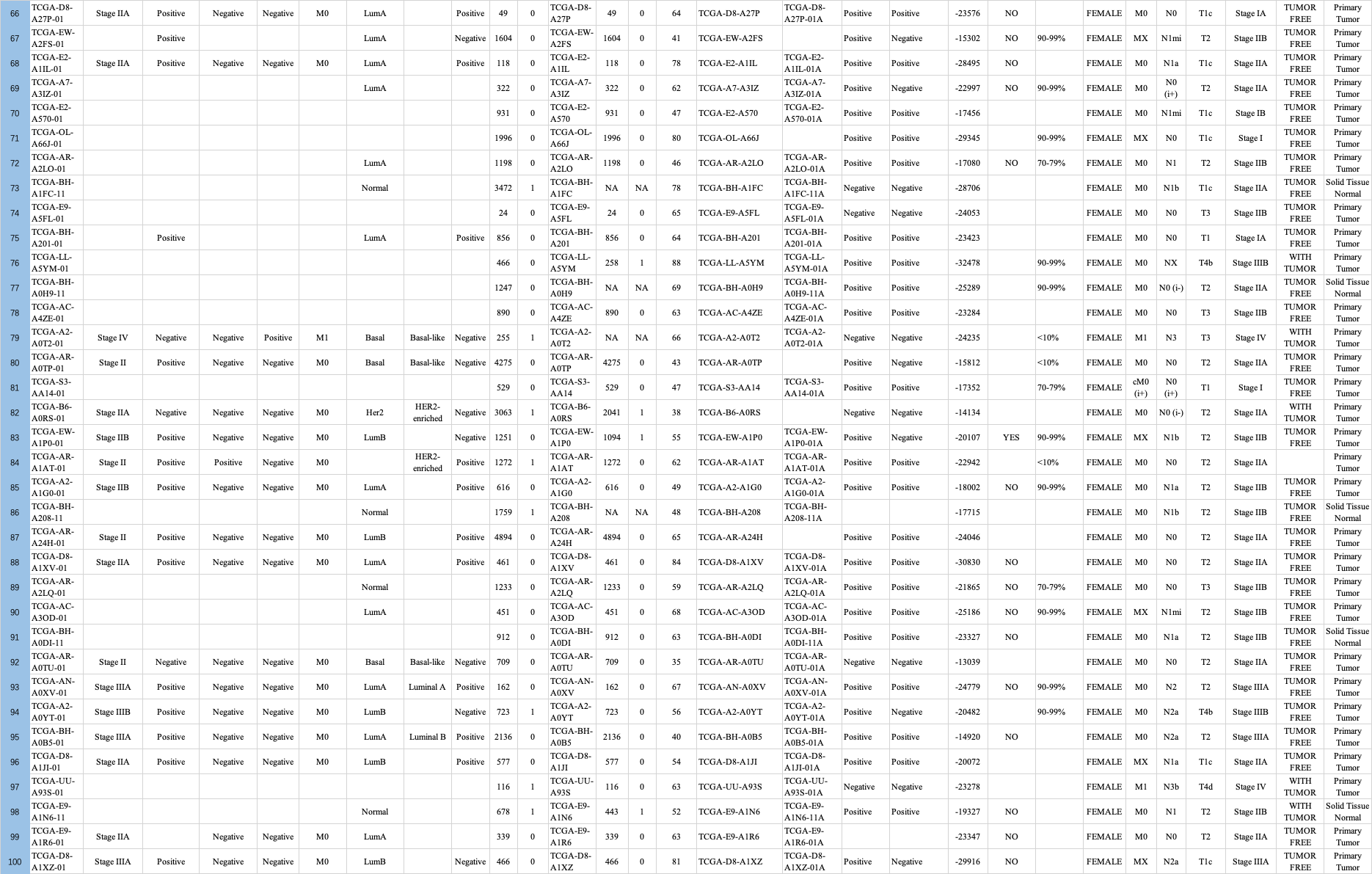


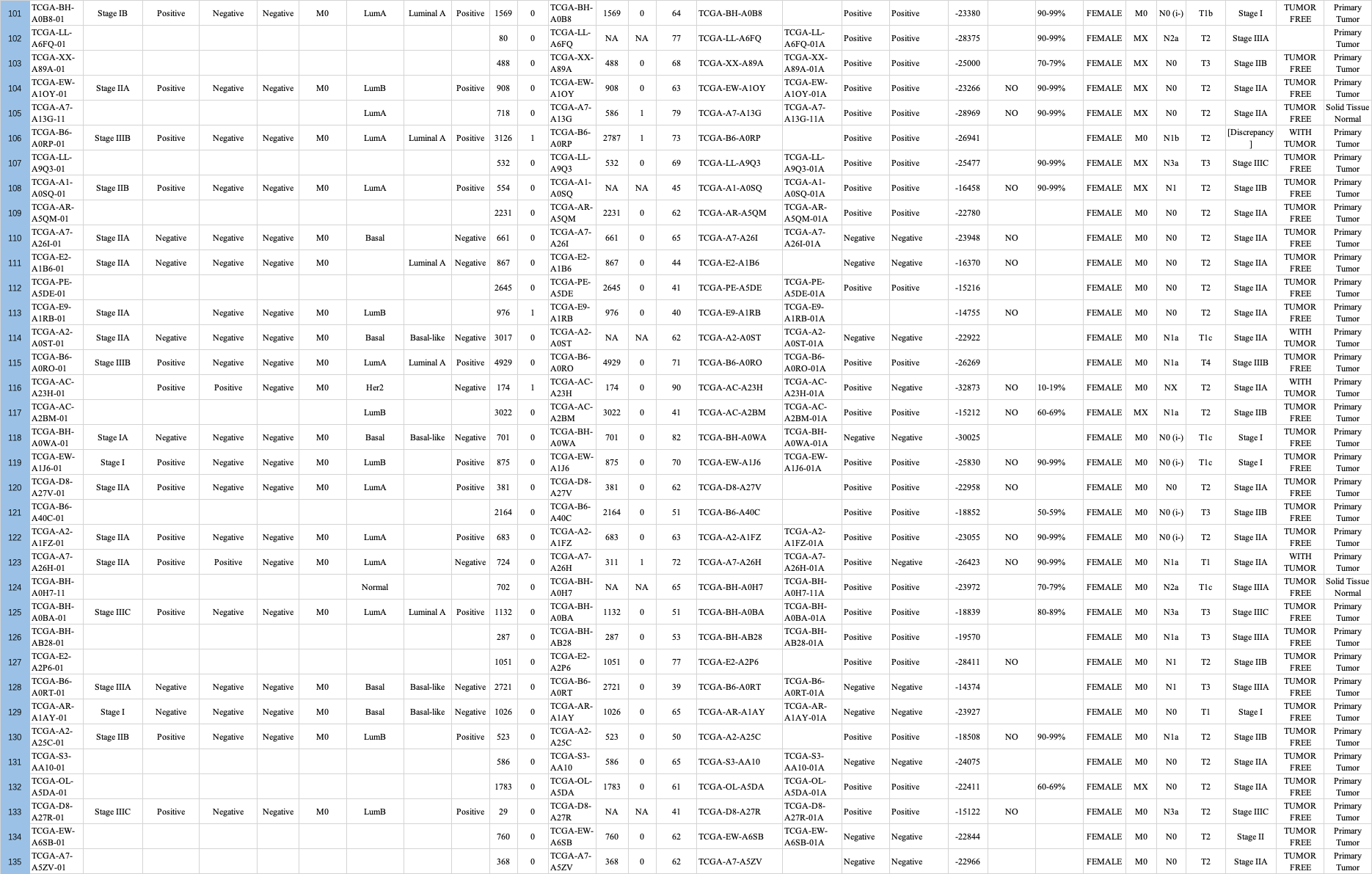


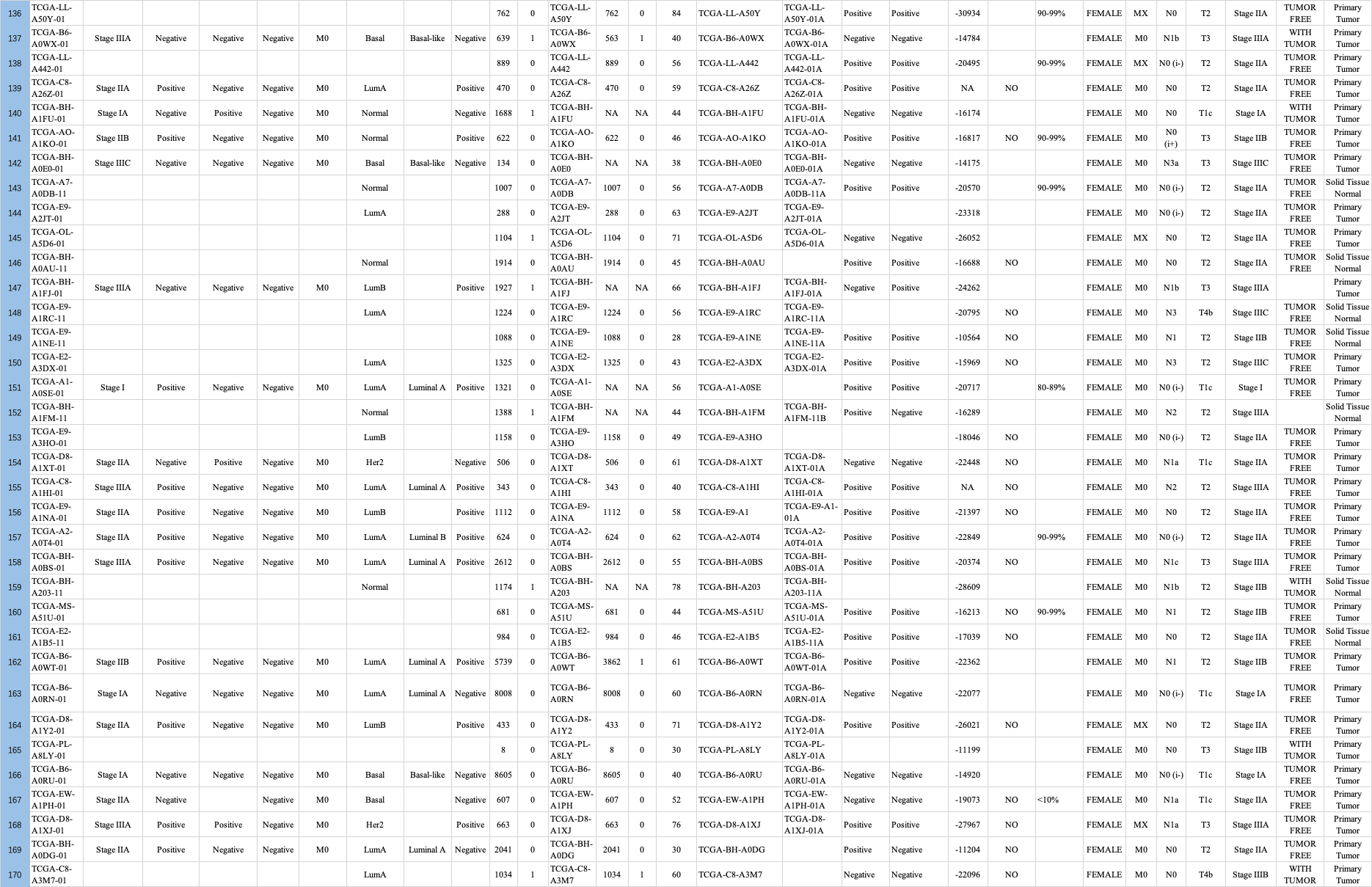


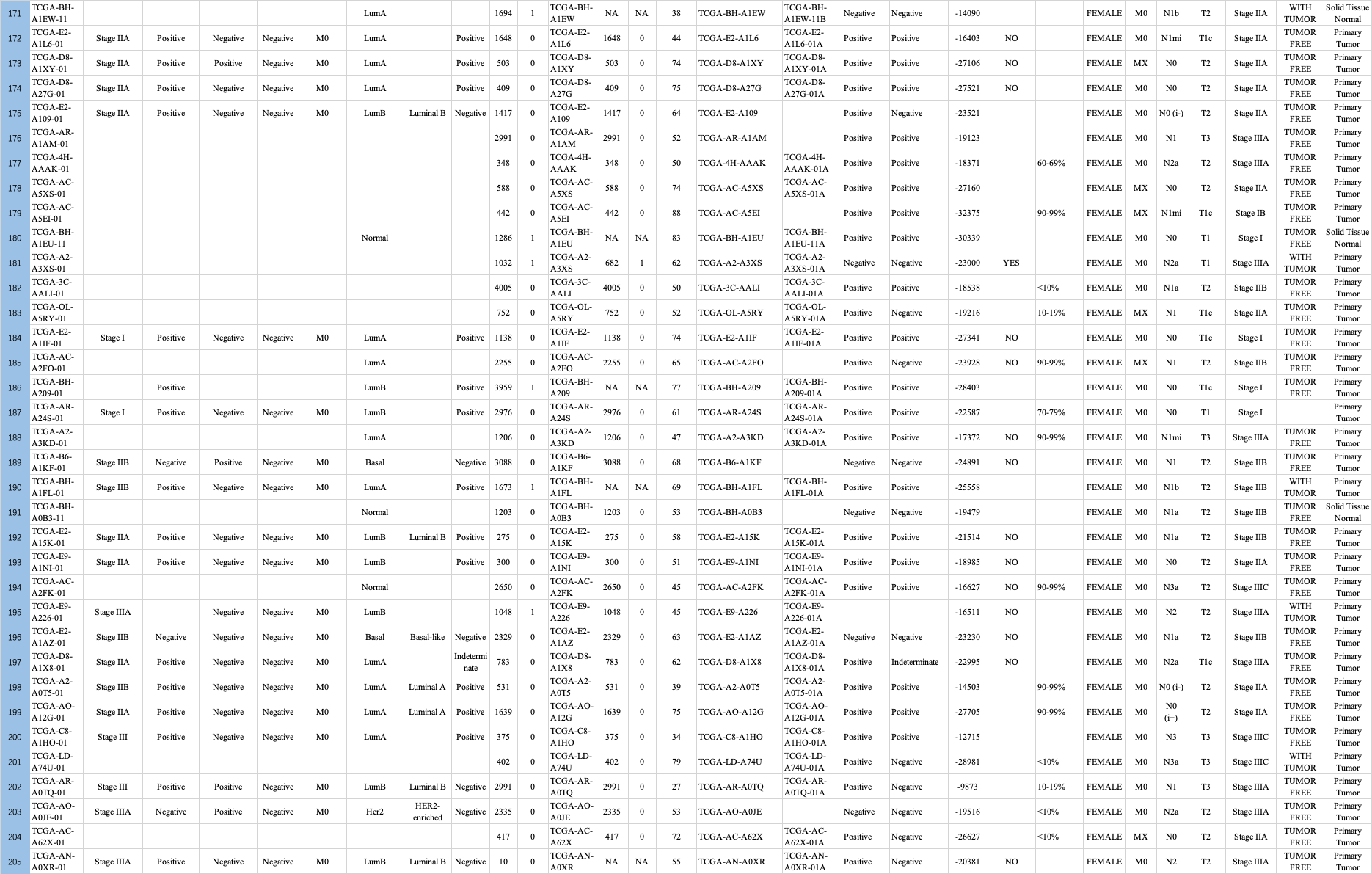


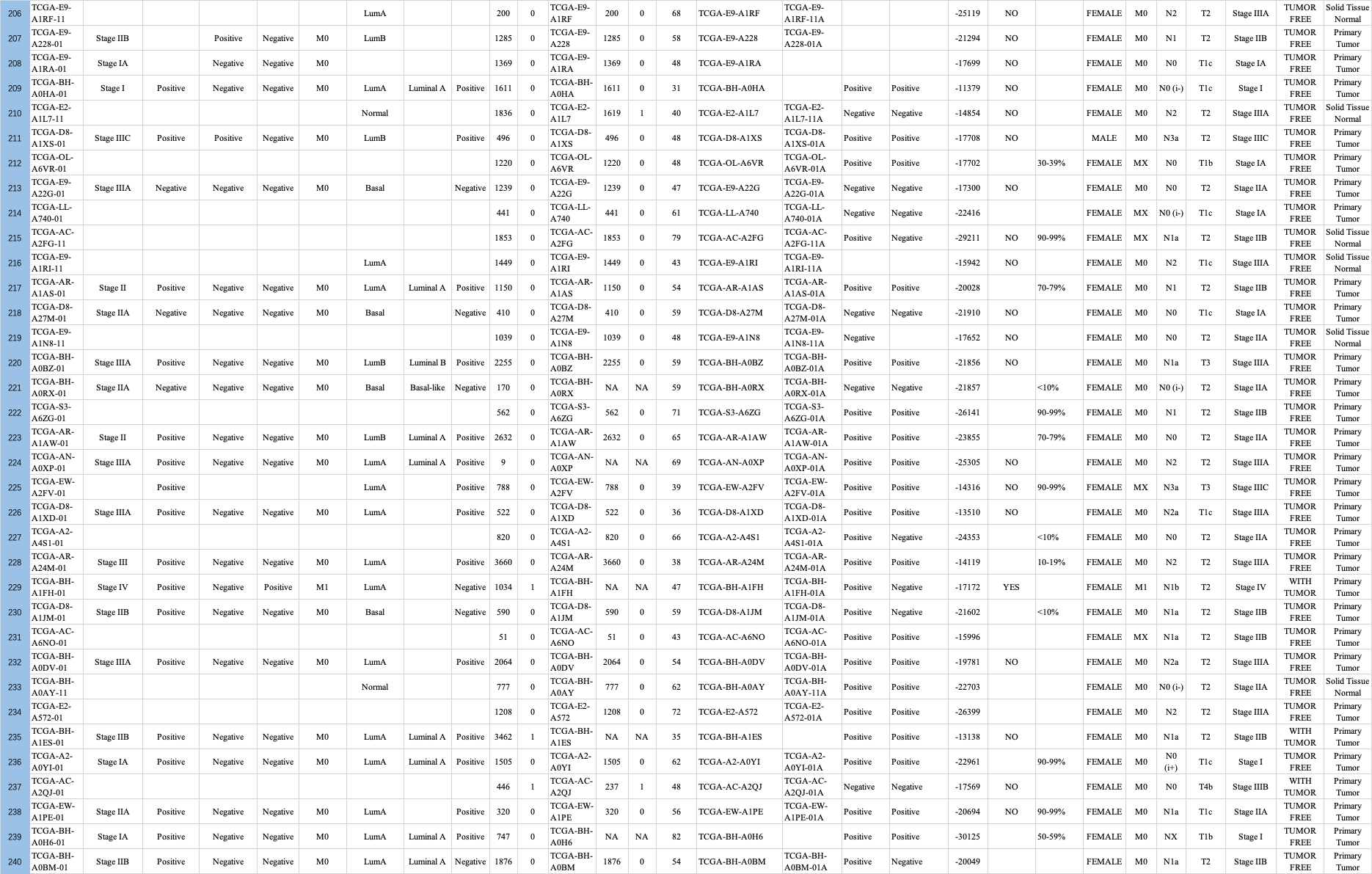


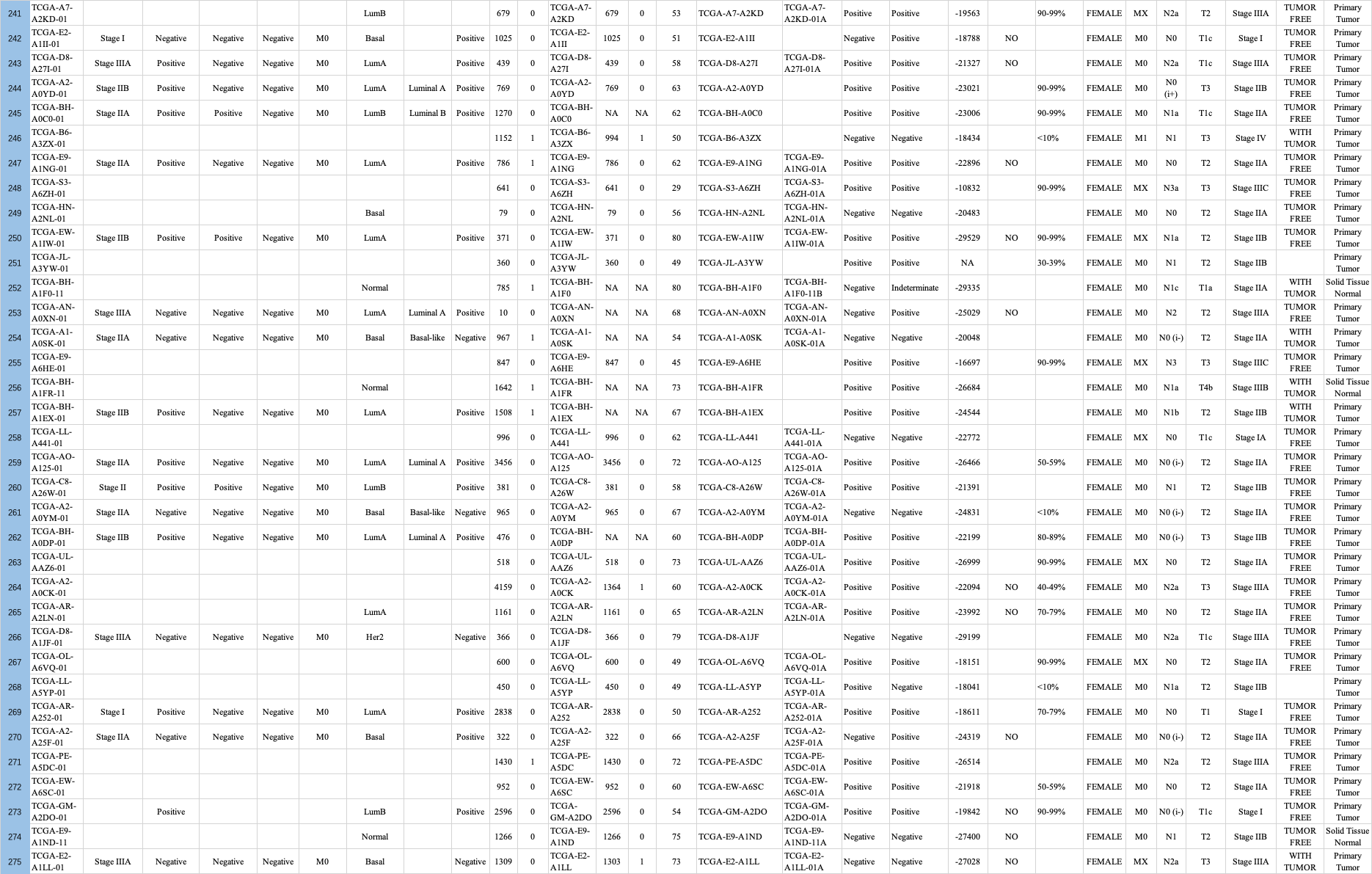


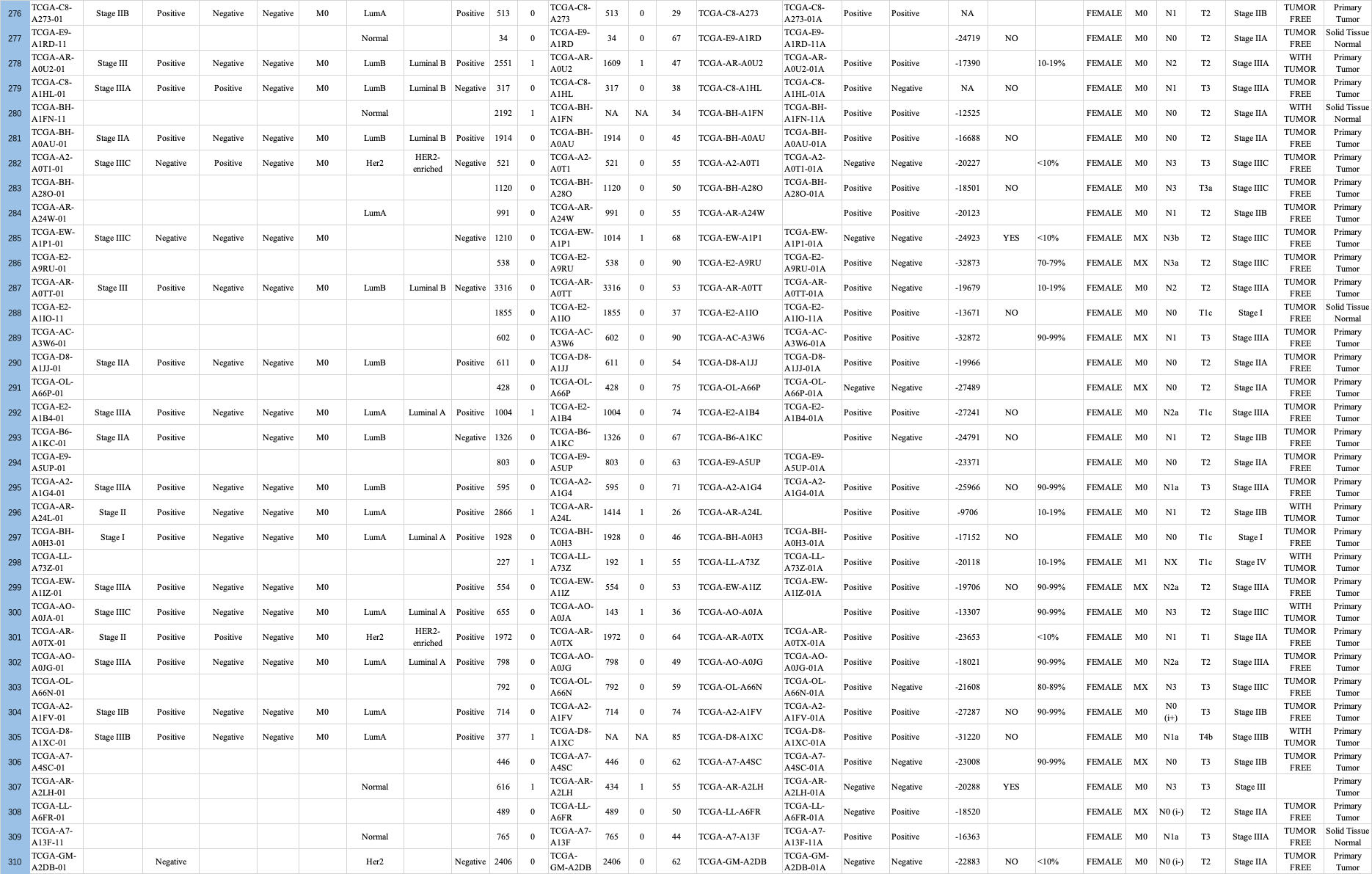


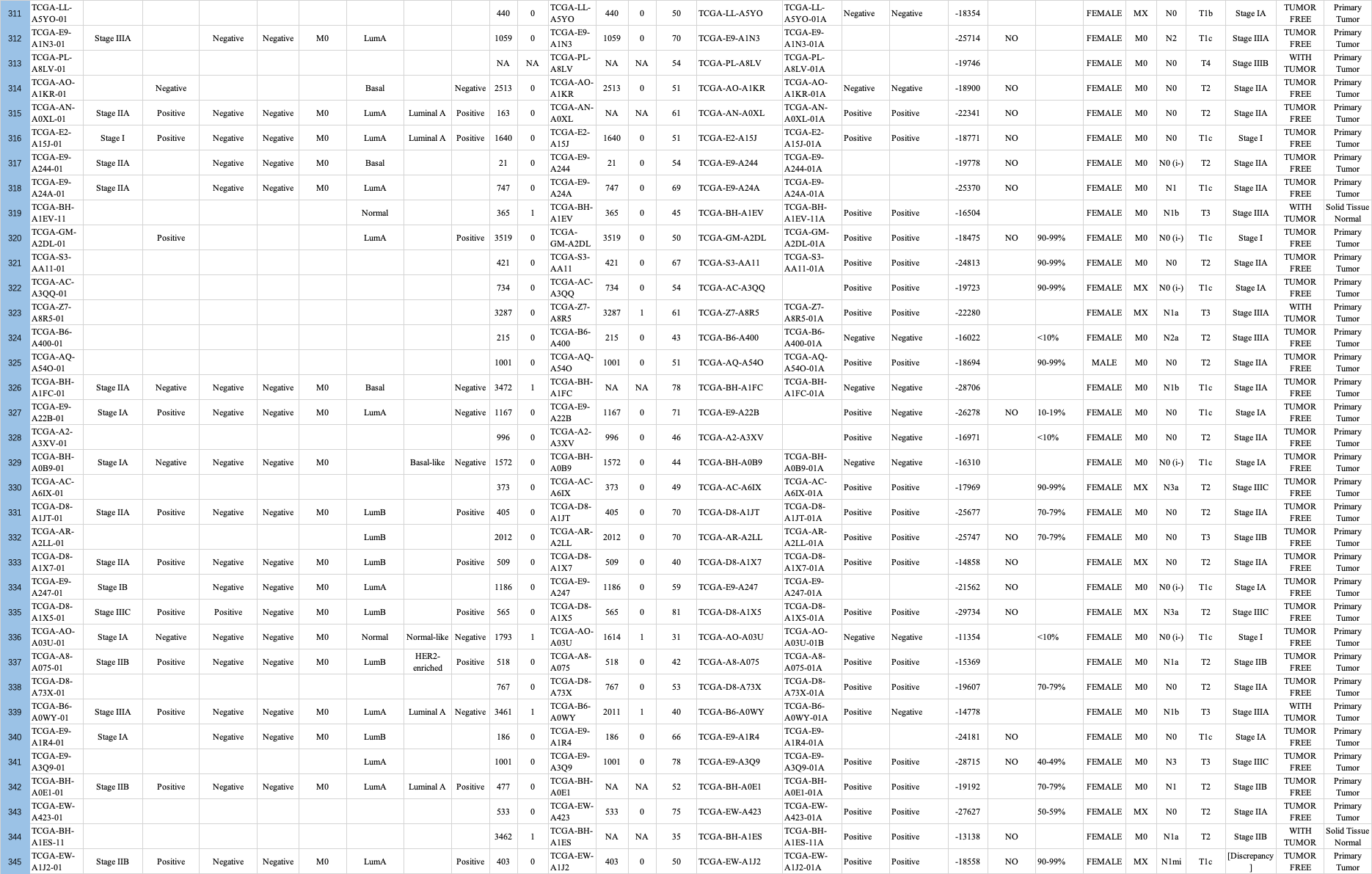


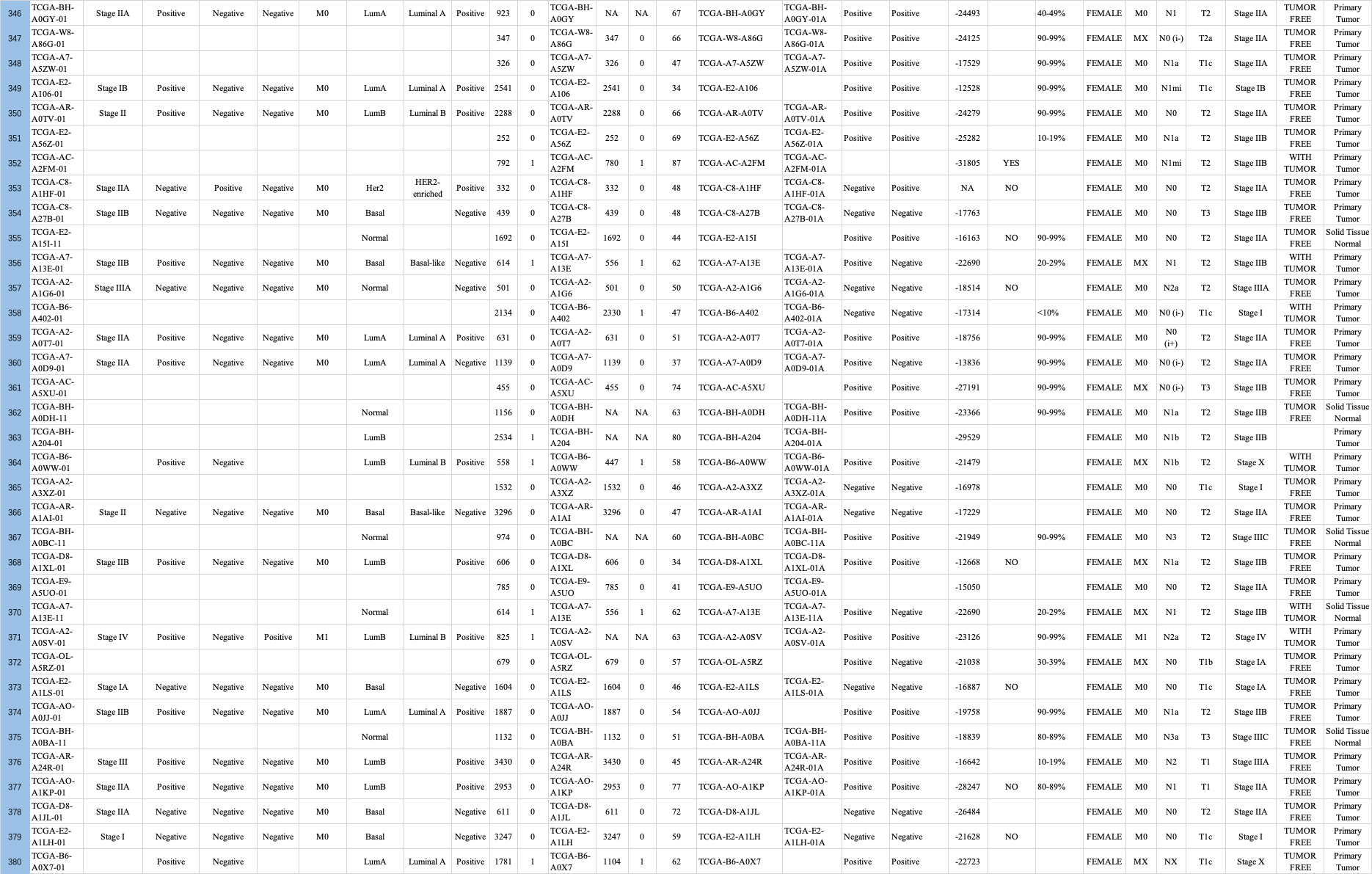


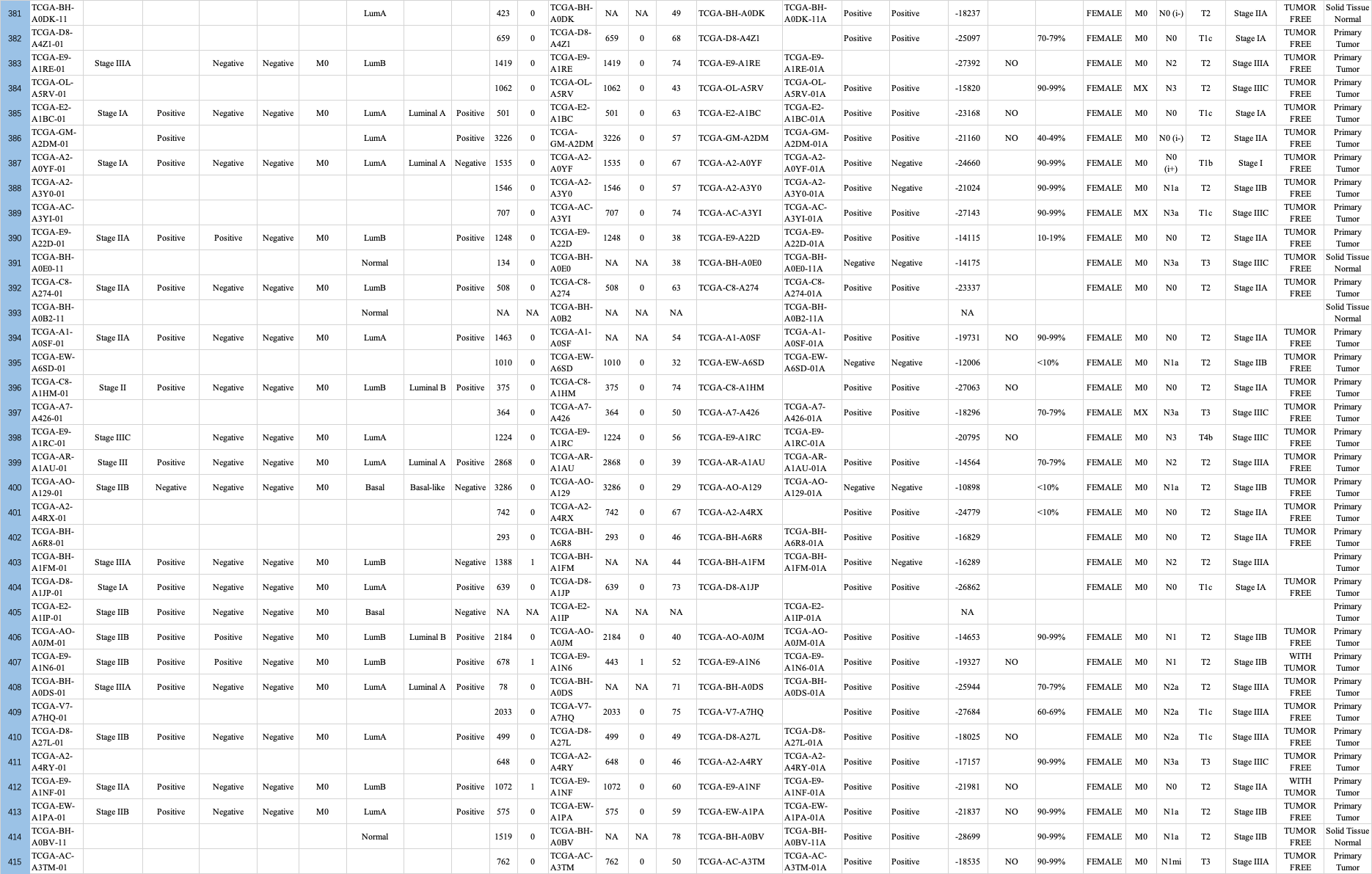


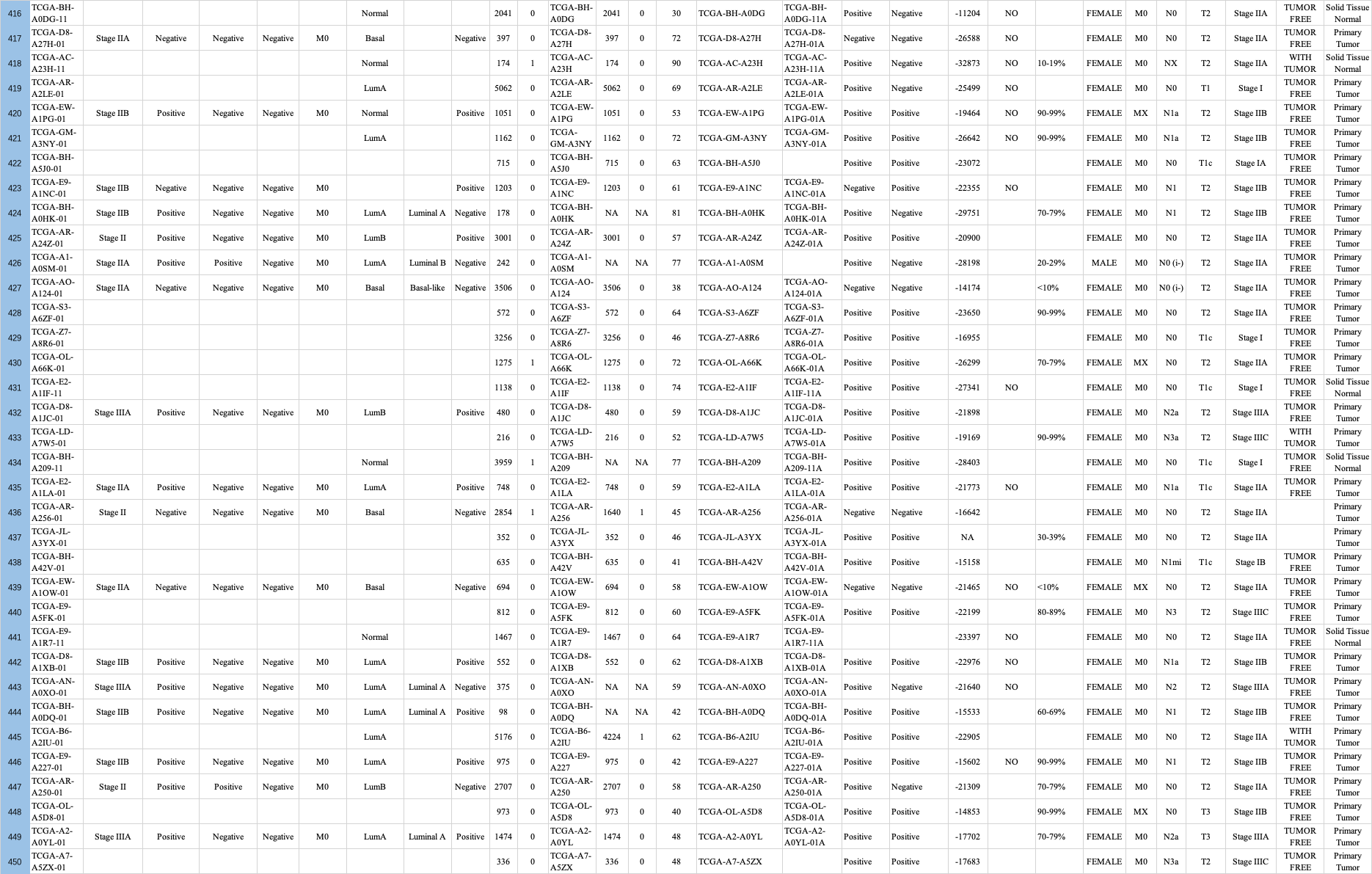


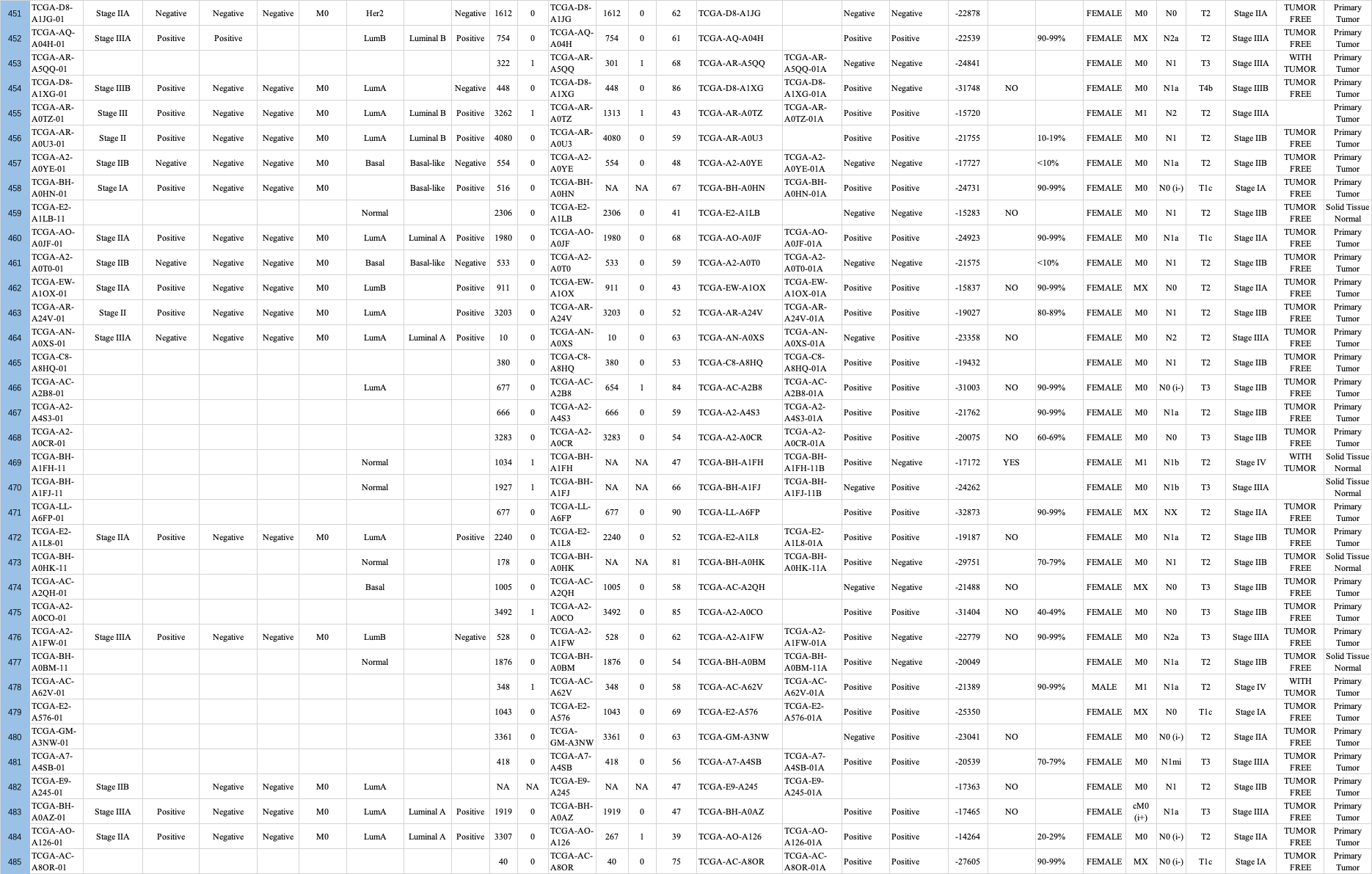


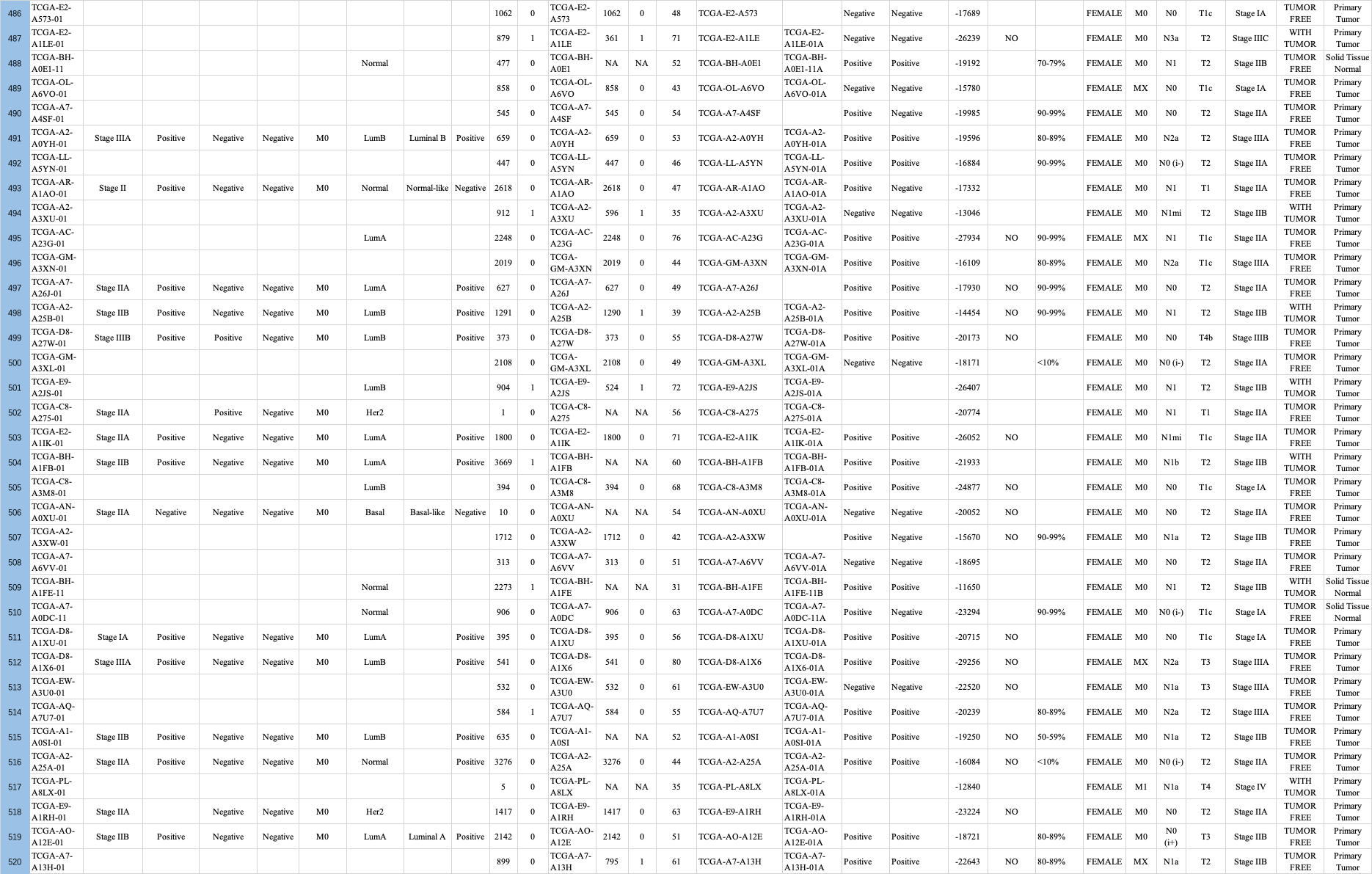


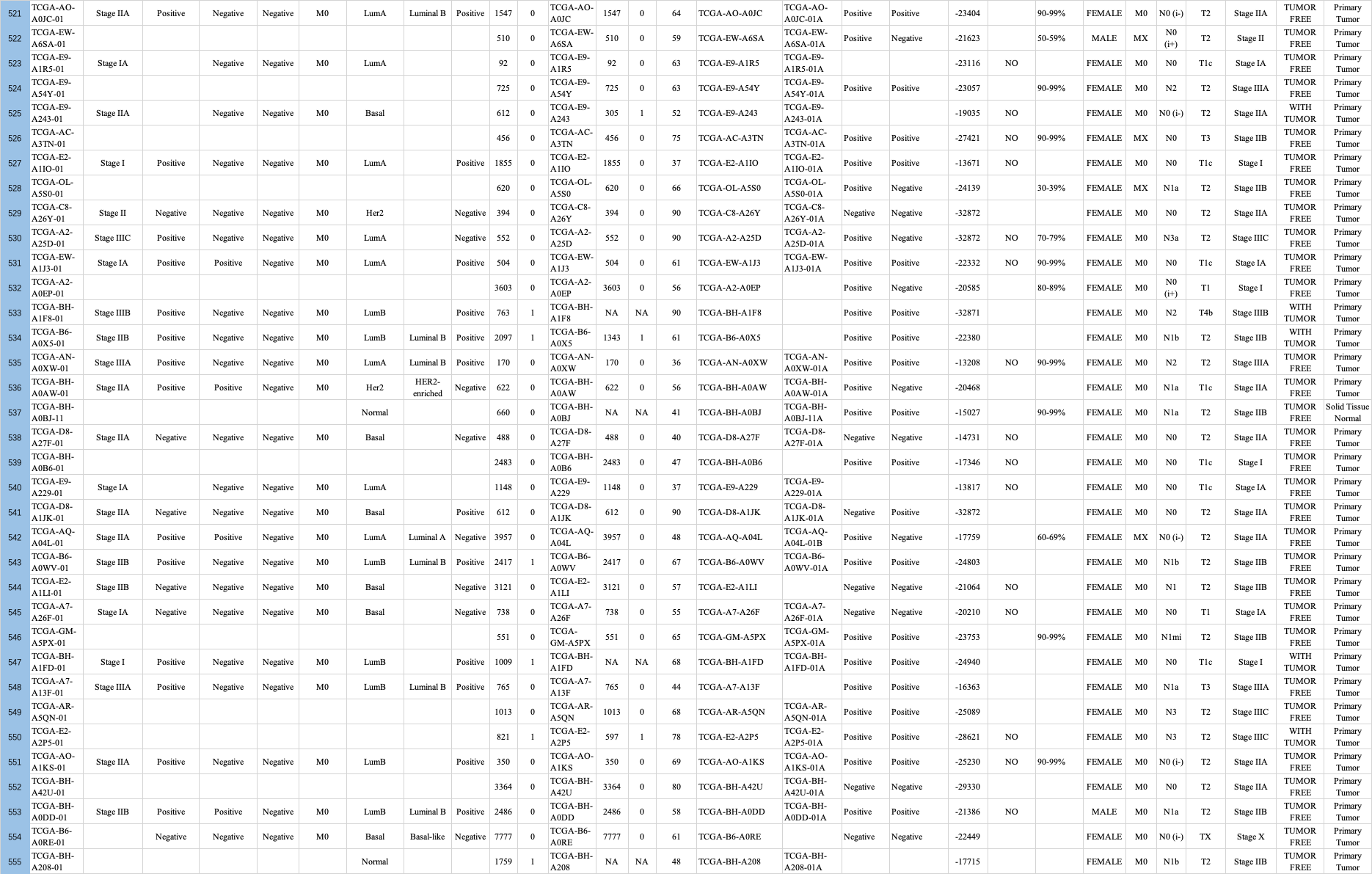


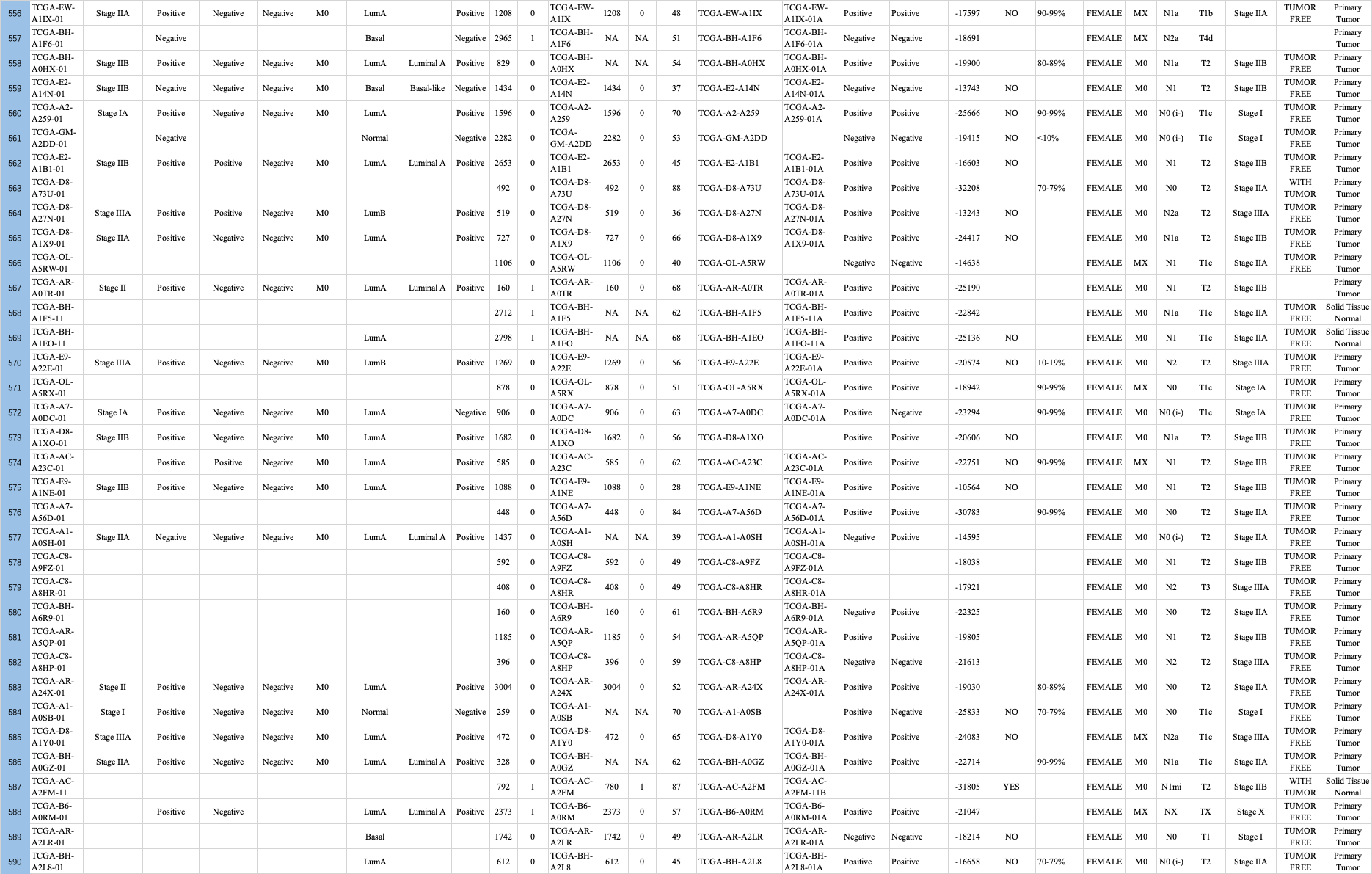


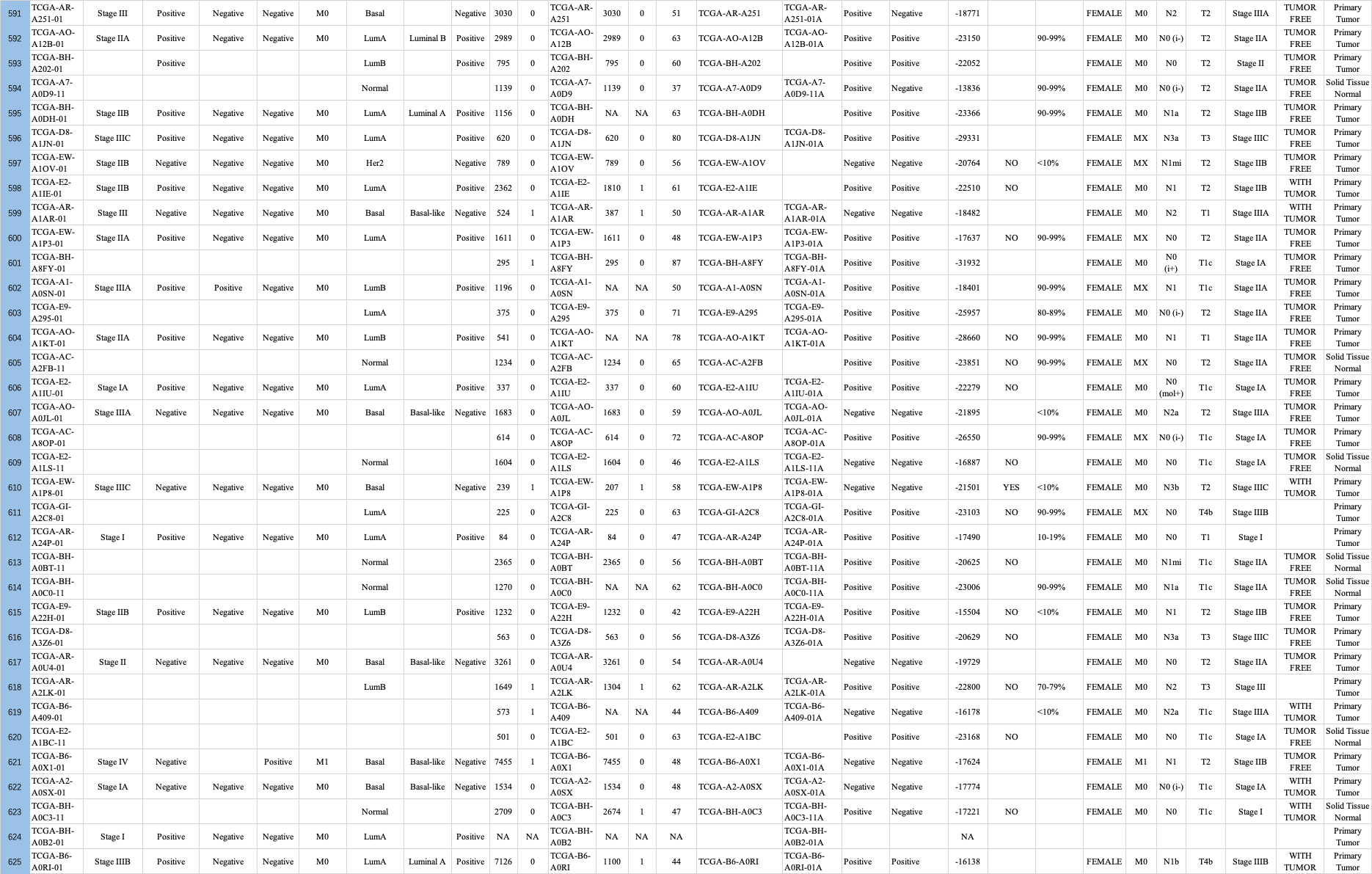


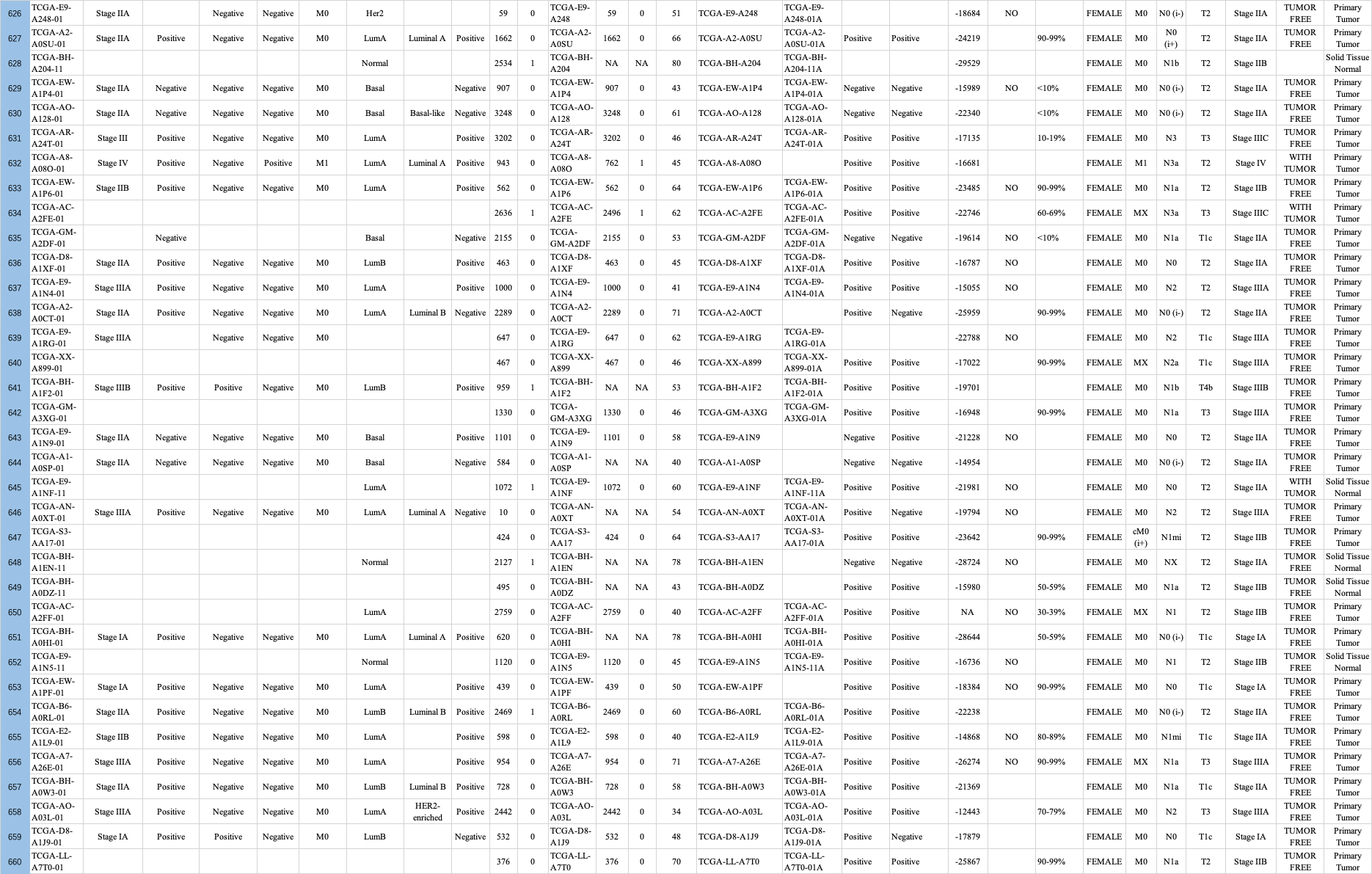


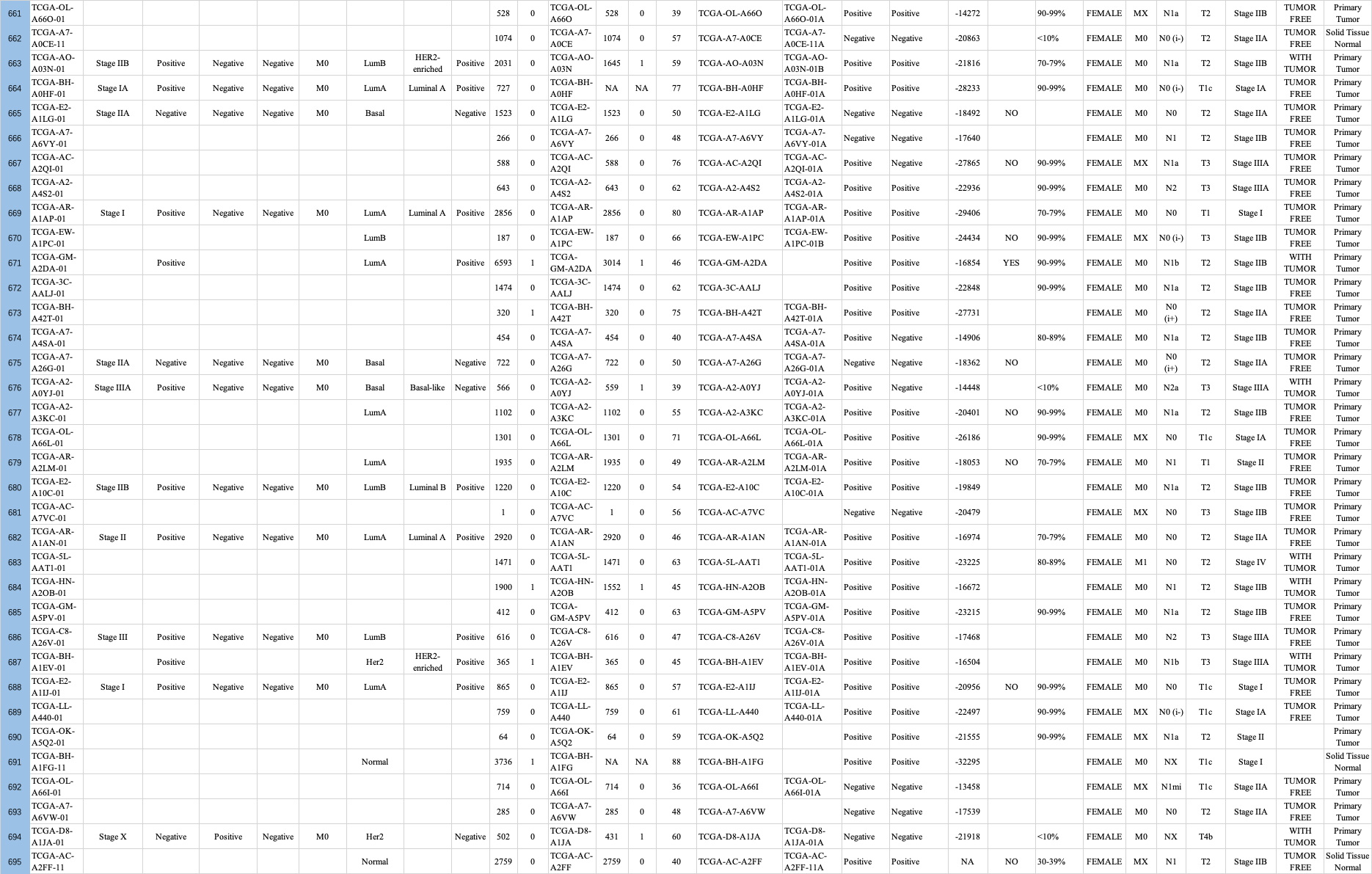


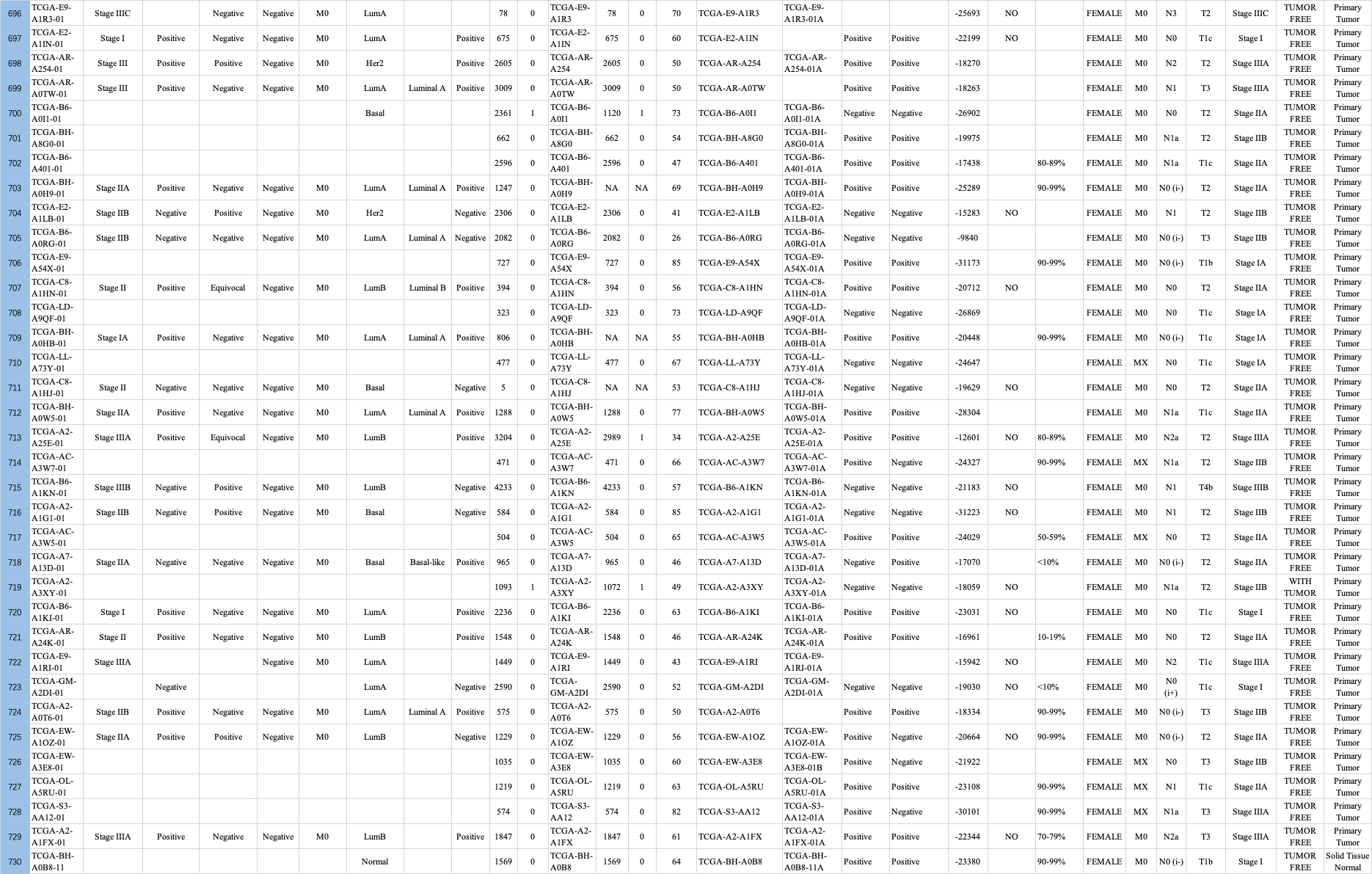


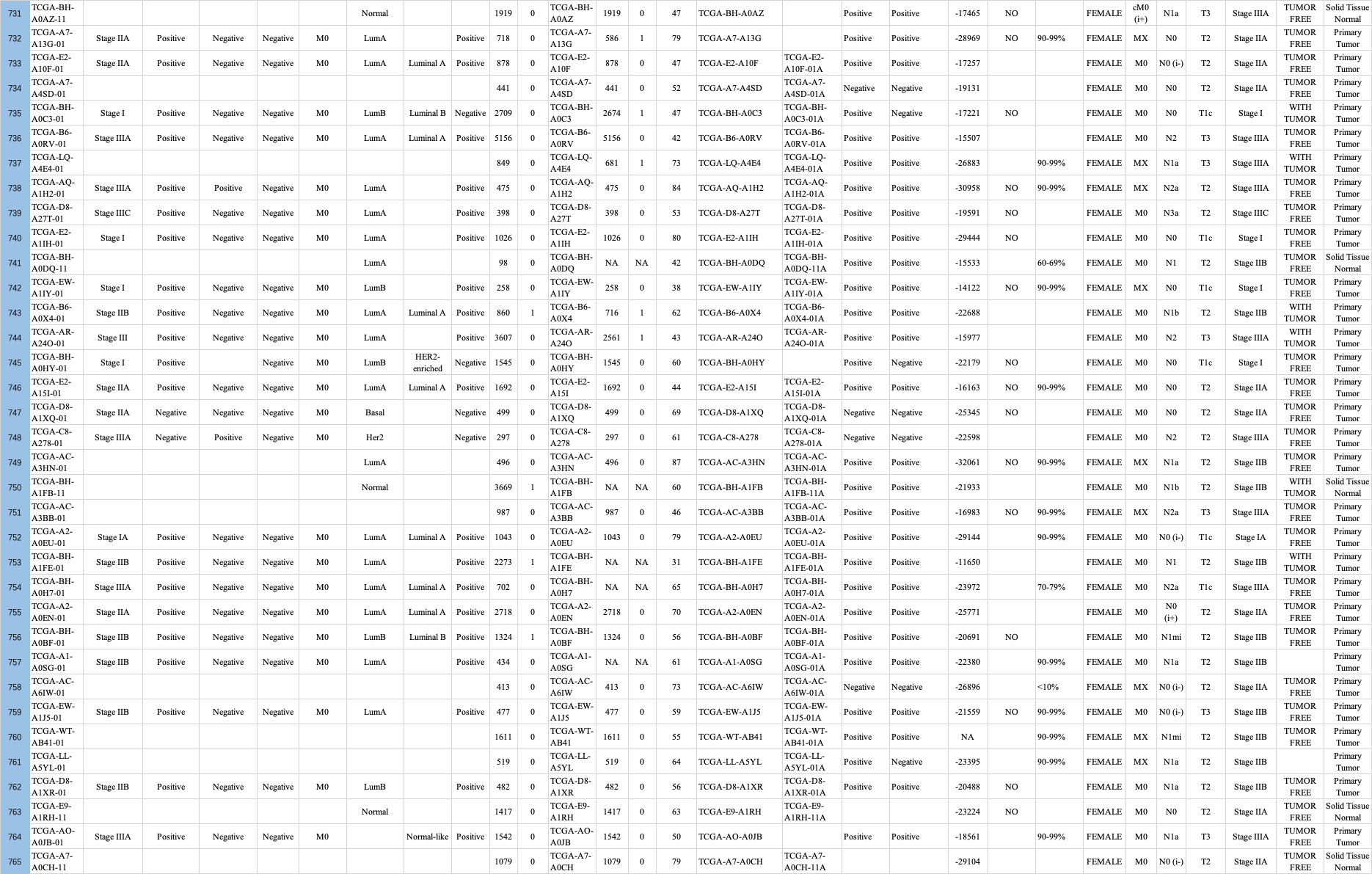


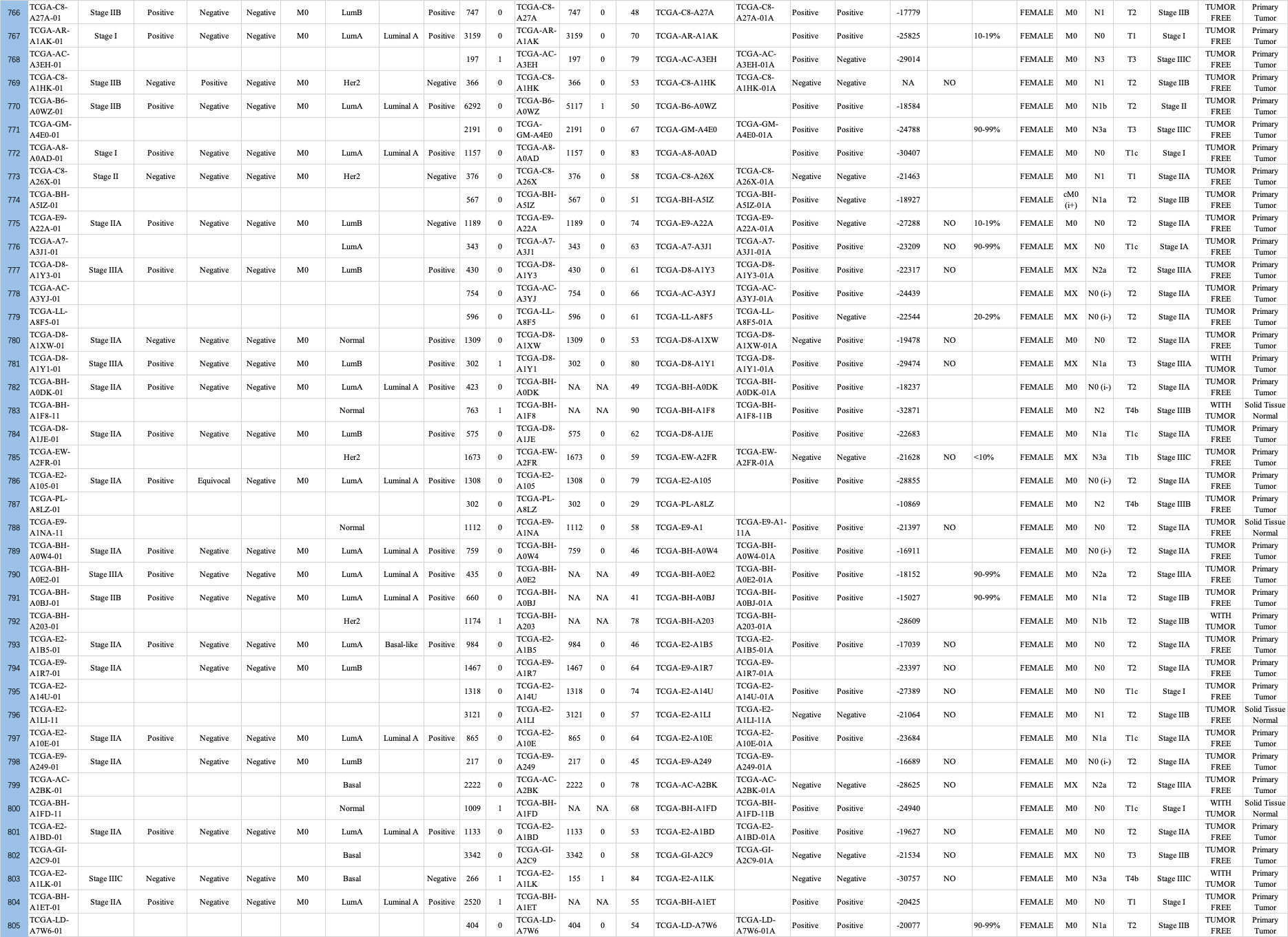


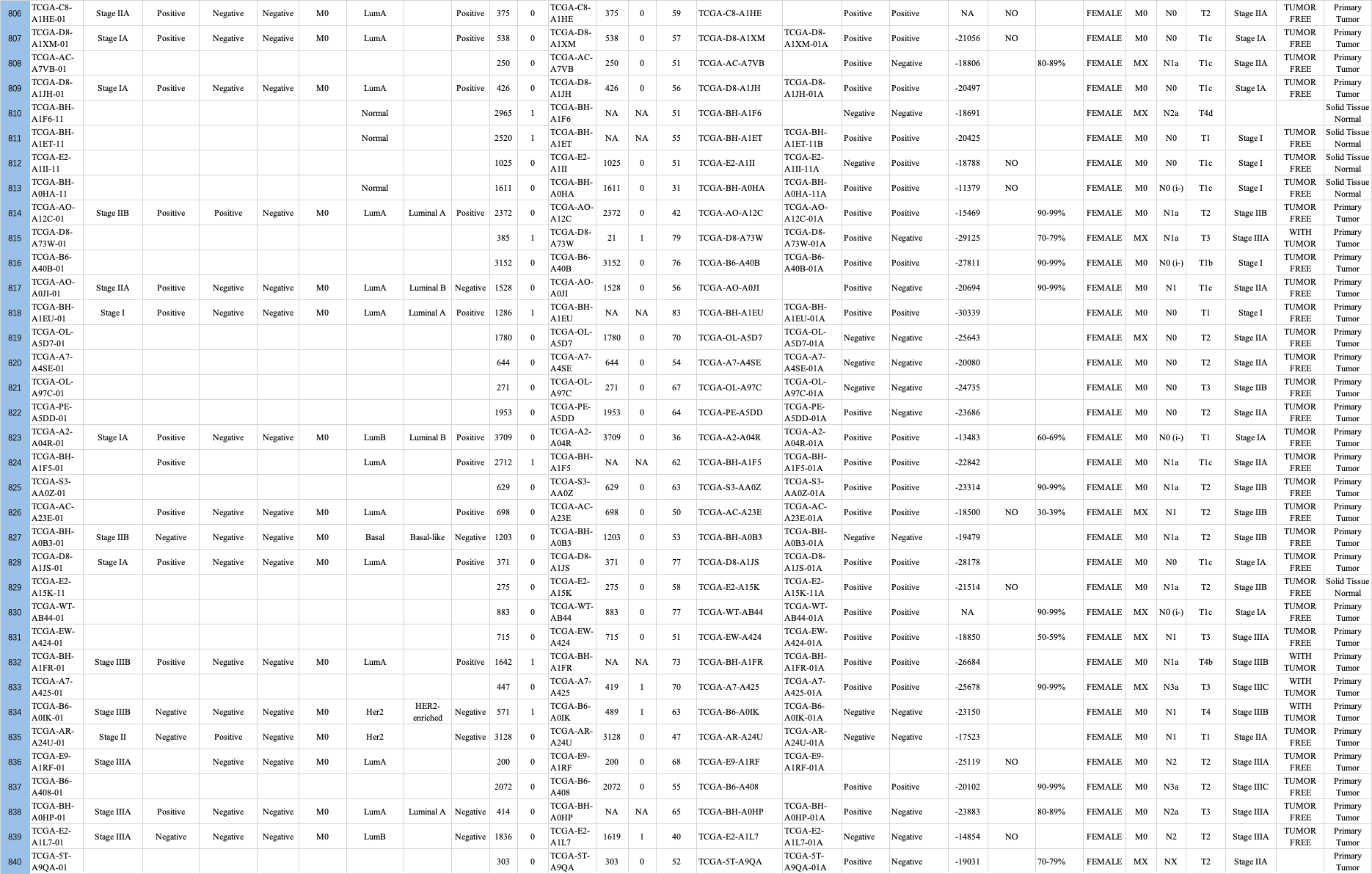


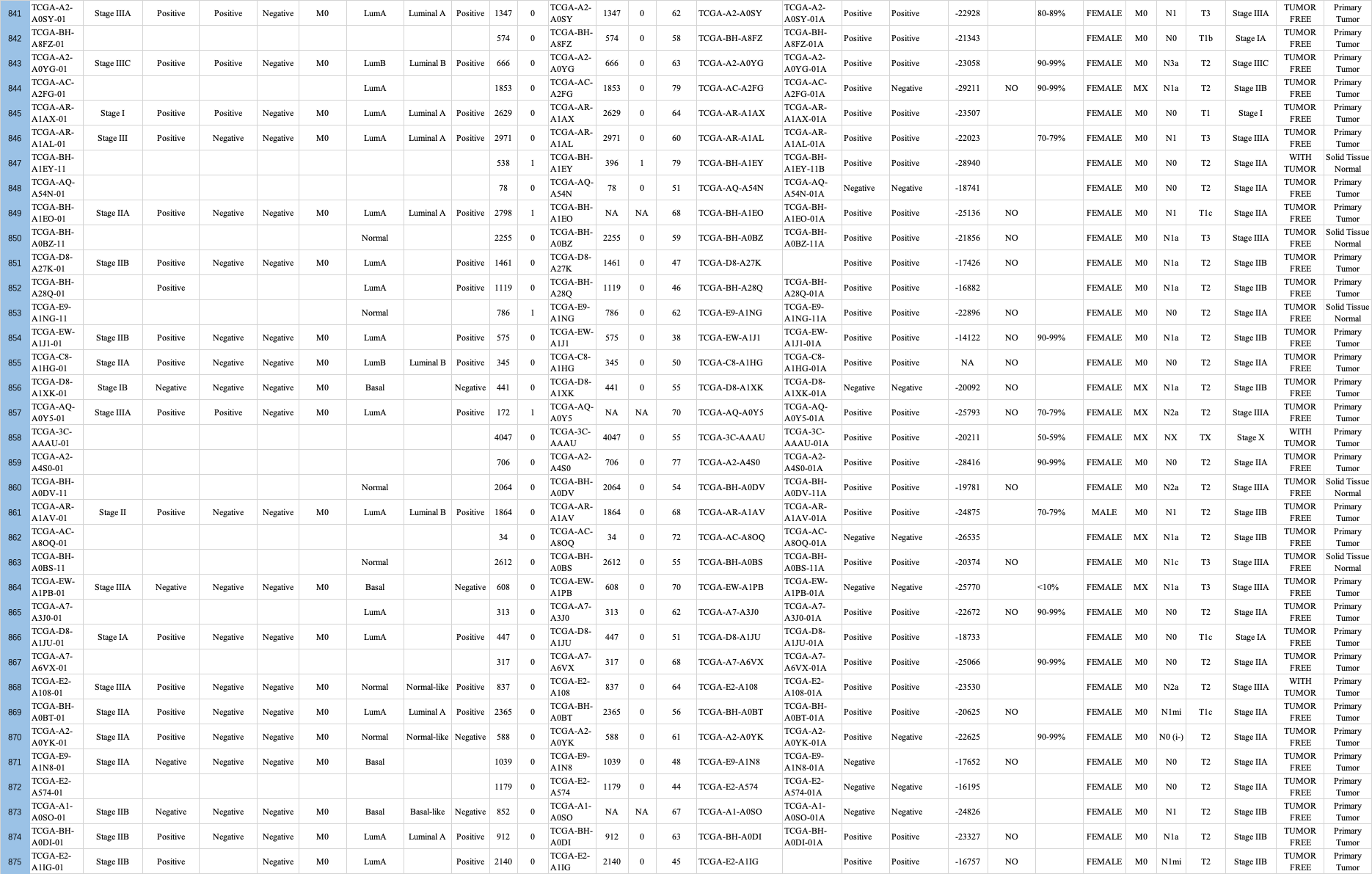


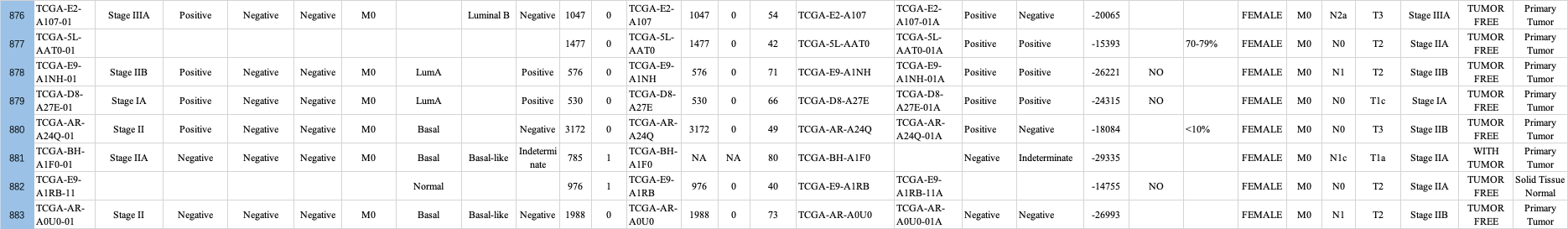


**TABLE S10 Chromosome location of 1332 differentially methylated regions in subtype classification of breast cancer.**

| **Serial ID** | **Chromosome** | **Start** | **End** |
| --- | --- | --- | --- |
|  | chr1 | 530266 | 530290 |
|  | chr1 | 1195714 | 1195758 |
|  | chr1 | 2498261 | 2498363 |
|  | chr1 | 6464425 | 6464453 |
|  | chr1 | 10075669 | 10075835 |
|  | chr1 | 10757632 | 10757695 |
|  | chr1 | 11627627 | 11627693 |
|  | chr1 | 12270862 | 12270890 |
|  | chr1 | 12605331 | 12605400 |
|  | chr1 | 16433802 | 16433875 |
|  | chr1 | 17059218 | 17059259 |
|  | chr1 | 21872496 | 21872545 |
|  | chr1 | 22693391 | 22693549 |
|  | chr1 | 27145882 | 27145902 |
|  | chr1 | 30275074 | 30275129 |
|  | chr1 | 30585954 | 30585988 |
|  | chr1 | 32756703 | 32756779 |
|  | chr1 | 34041003 | 34041098 |
|  | chr1 | 34451151 | 34451166 |
|  | chr1 | 34494410 | 34494433 |
|  | chr1 | 35171156 | 35171231 |
|  | chr1 | 36165508 | 36165873 |
|  | chr1 | 36957540 | 36957584 |
|  | chr1 | 37057972 | 37058017 |
|  | chr1 | 37484988 | 37485012 |
|  | chr1 | 37680130 | 37680156 |
|  | chr1 | 40173107 | 40173170 |
|  | chr1 | 41310976 | 41311098 |
|  | chr1 | 49323115 | 49323252 |
|  | chr1 | 52259497 | 52259577 |
|  | chr1 | 53817230 | 53817274 |
|  | chr1 | 55048112 | 55048243 |
|  | chr1 | 55056009 | 55056060 |
|  | chr1 | 56529197 | 56529306 |
|  | chr1 | 57439686 | 57439755 |
|  | chr1 | 65033952 | 65033991 |
|  | chr1 | 66815018 | 66815246 |
|  | chr1 | 91188579 | 91188798 |
|  | chr1 | 93129563 | 93129587 |
|  | chr1 | 93194353 | 93194494 |
|  | chr1 | 93810222 | 93810275 |
|  | chr1 | 100259561 | 100259577 |
|  | chr1 | 100861668 | 100861731 |
|  | chr1 | 106465411 | 106465482 |
|  | chr1 | 109312995 | 109313024 |
|  | chr1 | 110288597 | 110288710 |
|  | chr1 | 114355792 | 114355871 |
|  | chr1 | 116520044 | 116520137 |
|  | chr1 | 117708162 | 117708251 |
|  | chr1 | 119626780 | 119626798 |
|  | chr1 | 152906945 | 152907041 |
|  | chr1 | 155074650 | 155074682 |
|  | chr1 | 155621105 | 155621160 |
|  | chr1 | 156572369 | 156572455 |
|  | chr1 | 156879069 | 156879098 |
|  | chr1 | 161374138 | 161374169 |
|  | chr1 | 161531682 | 161531883 |
|  | chr1 | 164923923 | 164924041 |
|  | chr1 | 166261065 | 166261105 |
|  | chr1 | 175357616 | 175357712 |
|  | chr1 | 177752343 | 177752416 |
|  | chr1 | 178027665 | 178027861 |
|  | chr1 | 178578104 | 178578301 |
|  | chr1 | 179431486 | 179431578 |
|  | chr1 | 183005293 | 183005527 |
|  | chr1 | 183300484 | 183300500 |
|  | chr1 | 183331102 | 183331144 |
|  | chr1 | 189457176 | 189457381 |
|  | chr1 | 195281080 | 195281171 |
|  | chr1 | 201583416 | 201583428 |
|  | chr1 | 203045621 | 203045662 |
|  | chr1 | 203237258 | 203237287 |
|  | chr1 | 204044813 | 204044904 |
|  | chr1 | 209271289 | 209271472 |
|  | chr1 | 209542269 | 209542440 |
|  | chr1 | 210014223 | 210014252 |
|  | chr1 | 211313097 | 211313161 |
|  | chr1 | 211731443 | 211731487 |
|  | chr1 | 213870561 | 213870776 |
|  | chr1 | 215106880 | 215106902 |
|  | chr1 | 218762328 | 218762369 |
|  | chr1 | 220948750 | 220948799 |
|  | chr1 | 221322047 | 221322180 |
|  | chr1 | 221805542 | 221805572 |
|  | chr1 | 225040704 | 225040724 |
|  | chr1 | 227139743 | 227139818 |
|  | chr1 | 228647638 | 228647735 |
|  | chr1 | 230279567 | 230279757 |
|  | chr1 | 230431918 | 230432080 |
|  | chr1 | 233569743 | 233569811 |
|  | chr1 | 234432434 | 234432594 |
|  | chr1 | 234911510 | 234911545 |
|  | chr1 | 236782940 | 236782958 |
|  | chr1 | 238620592 | 238620824 |
|  | chr1 | 238727179 | 238727399 |
|  | chr1 | 239130811 | 239130981 |
|  | chr1 | 240310276 | 240310357 |
|  | chr1 | 241461893 | 241462097 |
|  | chr1 | 241485165 | 241485253 |
|  | chr1 | 241546060 | 241546102 |
|  | chr1 | 242293973 | 242294020 |
|  | chr1 | 244062741 | 244062788 |
|  | chr1 | 245284978 | 245285027 |
|  | chr1 | 245506050 | 245506120 |
|  | chr2 | 772773 | 772808 |
|  | chr2 | 5636195 | 5636216 |
|  | chr2 | 5766640 | 5766863 |
|  | chr2 | 6055107 | 6055242 |
|  | chr2 | 6567907 | 6568101 |
|  | chr2 | 8199575 | 8199615 |
|  | chr2 | 11784148 | 11784300 |
|  | chr2 | 14494715 | 14494771 |
|  | chr2 | 21023442 | 21023483 |
|  | chr2 | 21213660 | 21213671 |
|  | chr2 | 21229159 | 21229234 |
|  | chr2 | 22225782 | 22226026 |
|  | chr2 | 23509758 | 23509830 |
|  | chr2 | 27192631 | 27192717 |
|  | chr2 | 27736348 | 27736392 |
|  | chr2 | 29591768 | 29591855 |
|  | chr2 | 30334447 | 30334567 |
|  | chr2 | 32038463 | 32038679 |
|  | chr2 | 35146302 | 35146374 |
|  | chr2 | 38924384 | 38924408 |
|  | chr2 | 39367442 | 39367786 |
|  | chr2 | 39846606 | 39846653 |
|  | chr2 | 41081881 | 41082060 |
|  | chr2 | 43371061 | 43371120 |
|  | chr2 | 44390595 | 44390609 |
|  | chr2 | 49129699 | 49129974 |
|  | chr2 | 54240147 | 54240180 |
|  | chr2 | 55365866 | 55365946 |
|  | chr2 | 58948300 | 58948458 |
|  | chr2 | 62380959 | 62381078 |
|  | chr2 | 66308477 | 66308546 |
|  | chr2 | 69399169 | 69399334 |
|  | chr2 | 74206415 | 74206446 |
|  | chr2 | 74488706 | 74488781 |
|  | chr2 | 74612758 | 74612888 |
|  | chr2 | 78247659 | 78247758 |
|  | chr2 | 80646159 | 80646289 |
|  | chr2 | 83018114 | 83018170 |
|  | chr2 | 83130306 | 83130366 |
|  | chr2 | 84955243 | 84955494 |
|  | chr2 | 85442283 | 85442406 |
|  | chr2 | 95701452 | 95701518 |
|  | chr2 | 96833845 | 96833900 |
|  | chr2 | 96884313 | 96884352 |
|  | chr2 | 97723892 | 97723995 |
|  | chr2 | 102959777 | 102959853 |
|  | chr2 | 104001440 | 104001615 |
|  | chr2 | 110859541 | 110859552 |
|  | chr2 | 115050479 | 115050656 |
|  | chr2 | 115509204 | 115509267 |
|  | chr2 | 116735728 | 116736060 |
|  | chr2 | 117356683 | 117356843 |
|  | chr2 | 118130762 | 118130904 |
|  | chr2 | 121301459 | 121301524 |
|  | chr2 | 121739246 | 121739274 |
|  | chr2 | 126126403 | 126126488 |
|  | chr2 | 131040631 | 131040723 |
|  | chr2 | 133622572 | 133622883 |
|  | chr2 | 135052541 | 135052691 |
|  | chr2 | 137751363 | 137751489 |
|  | chr2 | 139954384 | 139954572 |
|  | chr2 | 143904924 | 143904945 |
|  | chr2 | 147614112 | 147614321 |
|  | chr2 | 153346901 | 153346967 |
|  | chr2 | 156866161 | 156866276 |
|  | chr2 | 162102946 | 162102976 |
|  | chr2 | 167325028 | 167325139 |
|  | chr2 | 167815548 | 167815684 |
|  | chr2 | 170627627 | 170627719 |
|  | chr2 | 171258163 | 171258215 |
|  | chr2 | 174740378 | 174740431 |
|  | chr2 | 175002995 | 175003156 |
|  | chr2 | 177015193 | 177015211 |
|  | chr2 | 185988232 | 185988307 |
|  | chr2 | 186131734 | 186131812 |
|  | chr2 | 186569647 | 186569785 |
|  | chr2 | 198144715 | 198144806 |
|  | chr2 | 200470861 | 200470938 |
|  | chr2 | 204212180 | 204212273 |
|  | chr2 | 206543408 | 206543492 |
|  | chr2 | 209272107 | 209272124 |
|  | chr2 | 211488330 | 211488578 |
|  | chr2 | 211839368 | 211839488 |
|  | chr2 | 213969330 | 213969374 |
|  | chr2 | 214823625 | 214823762 |
|  | chr2 | 216533439 | 216533629 |
|  | chr2 | 219198920 | 219199085 |
|  | chr2 | 220318148 | 220318343 |
|  | chr2 | 221966426 | 221966545 |
|  | chr2 | 223824233 | 223824322 |
|  | chr2 | 225818340 | 225818367 |
|  | chr2 | 225833477 | 225833811 |
|  | chr2 | 227993381 | 227993474 |
|  | chr2 | 229056669 | 229056683 |
|  | chr2 | 229184027 | 229184112 |
|  | chr2 | 230161344 | 230161505 |
|  | chr2 | 230430765 | 230430868 |
|  | chr2 | 231571938 | 231571980 |
|  | chr2 | 233247451 | 233247671 |
|  | chr2 | 234388999 | 234389033 |
|  | chr2 | 234699784 | 234699995 |
|  | chr2 | 238630755 | 238630831 |
|  | chr2 | 238832500 | 238832535 |
|  | chr2 | 239567818 | 239567863 |
|  | chr2 | 240697467 | 240697713 |
|  | chr2 | 240743693 | 240743794 |
|  | chr2 | 241504591 | 241504644 |
|  | chr2 | 242618194 | 242618226 |
|  | chr3 | 492283 | 492452 |
|  | chr3 | 4545014 | 4545131 |
|  | chr3 | 6685875 | 6685998 |
|  | chr3 | 7830045 | 7830219 |
|  | chr3 | 11008277 | 11008567 |
|  | chr3 | 12597632 | 12597723 |
|  | chr3 | 13134234 | 13134441 |
|  | chr3 | 13188231 | 13188380 |
|  | chr3 | 19666743 | 19666923 |
|  | chr3 | 20101505 | 20101618 |
|  | chr3 | 20312090 | 20312105 |
|  | chr3 | 20903218 | 20903345 |
|  | chr3 | 29690030 | 29690103 |
|  | chr3 | 31295482 | 31295587 |
|  | chr3 | 32156132 | 32156202 |
|  | chr3 | 34993556 | 34993664 |
|  | chr3 | 46936531 | 46936658 |
|  | chr3 | 49379031 | 49379058 |
|  | chr3 | 55121177 | 55121217 |
|  | chr3 | 55679706 | 55679738 |
|  | chr3 | 59685502 | 59685585 |
|  | chr3 | 61511267 | 61511338 |
|  | chr3 | 63429776 | 63429961 |
|  | chr3 | 64789035 | 64789190 |
|  | chr3 | 68701019 | 68701097 |
|  | chr3 | 70903480 | 70903596 |
|  | chr3 | 71115409 | 71115505 |
|  | chr3 | 71359710 | 71359782 |
|  | chr3 | 73347853 | 73347878 |
|  | chr3 | 75525739 | 75525766 |
|  | chr3 | 75980748 | 75980801 |
|  | chr3 | 76227064 | 76227209 |
|  | chr3 | 77641935 | 77642001 |
|  | chr3 | 78100282 | 78100392 |
|  | chr3 | 88198079 | 88198089 |
|  | chr3 | 99511391 | 99511564 |
|  | chr3 | 101406346 | 101406420 |
|  | chr3 | 107712377 | 107712453 |
|  | chr3 | 108975135 | 108975245 |
|  | chr3 | 109550542 | 109550601 |
|  | chr3 | 112195051 | 112195102 |
|  | chr3 | 113421030 | 113421050 |
|  | chr3 | 115928212 | 115928239 |
|  | chr3 | 128416432 | 128416631 |
|  | chr3 | 136714319 | 136714377 |
|  | chr3 | 137039576 | 137039604 |
|  | chr3 | 145210114 | 145210152 |
|  | chr3 | 145832931 | 145833034 |
|  | chr3 | 146201033 | 146201070 |
|  | chr3 | 147743801 | 147744068 |
|  | chr3 | 150716917 | 150717044 |
|  | chr3 | 152804892 | 152805091 |
|  | chr3 | 162051639 | 162051671 |
|  | chr3 | 163719235 | 163719384 |
|  | chr3 | 164251218 | 164251248 |
|  | chr3 | 164265859 | 164266172 |
|  | chr3 | 164970926 | 164971031 |
|  | chr3 | 169927178 | 169927195 |
|  | chr3 | 172046842 | 172046855 |
|  | chr3 | 173066246 | 173066302 |
|  | chr3 | 173734028 | 173734229 |
|  | chr3 | 177168961 | 177169023 |
|  | chr3 | 180779389 | 180779621 |
|  | chr3 | 182510051 | 182510117 |
|  | chr3 | 182972978 | 182973265 |
|  | chr3 | 185048171 | 185048197 |
|  | chr3 | 187783937 | 187784089 |
|  | chr3 | 189590475 | 189590562 |
|  | chr3 | 191272538 | 191272565 |
|  | chr3 | 195314008 | 195314084 |
|  | chr4 | 3647219 | 3647245 |
|  | chr4 | 5944924 | 5944945 |
|  | chr4 | 6685189 | 6685234 |
|  | chr4 | 9005050 | 9005087 |
|  | chr4 | 9116640 | 9116732 |
|  | chr4 | 9569055 | 9569093 |
|  | chr4 | 12270872 | 12271064 |
|  | chr4 | 12944785 | 12944907 |
|  | chr4 | 15260951 | 15261040 |
|  | chr4 | 18937510 | 18937571 |
|  | chr4 | 21203149 | 21203210 |
|  | chr4 | 22213943 | 22213964 |
|  | chr4 | 27470283 | 27470530 |
|  | chr4 | 30165379 | 30165429 |
|  | chr4 | 30501801 | 30501995 |
|  | chr4 | 31647299 | 31647495 |
|  | chr4 | 35503664 | 35503732 |
|  | chr4 | 35743016 | 35743131 |
|  | chr4 | 37221985 | 37222068 |
|  | chr4 | 39310293 | 39310362 |
|  | chr4 | 40267115 | 40267150 |
|  | chr4 | 45038146 | 45038315 |
|  | chr4 | 45798295 | 45798497 |
|  | chr4 | 47827919 | 47827943 |
|  | chr4 | 49244680 | 49244766 |
|  | chr4 | 49262341 | 49262407 |
|  | chr4 | 56264056 | 56264195 |
|  | chr4 | 57254798 | 57254869 |
|  | chr4 | 58961390 | 58961570 |
|  | chr4 | 61628233 | 61628279 |
|  | chr4 | 62731948 | 62731991 |
|  | chr4 | 63923994 | 63924144 |
|  | chr4 | 68197103 | 68197272 |
|  | chr4 | 69054529 | 69054601 |
|  | chr4 | 71572901 | 71573017 |
|  | chr4 | 71820194 | 71820261 |
|  | chr4 | 78289015 | 78289082 |
|  | chr4 | 81022394 | 81022476 |
|  | chr4 | 84700001 | 84700182 |
|  | chr4 | 89737557 | 89737677 |
|  | chr4 | 92094519 | 92094544 |
|  | chr4 | 94762234 | 94762429 |
|  | chr4 | 100277702 | 100277737 |
|  | chr4 | 100444187 | 100444258 |
|  | chr4 | 109348559 | 109348625 |
|  | chr4 | 109951163 | 109951213 |
|  | chr4 | 115759739 | 115759906 |
|  | chr4 | 117081635 | 117081777 |
|  | chr4 | 128981437 | 128981506 |
|  | chr4 | 133650269 | 133650285 |
|  | chr4 | 134283796 | 134283896 |
|  | chr4 | 139938879 | 139938948 |
|  | chr4 | 140655470 | 140655648 |
|  | chr4 | 142021646 | 142021821 |
|  | chr4 | 146700790 | 146700867 |
|  | chr4 | 152967801 | 152967826 |
|  | chr4 | 158471218 | 158471290 |
|  | chr4 | 162466513 | 162466608 |
|  | chr4 | 163483825 | 163483943 |
|  | chr4 | 165971955 | 165972001 |
|  | chr4 | 173289357 | 173289491 |
|  | chr4 | 175328716 | 175328752 |
|  | chr4 | 178805846 | 178806036 |
|  | chr4 | 180002204 | 180002224 |
|  | chr4 | 180252652 | 180252747 |
|  | chr4 | 181107287 | 181107395 |
|  | chr4 | 183131186 | 183131320 |
|  | chr4 | 183283955 | 183283977 |
|  | chr4 | 185209950 | 185209988 |
|  | chr4 | 187077189 | 187077214 |
|  | chr4 | 189587309 | 189587369 |
|  | chr4 | 189686134 | 189686221 |
|  | chr4 | 190462443 | 190462508 |
|  | chr5 | 1643345 | 1643471 |
|  | chr5 | 2239476 | 2239686 |
|  | chr5 | 2416313 | 2416400 |
|  | chr5 | 4920271 | 4920313 |
|  | chr5 | 10498633 | 10498680 |
|  | chr5 | 11378714 | 11378874 |
|  | chr5 | 11851147 | 11851214 |
|  | chr5 | 12169248 | 12169451 |
|  | chr5 | 13637965 | 13638008 |
|  | chr5 | 14149496 | 14149682 |
|  | chr5 | 15523559 | 15523595 |
|  | chr5 | 16082678 | 16082928 |
|  | chr5 | 16219173 | 16219224 |
|  | chr5 | 17143491 | 17143533 |
|  | chr5 | 17741665 | 17741842 |
|  | chr5 | 29336384 | 29336405 |
|  | chr5 | 29459216 | 29459278 |
|  | chr5 | 33875042 | 33875079 |
|  | chr5 | 36739297 | 36739336 |
|  | chr5 | 38409106 | 38409156 |
|  | chr5 | 40838494 | 40838514 |
|  | chr5 | 40841207 | 40841318 |
|  | chr5 | 42124196 | 42124370 |
|  | chr5 | 45994474 | 45994652 |
|  | chr5 | 51822646 | 51822753 |
|  | chr5 | 53840051 | 53840143 |
|  | chr5 | 55317034 | 55317068 |
|  | chr5 | 55962868 | 55962986 |
|  | chr5 | 61546344 | 61546514 |
|  | chr5 | 62943197 | 62943294 |
|  | chr5 | 76038443 | 76038463 |
|  | chr5 | 80313570 | 80313685 |
|  | chr5 | 85443934 | 85444060 |
|  | chr5 | 87066430 | 87066459 |
|  | chr5 | 102006097 | 102006187 |
|  | chr5 | 106666755 | 106666859 |
|  | chr5 | 109035979 | 109036108 |
|  | chr5 | 112254281 | 112254337 |
|  | chr5 | 112274862 | 112274951 |
|  | chr5 | 115394218 | 115394236 |
|  | chr5 | 121473585 | 121473638 |
|  | chr5 | 126139531 | 126139615 |
|  | chr5 | 134934704 | 134934809 |
|  | chr5 | 135365080 | 135365092 |
|  | chr5 | 135547545 | 135547575 |
|  | chr5 | 141652332 | 141652373 |
|  | chr5 | 143047498 | 143047722 |
|  | chr5 | 147300317 | 147300453 |
|  | chr5 | 148641039 | 148641095 |
|  | chr5 | 149252564 | 149252597 |
|  | chr5 | 151853964 | 151854202 |
|  | chr5 | 152912868 | 152912899 |
|  | chr5 | 154071440 | 154071462 |
|  | chr5 | 154323259 | 154323322 |
|  | chr5 | 159397957 | 159398021 |
|  | chr5 | 159547398 | 159547487 |
|  | chr5 | 163588402 | 163588450 |
|  | chr5 | 163945967 | 163946010 |
|  | chr5 | 164999794 | 164999847 |
|  | chr5 | 168286563 | 168286727 |
|  | chr5 | 171114998 | 171115066 |
|  | chr5 | 171586999 | 171587091 |
|  | chr5 | 173198493 | 173198518 |
|  | chr5 | 173538765 | 173538792 |
|  | chr5 | 176822365 | 176822446 |
|  | chr5 | 178054577 | 178054668 |
|  | chr5 | 180610088 | 180610112 |
|  | chr6 | 996674 | 996858 |
|  | chr6 | 1246539 | 1246631 |
|  | chr6 | 2712920 | 2712957 |
|  | chr6 | 2724649 | 2724707 |
|  | chr6 | 2785528 | 2785677 |
|  | chr6 | 2821412 | 2821615 |
|  | chr6 | 3285115 | 3285283 |
|  | chr6 | 3768626 | 3768660 |
|  | chr6 | 3849027 | 3849193 |
|  | chr6 | 5136190 | 5136218 |
|  | chr6 | 6043200 | 6043394 |
|  | chr6 | 6468440 | 6468450 |
|  | chr6 | 10348237 | 10348342 |
|  | chr6 | 10638513 | 10638551 |
|  | chr6 | 12762254 | 12762331 |
|  | chr6 | 15223505 | 15223581 |
|  | chr6 | 15764327 | 15764362 |
|  | chr6 | 18512918 | 18513124 |
|  | chr6 | 19890432 | 19890515 |
|  | chr6 | 19960113 | 19960144 |
|  | chr6 | 21606649 | 21606681 |
|  | chr6 | 23315970 | 23316121 |
|  | chr6 | 24681013 | 24681266 |
|  | chr6 | 25299042 | 25299110 |
|  | chr6 | 35150667 | 35150683 |
|  | chr6 | 36301033 | 36301071 |
|  | chr6 | 36992413 | 36992507 |
|  | chr6 | 52367849 | 52367896 |
|  | chr6 | 55209103 | 55209292 |
|  | chr6 | 66328762 | 66328929 |
|  | chr6 | 69700200 | 69700291 |
|  | chr6 | 72127281 | 72127317 |
|  | chr6 | 74060884 | 74060952 |
|  | chr6 | 74769523 | 74769774 |
|  | chr6 | 74791150 | 74791198 |
|  | chr6 | 76090983 | 76091041 |
|  | chr6 | 76403885 | 76403923 |
|  | chr6 | 76907918 | 76907993 |
|  | chr6 | 77749113 | 77749365 |
|  | chr6 | 77901819 | 77901962 |
|  | chr6 | 84140029 | 84140070 |
|  | chr6 | 84992659 | 84992828 |
|  | chr6 | 86767656 | 86767688 |
|  | chr6 | 86893906 | 86893996 |
|  | chr6 | 95638130 | 95638154 |
|  | chr6 | 97476043 | 97476091 |
|  | chr6 | 107781498 | 107781523 |
|  | chr6 | 109028665 | 109028793 |
|  | chr6 | 111147954 | 111147983 |
|  | chr6 | 112649972 | 112650048 |
|  | chr6 | 113248118 | 113248397 |
|  | chr6 | 115890865 | 115890937 |
|  | chr6 | 135061303 | 135061434 |
|  | chr6 | 135488371 | 135488407 |
|  | chr6 | 136414317 | 136414648 |
|  | chr6 | 139697692 | 139697783 |
|  | chr6 | 142016222 | 142016337 |
|  | chr6 | 144265688 | 144265871 |
|  | chr6 | 146748394 | 146748505 |
|  | chr6 | 147831374 | 147831459 |
|  | chr6 | 149365650 | 149365694 |
|  | chr6 | 149382267 | 149382301 |
|  | chr6 | 151258496 | 151258551 |
|  | chr6 | 151634210 | 151634399 |
|  | chr6 | 153942390 | 153942448 |
|  | chr6 | 154665736 | 154665770 |
|  | chr6 | 159087552 | 159087598 |
|  | chr6 | 161674308 | 161674350 |
|  | chr6 | 162314408 | 162314593 |
|  | chr7 | 126274 | 126290 |
|  | chr7 | 697412 | 697491 |
|  | chr7 | 866493 | 866527 |
|  | chr7 | 1298844 | 1298858 |
|  | chr7 | 1337419 | 1337482 |
|  | chr7 | 3332970 | 3333021 |
|  | chr7 | 4126170 | 4126217 |
|  | chr7 | 4834459 | 4834559 |
|  | chr7 | 5201064 | 5201200 |
|  | chr7 | 5500578 | 5500642 |
|  | chr7 | 5558971 | 5559018 |
|  | chr7 | 6617523 | 6617559 |
|  | chr7 | 6704235 | 6704261 |
|  | chr7 | 12335609 | 12335692 |
|  | chr7 | 19885970 | 19886119 |
|  | chr7 | 22757395 | 22757512 |
|  | chr7 | 25790534 | 25790640 |
|  | chr7 | 26421332 | 26421390 |
|  | chr7 | 26570602 | 26570642 |
|  | chr7 | 27178860 | 27178883 |
|  | chr7 | 30543224 | 30543264 |
|  | chr7 | 31724325 | 31724458 |
|  | chr7 | 39042093 | 39042245 |
|  | chr7 | 39496684 | 39496753 |
|  | chr7 | 39628460 | 39628649 |
|  | chr7 | 49298951 | 49298985 |
|  | chr7 | 49854410 | 49854529 |
|  | chr7 | 50793592 | 50793660 |
|  | chr7 | 54015681 | 54015957 |
|  | chr7 | 55959746 | 55959771 |
|  | chr7 | 56817035 | 56817280 |
|  | chr7 | 57653731 | 57653793 |
|  | chr7 | 62244615 | 62244691 |
|  | chr7 | 63062624 | 63062722 |
|  | chr7 | 67546209 | 67546409 |
|  | chr7 | 67600797 | 67600864 |
|  | chr7 | 67647002 | 67647025 |
|  | chr7 | 68293867 | 68293898 |
|  | chr7 | 70080097 | 70080160 |
|  | chr7 | 71341207 | 71341384 |
|  | chr7 | 76423097 | 76423170 |
|  | chr7 | 77687645 | 77687714 |
|  | chr7 | 80260158 | 80260230 |
|  | chr7 | 81439702 | 81439845 |
|  | chr7 | 84589081 | 84589195 |
|  | chr7 | 88685731 | 88685993 |
|  | chr7 | 88884516 | 88884590 |
|  | chr7 | 90115275 | 90115358 |
|  | chr7 | 93996386 | 93996405 |
|  | chr7 | 95410216 | 95410342 |
|  | chr7 | 97647189 | 97647239 |
|  | chr7 | 98209934 | 98210100 |
|  | chr7 | 99077503 | 99077537 |
|  | chr7 | 99778461 | 99778568 |
|  | chr7 | 100359782 | 100359815 |
|  | chr7 | 107286880 | 107287023 |
|  | chr7 | 108539827 | 108539883 |
|  | chr7 | 108805804 | 108805862 |
|  | chr7 | 114571848 | 114571917 |
|  | chr7 | 117759706 | 117759819 |
|  | chr7 | 125165409 | 125165455 |
|  | chr7 | 126459350 | 126459360 |
|  | chr7 | 126691392 | 126691552 |
|  | chr7 | 126710883 | 126711112 |
|  | chr7 | 135443039 | 135443061 |
|  | chr7 | 135913958 | 135914028 |
|  | chr7 | 138560748 | 138560799 |
|  | chr7 | 142902864 | 142902910 |
|  | chr7 | 145946092 | 145946269 |
|  | chr7 | 146442834 | 146443169 |
|  | chr7 | 154410877 | 154410938 |
|  | chr7 | 155324026 | 155324132 |
|  | chr7 | 157100220 | 157100246 |
|  | chr7 | 157641162 | 157641239 |
|  | chr7 | 158909535 | 158909601 |
|  | chr8 | 1873166 | 1873196 |
|  | chr8 | 2744513 | 2744657 |
|  | chr8 | 2750292 | 2750459 |
|  | chr8 | 3794142 | 3794480 |
|  | chr8 | 3836031 | 3836152 |
|  | chr8 | 5520136 | 5520213 |
|  | chr8 | 6697762 | 6697917 |
|  | chr8 | 8241970 | 8242111 |
|  | chr8 | 8377815 | 8377968 |
|  | chr8 | 8654453 | 8654495 |
|  | chr8 | 9225687 | 9225742 |
|  | chr8 | 9339329 | 9339349 |
|  | chr8 | 12342751 | 12342872 |
|  | chr8 | 12383679 | 12383723 |
|  | chr8 | 13979709 | 13979757 |
|  | chr8 | 14401215 | 14401623 |
|  | chr8 | 15957980 | 15958056 |
|  | chr8 | 17003183 | 17003202 |
|  | chr8 | 18218117 | 18218172 |
|  | chr8 | 19385695 | 19385765 |
|  | chr8 | 20685795 | 20685859 |
|  | chr8 | 24881441 | 24881632 |
|  | chr8 | 25485244 | 25485337 |
|  | chr8 | 36957376 | 36957425 |
|  | chr8 | 39328352 | 39328436 |
|  | chr8 | 41287589 | 41287716 |
|  | chr8 | 43315140 | 43315186 |
|  | chr8 | 55751916 | 55752038 |
|  | chr8 | 62263339 | 62263443 |
|  | chr8 | 66548732 | 66548780 |
|  | chr8 | 68537038 | 68537048 |
|  | chr8 | 70839805 | 70839832 |
|  | chr8 | 73246964 | 73247119 |
|  | chr8 | 77737661 | 77737694 |
|  | chr8 | 77797967 | 77798059 |
|  | chr8 | 84051808 | 84051824 |
|  | chr8 | 84577090 | 84577266 |
|  | chr8 | 89868334 | 89868470 |
|  | chr8 | 93382381 | 93382418 |
|  | chr8 | 96814931 | 96814961 |
|  | chr8 | 97416653 | 97416720 |
|  | chr8 | 102042142 | 102042195 |
|  | chr8 | 111690125 | 111690311 |
|  | chr8 | 111884107 | 111884168 |
|  | chr8 | 112983022 | 112983120 |
|  | chr8 | 113854646 | 113854696 |
|  | chr8 | 114824348 | 114824463 |
|  | chr8 | 118108318 | 118108441 |
|  | chr8 | 120693533 | 120693691 |
|  | chr8 | 125530502 | 125530595 |
|  | chr8 | 126245259 | 126245328 |
|  | chr8 | 138531444 | 138531602 |
|  | chr8 | 138694430 | 138694514 |
|  | chr8 | 141111018 | 141111083 |
|  | chr8 | 141431753 | 141431851 |
|  | chr8 | 141629480 | 141629545 |
|  | chr8 | 142672039 | 142672070 |
|  | chr8 | 142889667 | 142889749 |
|  | chr8 | 144136397 | 144136456 |
|  | chr9 | 273044 | 273080 |
|  | chr9 | 2021993 | 2022039 |
|  | chr9 | 3840507 | 3840555 |
|  | chr9 | 7691473 | 7691628 |
|  | chr9 | 9413359 | 9413396 |
|  | chr9 | 10264748 | 10264843 |
|  | chr9 | 11397710 | 11397764 |
|  | chr9 | 11956715 | 11956929 |
|  | chr9 | 12159148 | 12159263 |
|  | chr9 | 14318963 | 14319168 |
|  | chr9 | 16071087 | 16071270 |
|  | chr9 | 23816604 | 23816684 |
|  | chr9 | 24641430 | 24641479 |
|  | chr9 | 33013779 | 33013799 |
|  | chr9 | 34518494 | 34518555 |
|  | chr9 | 35644857 | 35644927 |
|  | chr9 | 37535135 | 37535196 |
|  | chr9 | 39058430 | 39058450 |
|  | chr9 | 44107708 | 44107772 |
|  | chr9 | 45350324 | 45350416 |
|  | chr9 | 74523302 | 74523567 |
|  | chr9 | 74670673 | 74670804 |
|  | chr9 | 77164721 | 77164750 |
|  | chr9 | 77477102 | 77477255 |
|  | chr9 | 79261416 | 79261469 |
|  | chr9 | 79281623 | 79281651 |
|  | chr9 | 80462105 | 80462215 |
|  | chr9 | 81547798 | 81547921 |
|  | chr9 | 81633553 | 81633665 |
|  | chr9 | 83274977 | 83275017 |
|  | chr9 | 89414699 | 89414711 |
|  | chr9 | 90723785 | 90723935 |
|  | chr9 | 93375438 | 93375499 |
|  | chr9 | 98549613 | 98549673 |
|  | chr9 | 101557820 | 101557958 |
|  | chr9 | 102358311 | 102358410 |
|  | chr9 | 104441747 | 104441762 |
|  | chr9 | 105522992 | 105523202 |
|  | chr9 | 107527163 | 107527283 |
|  | chr9 | 107783170 | 107783297 |
|  | chr9 | 111605374 | 111605554 |
|  | chr9 | 115594327 | 115594364 |
|  | chr9 | 118622326 | 118622580 |
|  | chr9 | 121247872 | 121247963 |
|  | chr9 | 125765158 | 125765355 |
|  | chr9 | 129126564 | 129126590 |
|  | chr9 | 129278842 | 129278863 |
|  | chr9 | 130557830 | 130557874 |
|  | chr9 | 131853585 | 131853614 |
|  | chr9 | 135424302 | 135424350 |
|  | chr9 | 135625233 | 135625253 |
|  | chr9 | 137585058 | 137585097 |
|  | chr9 | 138855581 | 138855668 |
|  | chr9 | 139610610 | 139610701 |
|  | chr10 | 1699889 | 1699914 |
|  | chr10 | 3513969 | 3514020 |
|  | chr10 | 3930163 | 3930213 |
|  | chr10 | 5576761 | 5576828 |
|  | chr10 | 7507721 | 7507863 |
|  | chr10 | 7732774 | 7732853 |
|  | chr10 | 10558497 | 10558764 |
|  | chr10 | 10577588 | 10577633 |
|  | chr10 | 11767507 | 11767546 |
|  | chr10 | 12802624 | 12802731 |
|  | chr10 | 13451641 | 13451765 |
|  | chr10 | 14802286 | 14802347 |
|  | chr10 | 16400447 | 16400638 |
|  | chr10 | 18629915 | 18629948 |
|  | chr10 | 22418795 | 22418833 |
|  | chr10 | 22635152 | 22635250 |
|  | chr10 | 24808535 | 24808557 |
|  | chr10 | 26422606 | 26422770 |
|  | chr10 | 26534902 | 26534945 |
|  | chr10 | 28258032 | 28258100 |
|  | chr10 | 29906014 | 29906063 |
|  | chr10 | 34512108 | 34512238 |
|  | chr10 | 36867641 | 36867747 |
|  | chr10 | 38713314 | 38713343 |
|  | chr10 | 42635614 | 42635700 |
|  | chr10 | 42989479 | 42989512 |
|  | chr10 | 43770866 | 43770888 |
|  | chr10 | 44853269 | 44853431 |
|  | chr10 | 54007802 | 54007960 |
|  | chr10 | 54821614 | 54821702 |
|  | chr10 | 60375101 | 60375122 |
|  | chr10 | 61649939 | 61650093 |
|  | chr10 | 62882060 | 62882102 |
|  | chr10 | 65799215 | 65799226 |
|  | chr10 | 73236858 | 73236989 |
|  | chr10 | 74188755 | 74188805 |
|  | chr10 | 74459212 | 74459267 |
|  | chr10 | 78794927 | 78795045 |
|  | chr10 | 84134067 | 84134272 |
|  | chr10 | 85779393 | 85779439 |
|  | chr10 | 85970573 | 85970650 |
|  | chr10 | 93662526 | 93662571 |
|  | chr10 | 94354842 | 94354867 |
|  | chr10 | 95024801 | 95024827 |
|  | chr10 | 96304593 | 96304657 |
|  | chr10 | 98869979 | 98870158 |
|  | chr10 | 98899939 | 98899973 |
|  | chr10 | 99323740 | 99323839 |
|  | chr10 | 102027951 | 102027980 |
|  | chr10 | 103883306 | 103883326 |
|  | chr10 | 104451124 | 104451341 |
|  | chr10 | 107266185 | 107266219 |
|  | chr10 | 108747261 | 108747335 |
|  | chr10 | 109784375 | 109784558 |
|  | chr10 | 111428182 | 111428441 |
|  | chr10 | 111467085 | 111467324 |
|  | chr10 | 111843221 | 111843271 |
|  | chr10 | 112013106 | 112013276 |
|  | chr10 | 112208556 | 112208631 |
|  | chr10 | 113576281 | 113576380 |
|  | chr10 | 116231797 | 116231833 |
|  | chr10 | 119209709 | 119209913 |
|  | chr10 | 119496193 | 119496225 |
|  | chr10 | 120868258 | 120868294 |
|  | chr10 | 123598592 | 123598650 |
|  | chr10 | 124251740 | 124251827 |
|  | chr10 | 129112146 | 129112247 |
|  | chr10 | 129530165 | 129530301 |
|  | chr10 | 129979278 | 129979378 |
|  | chr10 | 130178743 | 130178795 |
|  | chr10 | 130544845 | 130544882 |
|  | chr10 | 130682904 | 130682965 |
|  | chr10 | 132436250 | 132436378 |
|  | chr10 | 132904043 | 132904158 |
|  | chr10 | 134448226 | 134448253 |
|  | chr11 | 469880 | 469983 |
|  | chr11 | 1376275 | 1376291 |
|  | chr11 | 1699972 | 1700053 |
|  | chr11 | 4070620 | 4070649 |
|  | chr11 | 5914934 | 5915119 |
|  | chr11 | 7040164 | 7040191 |
|  | chr11 | 7981532 | 7981599 |
|  | chr11 | 9026477 | 9026536 |
|  | chr11 | 9831121 | 9831197 |
|  | chr11 | 10408871 | 10408959 |
|  | chr11 | 13348396 | 13348462 |
|  | chr11 | 18853263 | 18853339 |
|  | chr11 | 19401165 | 19401204 |
|  | chr11 | 23434984 | 23435183 |
|  | chr11 | 24837487 | 24837737 |
|  | chr11 | 31280608 | 31280638 |
|  | chr11 | 32101243 | 32101338 |
|  | chr11 | 33612630 | 33612758 |
|  | chr11 | 33954221 | 33954284 |
|  | chr11 | 38361051 | 38361264 |
|  | chr11 | 41458967 | 41458977 |
|  | chr11 | 47990992 | 47991055 |
|  | chr11 | 48815232 | 48815401 |
|  | chr11 | 49102489 | 49102604 |
|  | chr11 | 49727470 | 49727569 |
|  | chr11 | 51586159 | 51586172 |
|  | chr11 | 54965178 | 54965394 |
|  | chr11 | 59437958 | 59438043 |
|  | chr11 | 61104457 | 61104538 |
|  | chr11 | 68166023 | 68166043 |
|  | chr11 | 69281671 | 69281735 |
|  | chr11 | 71096315 | 71096347 |
|  | chr11 | 74509570 | 74509605 |
|  | chr11 | 75588307 | 75588372 |
|  | chr11 | 77971325 | 77971456 |
|  | chr11 | 78021752 | 78021783 |
|  | chr11 | 79197215 | 79197312 |
|  | chr11 | 79610623 | 79610737 |
|  | chr11 | 79898503 | 79898538 |
|  | chr11 | 81713898 | 81714059 |
|  | chr11 | 82756136 | 82756274 |
|  | chr11 | 83519070 | 83519130 |
|  | chr11 | 89969541 | 89969576 |
|  | chr11 | 92637617 | 92637672 |
|  | chr11 | 94307935 | 94308000 |
|  | chr11 | 97815361 | 97815482 |
|  | chr11 | 99551252 | 99551489 |
|  | chr11 | 102980775 | 102980795 |
|  | chr11 | 106521037 | 106521192 |
|  | chr11 | 107288478 | 107288521 |
|  | chr11 | 108905829 | 108905942 |
|  | chr11 | 113937189 | 113937299 |
|  | chr11 | 121064512 | 121064750 |
|  | chr11 | 124351457 | 124351474 |
|  | chr11 | 125942470 | 125942707 |
|  | chr11 | 129237889 | 129238086 |
|  | chr11 | 131923827 | 131923984 |
|  | chr11 | 132949639 | 132949784 |
|  | chr11 | 132962391 | 132962572 |
|  | chr11 | 133421753 | 133421887 |
|  | chr11 | 133646845 | 133647021 |
|  | chr12 | 2027242 | 2027356 |
|  | chr12 | 2482250 | 2482301 |
|  | chr12 | 5515564 | 5515606 |
|  | chr12 | 12120478 | 12120574 |
|  | chr12 | 13253206 | 13253239 |
|  | chr12 | 13573496 | 13573569 |
|  | chr12 | 15408623 | 15408726 |
|  | chr12 | 18408358 | 18408535 |
|  | chr12 | 21601215 | 21601247 |
|  | chr12 | 24399993 | 24400079 |
|  | chr12 | 29757096 | 29757208 |
|  | chr12 | 42856414 | 42856465 |
|  | chr12 | 42873072 | 42873143 |
|  | chr12 | 45460077 | 45460130 |
|  | chr12 | 47462432 | 47462483 |
|  | chr12 | 48496571 | 48496591 |
|  | chr12 | 48806600 | 48806688 |
|  | chr12 | 49716238 | 49716274 |
|  | chr12 | 51034598 | 51034671 |
|  | chr12 | 51941221 | 51941448 |
|  | chr12 | 56576264 | 56576300 |
|  | chr12 | 64017552 | 64017706 |
|  | chr12 | 67669200 | 67669250 |
|  | chr12 | 68135193 | 68135371 |
|  | chr12 | 69010020 | 69010153 |
|  | chr12 | 73054015 | 73054054 |
|  | chr12 | 73219551 | 73219609 |
|  | chr12 | 74760058 | 74760194 |
|  | chr12 | 77456738 | 77456841 |
|  | chr12 | 87999743 | 87999859 |
|  | chr12 | 94276458 | 94276519 |
|  | chr12 | 96360232 | 96360288 |
|  | chr12 | 97558905 | 97558940 |
|  | chr12 | 98523507 | 98523659 |
|  | chr12 | 98896359 | 98896416 |
|  | chr12 | 103201081 | 103201298 |
|  | chr12 | 104575997 | 104576221 |
|  | chr12 | 104607536 | 104607583 |
|  | chr12 | 111592299 | 111592470 |
|  | chr12 | 113854372 | 113854592 |
|  | chr12 | 113856241 | 113856272 |
|  | chr12 | 114101495 | 114101527 |
|  | chr12 | 115114647 | 115114762 |
|  | chr12 | 117448018 | 117448081 |
|  | chr12 | 117628566 | 117628592 |
|  | chr12 | 118085565 | 118085644 |
|  | chr12 | 119313322 | 119313385 |
|  | chr12 | 119591006 | 119591095 |
|  | chr12 | 119667850 | 119667917 |
|  | chr12 | 125731012 | 125731029 |
|  | chr12 | 127615499 | 127615538 |
|  | chr12 | 128233283 | 128233369 |
|  | chr12 | 128933269 | 128933351 |
|  | chr12 | 131245857 | 131245916 |
|  | chr12 | 131463413 | 131463473 |
|  | chr12 | 133695850 | 133695895 |
|  | chr13 | 19421093 | 19421384 |
|  | chr13 | 19509160 | 19509361 |
|  | chr13 | 21801645 | 21801723 |
|  | chr13 | 23379100 | 23379276 |
|  | chr13 | 24821395 | 24821461 |
|  | chr13 | 26692145 | 26692168 |
|  | chr13 | 28477851 | 28477968 |
|  | chr13 | 28607075 | 28607113 |
|  | chr13 | 29329760 | 29329834 |
|  | chr13 | 30528637 | 30528660 |
|  | chr13 | 30982970 | 30983009 |
|  | chr13 | 31145448 | 31145495 |
|  | chr13 | 34291724 | 34291792 |
|  | chr13 | 38436959 | 38437013 |
|  | chr13 | 39198869 | 39198970 |
|  | chr13 | 47094998 | 47095026 |
|  | chr13 | 49199828 | 49199859 |
|  | chr13 | 50194321 | 50194491 |
|  | chr13 | 50939995 | 50940179 |
|  | chr13 | 51632668 | 51633013 |
|  | chr13 | 53452056 | 53452092 |
|  | chr13 | 64816906 | 64816950 |
|  | chr13 | 78309415 | 78309609 |
|  | chr13 | 82298558 | 82298692 |
|  | chr13 | 82300822 | 82301087 |
|  | chr13 | 89357240 | 89357317 |
|  | chr13 | 89766085 | 89766322 |
|  | chr13 | 93212015 | 93212115 |
|  | chr13 | 94253094 | 94253200 |
|  | chr13 | 103065600 | 103065770 |
|  | chr13 | 103735851 | 103735883 |
|  | chr13 | 108175766 | 108175972 |
|  | chr13 | 110013010 | 110013078 |
|  | chr13 | 111280996 | 111281033 |
|  | chr13 | 112182010 | 112182048 |
|  | chr13 | 113578543 | 113578606 |
|  | chr13 | 114184461 | 114184512 |
|  | chr13 | 114567447 | 114567465 |
|  | chr13 | 114568402 | 114568448 |
|  | chr14 | 21483524 | 21483609 |
|  | chr14 | 21750167 | 21750188 |
|  | chr14 | 22118531 | 22118693 |
|  | chr14 | 23939717 | 23939742 |
|  | chr14 | 26885137 | 26885247 |
|  | chr14 | 27387612 | 27387807 |
|  | chr14 | 29668626 | 29668821 |
|  | chr14 | 30374888 | 30375008 |
|  | chr14 | 33756648 | 33756711 |
|  | chr14 | 34989244 | 34989315 |
|  | chr14 | 41303339 | 41303520 |
|  | chr14 | 41313320 | 41313379 |
|  | chr14 | 41575859 | 41576124 |
|  | chr14 | 42438079 | 42438111 |
|  | chr14 | 44921080 | 44921221 |
|  | chr14 | 45542507 | 45542627 |
|  | chr14 | 48264169 | 48264202 |
|  | chr14 | 50692299 | 50692380 |
|  | chr14 | 53358909 | 53359159 |
|  | chr14 | 54639375 | 54639553 |
|  | chr14 | 61950817 | 61950869 |
|  | chr14 | 66404005 | 66404201 |
|  | chr14 | 66470335 | 66470394 |
|  | chr14 | 69906748 | 69906799 |
|  | chr14 | 70460705 | 70460757 |
|  | chr14 | 72580942 | 72581015 |
|  | chr14 | 74796720 | 74796875 |
|  | chr14 | 77691388 | 77691486 |
|  | chr14 | 78081261 | 78081273 |
|  | chr14 | 78100498 | 78100586 |
|  | chr14 | 79210766 | 79210993 |
|  | chr14 | 79301478 | 79301548 |
|  | chr14 | 79478039 | 79478120 |
|  | chr14 | 84948458 | 84948663 |
|  | chr14 | 86640347 | 86640362 |
|  | chr14 | 88473037 | 88473124 |
|  | chr14 | 89413530 | 89413551 |
|  | chr14 | 89577404 | 89577526 |
|  | chr14 | 91263114 | 91263268 |
|  | chr14 | 93909483 | 93909529 |
|  | chr14 | 95357886 | 95358077 |
|  | chr14 | 96434596 | 96434796 |
|  | chr14 | 96489471 | 96489706 |
|  | chr14 | 97624346 | 97624386 |
|  | chr14 | 97713205 | 97713245 |
|  | chr14 | 98764186 | 98764212 |
|  | chr14 | 99503110 | 99503146 |
|  | chr14 | 100215422 | 100215675 |
|  | chr14 | 101817254 | 101817302 |
|  | chr14 | 102071546 | 102071574 |
|  | chr14 | 102290434 | 102290506 |
|  | chr14 | 102776889 | 102776932 |
|  | chr14 | 102784213 | 102784262 |
|  | chr14 | 104416486 | 104416532 |
|  | chr14 | 104707154 | 104707288 |
|  | chr14 | 105748314 | 105748374 |
|  | chr15 | 22550642 | 22550703 |
|  | chr15 | 24312897 | 24313178 |
|  | chr15 | 24927142 | 24927178 |
|  | chr15 | 25379250 | 25379450 |
|  | chr15 | 25550477 | 25550622 |
|  | chr15 | 29958104 | 29958153 |
|  | chr15 | 30112275 | 30112413 |
|  | chr15 | 31528160 | 31528281 |
|  | chr15 | 32099604 | 32099811 |
|  | chr15 | 32109284 | 32109384 |
|  | chr15 | 36585344 | 36585443 |
|  | chr15 | 40693398 | 40693415 |
|  | chr15 | 44036347 | 44036374 |
|  | chr15 | 45678971 | 45679070 |
|  | chr15 | 46075296 | 46075409 |
|  | chr15 | 50543751 | 50543894 |
|  | chr15 | 53094763 | 53094798 |
|  | chr15 | 55144985 | 55145049 |
|  | chr15 | 57060226 | 57060316 |
|  | chr15 | 57939473 | 57939610 |
|  | chr15 | 58000305 | 58000498 |
|  | chr15 | 58552388 | 58552563 |
|  | chr15 | 59937836 | 59937931 |
|  | chr15 | 61752626 | 61752844 |
|  | chr15 | 63538100 | 63538177 |
|  | chr15 | 70006885 | 70006908 |
|  | chr15 | 71335213 | 71335274 |
|  | chr15 | 74436187 | 74436238 |
|  | chr15 | 74548615 | 74548731 |
|  | chr15 | 75019442 | 75019501 |
|  | chr15 | 75337366 | 75337388 |
|  | chr15 | 75843698 | 75843730 |
|  | chr15 | 77393702 | 77393754 |
|  | chr15 | 80759602 | 80759696 |
|  | chr15 | 81390717 | 81390914 |
|  | chr15 | 84436607 | 84436738 |
|  | chr15 | 84482002 | 84482111 |
|  | chr15 | 85315450 | 85315507 |
|  | chr15 | 86927662 | 86927760 |
|  | chr15 | 87285578 | 87285847 |
|  | chr15 | 92531460 | 92531522 |
|  | chr15 | 93765105 | 93765212 |
|  | chr15 | 95197369 | 95197555 |
|  | chr15 | 95649673 | 95649832 |
|  | chr15 | 98793207 | 98793268 |
|  | chr15 | 100950756 | 100950823 |
|  | chr16 | 2917731 | 2917828 |
|  | chr16 | 4141866 | 4141918 |
|  | chr16 | 4821488 | 4821539 |
|  | chr16 | 4956504 | 4956520 |
|  | chr16 | 5737313 | 5737512 |
|  | chr16 | 5862505 | 5862547 |
|  | chr16 | 6037595 | 6037680 |
|  | chr16 | 6618829 | 6618875 |
|  | chr16 | 7106260 | 7106277 |
|  | chr16 | 7433852 | 7434017 |
|  | chr16 | 7480387 | 7480492 |
|  | chr16 | 9038829 | 9038849 |
|  | chr16 | 11130694 | 11130728 |
|  | chr16 | 13204236 | 13204279 |
|  | chr16 | 13755592 | 13755696 |
|  | chr16 | 15066359 | 15066429 |
|  | chr16 | 16631236 | 16631527 |
|  | chr16 | 17238614 | 17238680 |
|  | chr16 | 18747538 | 18747586 |
|  | chr16 | 24063653 | 24063725 |
|  | chr16 | 24198207 | 24198371 |
|  | chr16 | 33947409 | 33947447 |
|  | chr16 | 47147922 | 47147999 |
|  | chr16 | 48834467 | 48834803 |
|  | chr16 | 49227159 | 49227226 |
|  | chr16 | 49402351 | 49402388 |
|  | chr16 | 51408940 | 51409018 |
|  | chr16 | 51439033 | 51439185 |
|  | chr16 | 51444755 | 51444850 |
|  | chr16 | 57335228 | 57335241 |
|  | chr16 | 59704488 | 59704688 |
|  | chr16 | 60566512 | 60566548 |
|  | chr16 | 60890528 | 60890672 |
|  | chr16 | 64758404 | 64758544 |
|  | chr16 | 69544073 | 69544141 |
|  | chr16 | 70416066 | 70416104 |
|  | chr16 | 70644681 | 70644758 |
|  | chr16 | 76322042 | 76322145 |
|  | chr16 | 78941736 | 78941773 |
|  | chr16 | 78946739 | 78946834 |
|  | chr16 | 79674160 | 79674381 |
|  | chr16 | 85150529 | 85150600 |
|  | chr16 | 87099441 | 87099528 |
|  | chr16 | 87871072 | 87871088 |
|  | chr16 | 87983365 | 87983405 |
|  | chr16 | 88013565 | 88013673 |
|  | chr16 | 88525813 | 88525902 |
|  | chr17 | 505327 | 505454 |
|  | chr17 | 609300 | 609397 |
|  | chr17 | 1457502 | 1457530 |
|  | chr17 | 3072518 | 3072577 |
|  | chr17 | 5886615 | 5886691 |
|  | chr17 | 5913976 | 5914053 |
|  | chr17 | 6066267 | 6066328 |
|  | chr17 | 6094005 | 6094041 |
|  | chr17 | 6145035 | 6145086 |
|  | chr17 | 10215751 | 10215946 |
|  | chr17 | 11235694 | 11235826 |
|  | chr17 | 11530779 | 11530911 |
|  | chr17 | 16761770 | 16761805 |
|  | chr17 | 17777140 | 17777193 |
|  | chr17 | 18015954 | 18015984 |
|  | chr17 | 25451897 | 25452032 |
|  | chr17 | 25773640 | 25773726 |
|  | chr17 | 25885987 | 25886127 |
|  | chr17 | 26880310 | 26880375 |
|  | chr17 | 27513128 | 27513188 |
|  | chr17 | 29648465 | 29648549 |
|  | chr17 | 32299789 | 32299847 |
|  | chr17 | 32520362 | 32520484 |
|  | chr17 | 35545388 | 35545411 |
|  | chr17 | 36182009 | 36182095 |
|  | chr17 | 37223931 | 37223953 |
|  | chr17 | 42186160 | 42186195 |
|  | chr17 | 45065429 | 45065489 |
|  | chr17 | 46697121 | 46697141 |
|  | chr17 | 48280685 | 48280708 |
|  | chr17 | 49517098 | 49517201 |
|  | chr17 | 49712729 | 49712747 |
|  | chr17 | 49788942 | 49789091 |
|  | chr17 | 51359384 | 51359456 |
|  | chr17 | 51477453 | 51477498 |
|  | chr17 | 51628255 | 51628314 |
|  | chr17 | 51880882 | 51881006 |
|  | chr17 | 52336975 | 52337062 |
|  | chr17 | 55516082 | 55516186 |
|  | chr17 | 55570633 | 55570736 |
|  | chr17 | 56422590 | 56422805 |
|  | chr17 | 56597031 | 56597073 |
|  | chr17 | 61539842 | 61539946 |
|  | chr17 | 65316899 | 65316919 |
|  | chr17 | 67946548 | 67946625 |
|  | chr17 | 69919034 | 69919218 |
|  | chr17 | 70191038 | 70191054 |
|  | chr17 | 76792682 | 76792907 |
|  | chr17 | 78042805 | 78042823 |
|  | chr17 | 78860136 | 78860218 |
|  | chr17 | 79129050 | 79129102 |
|  | chr17 | 79792008 | 79792058 |
|  | chr17 | 81103436 | 81103479 |
|  | chr18 | 380078 | 380214 |
|  | chr18 | 5123655 | 5123748 |
|  | chr18 | 5239001 | 5239087 |
|  | chr18 | 5363994 | 5364139 |
|  | chr18 | 6055976 | 6056148 |
|  | chr18 | 7056056 | 7056137 |
|  | chr18 | 10101377 | 10101633 |
|  | chr18 | 12651586 | 12651632 |
|  | chr18 | 13609065 | 13609112 |
|  | chr18 | 19320134 | 19320216 |
|  | chr18 | 20901876 | 20901967 |
|  | chr18 | 35082228 | 35082299 |
|  | chr18 | 39042490 | 39042523 |
|  | chr18 | 40861728 | 40861847 |
|  | chr18 | 41533971 | 41534082 |
|  | chr18 | 42161939 | 42161999 |
|  | chr18 | 43308151 | 43308205 |
|  | chr18 | 47519727 | 47519758 |
|  | chr18 | 48678702 | 48678730 |
|  | chr18 | 49081316 | 49081527 |
|  | chr18 | 52660602 | 52660666 |
|  | chr18 | 53349748 | 53349931 |
|  | chr18 | 60828218 | 60828431 |
|  | chr18 | 63069544 | 63069667 |
|  | chr18 | 63397724 | 63397794 |
|  | chr18 | 66905604 | 66905718 |
|  | chr18 | 68869292 | 68869492 |
|  | chr18 | 69995568 | 69995756 |
|  | chr18 | 70533988 | 70534013 |
|  | chr18 | 73756490 | 73756715 |
|  | chr18 | 73980434 | 73980491 |
|  | chr18 | 74146428 | 74146465 |
|  | chr18 | 75569488 | 75569532 |
|  | chr18 | 75779370 | 75779415 |
|  | chr18 | 77733926 | 77733986 |
|  | chr19 | 3312150 | 3312328 |
|  | chr19 | 5122684 | 5122701 |
|  | chr19 | 6091806 | 6091876 |
|  | chr19 | 8106297 | 8106405 |
|  | chr19 | 9512159 | 9512204 |
|  | chr19 | 10404100 | 10404135 |
|  | chr19 | 11736181 | 11736386 |
|  | chr19 | 12941220 | 12941271 |
|  | chr19 | 14341474 | 14341569 |
|  | chr19 | 14608935 | 14609015 |
|  | chr19 | 14649815 | 14649857 |
|  | chr19 | 16167974 | 16168153 |
|  | chr19 | 28935400 | 28935591 |
|  | chr19 | 29536443 | 29536643 |
|  | chr19 | 32240383 | 32240422 |
|  | chr19 | 39051579 | 39051747 |
|  | chr19 | 40293869 | 40293909 |
|  | chr19 | 44440463 | 44440573 |
|  | chr19 | 46179408 | 46179442 |
|  | chr19 | 47236842 | 47236949 |
|  | chr19 | 49106271 | 49106418 |
|  | chr19 | 50399909 | 50399989 |
|  | chr19 | 50946718 | 50946769 |
|  | chr19 | 51020070 | 51020085 |
|  | chr19 | 51581569 | 51581601 |
|  | chr19 | 54106762 | 54106792 |
|  | chr19 | 56675232 | 56675276 |
|  | chr19 | 57161797 | 57161834 |
|  | chr19 | 57389699 | 57389796 |
|  | chr20 | 1780545 | 1780596 |
|  | chr20 | 1978411 | 1978431 |
|  | chr20 | 3587151 | 3587213 |
|  | chr20 | 3837638 | 3837667 |
|  | chr20 | 3958488 | 3958508 |
|  | chr20 | 5276042 | 5276217 |
|  | chr20 | 5667227 | 5667419 |
|  | chr20 | 7986829 | 7986897 |
|  | chr20 | 9514046 | 9514071 |
|  | chr20 | 10982416 | 10982432 |
|  | chr20 | 11985909 | 11986112 |
|  | chr20 | 16024395 | 16024799 |
|  | chr20 | 16688703 | 16688788 |
|  | chr20 | 20203094 | 20203269 |
|  | chr20 | 20804457 | 20804509 |
|  | chr20 | 25845400 | 25845436 |
|  | chr20 | 25941788 | 25942024 |
|  | chr20 | 35408115 | 35408190 |
|  | chr20 | 36948810 | 36948857 |
|  | chr20 | 40053934 | 40054023 |
|  | chr20 | 40858797 | 40858909 |
|  | chr20 | 42733546 | 42733635 |
|  | chr20 | 43041002 | 43041174 |
|  | chr20 | 45401631 | 45401640 |
|  | chr20 | 45557711 | 45557766 |
|  | chr20 | 46486501 | 46486540 |
|  | chr20 | 47897999 | 47898032 |
|  | chr20 | 47970268 | 47970339 |
|  | chr20 | 48551697 | 48551709 |
|  | chr20 | 50317550 | 50317577 |
|  | chr20 | 52467034 | 52467062 |
|  | chr20 | 52539751 | 52539807 |
|  | chr20 | 52715103 | 52715134 |
|  | chr20 | 56327117 | 56327197 |
|  | chr20 | 57900458 | 57900479 |
|  | chr20 | 60209053 | 60209122 |
|  | chr20 | 60286209 | 60286322 |
|  | chr21 | 9948798 | 9948970 |
|  | chr21 | 17394461 | 17394557 |
|  | chr21 | 20725219 | 20725281 |
|  | chr21 | 21383462 | 21383534 |
|  | chr21 | 24019248 | 24019292 |
|  | chr21 | 24297290 | 24297369 |
|  | chr21 | 25680243 | 25680277 |
|  | chr21 | 26960348 | 26960364 |
|  | chr21 | 27431212 | 27431272 |
|  | chr21 | 29519723 | 29519778 |
|  | chr21 | 31708119 | 31708187 |
|  | chr21 | 34940563 | 34940576 |
|  | chr21 | 35501627 | 35501645 |
|  | chr21 | 37277517 | 37277709 |
|  | chr21 | 38381081 | 38381182 |
|  | chr21 | 38750995 | 38751180 |
|  | chr21 | 41342105 | 41342187 |
|  | chr21 | 42229643 | 42229775 |
|  | chr21 | 43681225 | 43681272 |
|  | chr21 | 44220213 | 44220253 |
|  | chr21 | 44577060 | 44577098 |
|  | chr21 | 46821020 | 46821040 |
|  | chr21 | 47896220 | 47896372 |
|  | chr22 | 18403096 | 18403152 |
|  | chr22 | 22346970 | 22347162 |
|  | chr22 | 25628605 | 25628617 |
|  | chr22 | 26771080 | 26771117 |
|  | chr22 | 29593650 | 29593694 |
|  | chr22 | 33467940 | 33468097 |
|  | chr22 | 34137139 | 34137167 |
|  | chr22 | 34568941 | 34568997 |
|  | chr22 | 35752798 | 35752858 |
|  | chr22 | 36115036 | 36115063 |
|  | chr22 | 36599678 | 36599853 |
|  | chr22 | 36753260 | 36753429 |
|  | chr22 | 37024474 | 37024525 |
|  | chr22 | 37088239 | 37088323 |
|  | chr22 | 37209839 | 37209861 |
|  | chr22 | 39028083 | 39028170 |
|  | chr22 | 43029136 | 43029152 |
|  | chr22 | 45609401 | 45609424 |
|  | chr22 | 46873283 | 46873379 |
|  | chr22 | 47082805 | 47082847 |
|  | chr22 | 49422142 | 49422166 |
|  | chr22 | 49435140 | 49435259 |
|  | chr22 | 50847659 | 50847678 |
|  | chrX | 515853 | 515973 |
|  | chrX | 1620129 | 1620189 |
|  | chrX | 1739912 | 1739951 |
|  | chrX | 1814789 | 1814909 |
|  | chrX | 2043822 | 2043846 |
|  | chrX | 4119429 | 4119571 |
|  | chrX | 6764374 | 6764401 |
|  | chrX | 9115662 | 9115707 |
|  | chrX | 12658187 | 12658314 |
|  | chrX | 13227241 | 13227383 |
|  | chrX | 13713195 | 13713233 |
|  | chrX | 13779959 | 13780013 |
|  | chrX | 14362009 | 14362062 |
|  | chrX | 18214183 | 18214278 |
|  | chrX | 18843351 | 18843486 |
|  | chrX | 19191892 | 19191962 |
|  | chrX | 19444826 | 19444893 |
|  | chrX | 19758922 | 19758957 |
|  | chrX | 23343284 | 23343305 |
|  | chrX | 24300744 | 24300929 |
|  | chrX | 25152652 | 25152785 |
|  | chrX | 27519060 | 27519106 |
|  | chrX | 27806727 | 27806876 |
|  | chrX | 29620202 | 29620394 |
|  | chrX | 30110549 | 30110604 |
|  | chrX | 31966149 | 31966318 |
|  | chrX | 35118520 | 35118669 |
|  | chrX | 39009605 | 39009777 |
|  | chrX | 41094164 | 41094447 |
|  | chrX | 41449211 | 41449259 |
|  | chrX | 44891941 | 44891978 |
|  | chrX | 46145756 | 46145799 |
|  | chrX | 56024385 | 56024463 |
|  | chrX | 57905072 | 57905222 |
|  | chrX | 63615379 | 63615419 |
|  | chrX | 64919436 | 64919529 |
|  | chrX | 67906525 | 67906684 |
|  | chrX | 68217388 | 68217465 |
|  | chrX | 68924789 | 68924962 |
|  | chrX | 68939842 | 68939890 |
|  | chrX | 69700733 | 69700856 |
|  | chrX | 70711985 | 70712024 |
|  | chrX | 70712928 | 70712974 |
|  | chrX | 73513090 | 73513146 |
|  | chrX | 74300207 | 74300433 |
|  | chrX | 82967711 | 82967769 |
|  | chrX | 83625208 | 83625280 |
|  | chrX | 92084856 | 92084913 |
|  | chrX | 94710104 | 94710259 |
|  | chrX | 96418311 | 96418411 |
|  | chrX | 100074535 | 100074606 |
|  | chrX | 100419444 | 100419570 |
|  | chrX | 103021835 | 103021870 |
|  | chrX | 106046600 | 106046661 |
|  | chrX | 107137171 | 107137209 |
|  | chrX | 107930700 | 107930914 |
|  | chrX | 109424267 | 109424279 |
|  | chrX | 110008131 | 110008224 |
|  | chrX | 110319485 | 110319674 |
|  | chrX | 111484055 | 111484073 |
|  | chrX | 116002579 | 116002681 |
|  | chrX | 119078079 | 119078098 |
|  | chrX | 123332017 | 123332117 |
|  | chrX | 124358672 | 124358747 |
|  | chrX | 124384234 | 124384319 |
|  | chrX | 125242174 | 125242240 |
|  | chrX | 131597209 | 131597221 |
|  | chrX | 136647256 | 136647371 |
|  | chrX | 136758937 | 136758979 |
|  | chrX | 137298871 | 137299098 |
|  | chrX | 139111820 | 139111965 |
|  | chrX | 141029779 | 141029904 |
|  | chrX | 155176142 | 155176182 |
